# Dissecting and analyzing the Subclonal Mutations Associated with Poor Prognosis in Diffuse Glioma

**DOI:** 10.1155/2022/4919111

**Published:** 2022-04-18

**Authors:** Ming Bai, Xiaolong Wang, Huixue Zhang, Jianjian Wang, Gaysina Lyaysan, Si Xu, Kuo Tian, Tianfeng Wang, Jie Li, Na Wang, Xiaoyu Lu, Xiaoming Zhang, Lihua Wang

**Affiliations:** ^1^The Second Affiliated Hospital of Harbin Medical University, Harbin, China; ^2^The Third Affiliated Hospital of Harbin Medical University, Harbin, China; ^3^BSMU Bashkir State Medical University, Ufa, Russia

## Abstract

The prognostic and therapeutic implications in diffuse gliomas are still challenging. In this study, we first performed an integrative framework to infer the clonal status of mutations in glioblastomas (GBMs) and low-grade gliomas (LGGs) by using exome sequencing data from TCGA and observed both clonal and subclonal mutations for most mutant genes. Based on the clonal status of a given gene, we systematically investigated its prognostic value in GBM and LGG, respectively. Focusing on the subclonal mutations, our results showed that they were more likely to contribute to the poor prognosis, which could be hardly figured out without considering clonal status. These risk subclonal mutations were associated with some specific genomic features, such as genomic instability and intratumor heterogeneity, and their accumulation could enhance the prognostic value. By analyzing the regulatory mechanisms underlying the risk subclonal mutations, we found that the subclonal mutations of AHNAK and AHNAK2 in GBM and those of NF1 and PTEN in LGG could influence some important molecules and functions associated with glioma progression. Furthermore, we dissected the role of risk subclonal mutations in tumor evolution and found that advanced subclonal mutations showed poorer overall survival. Our study revealed the importance of clonal status in prognosis analysis, highlighting the role of the subclonal mutation in glioma prognosis.

## 1. Introduction

Diffuse glioma is the most common aggressive primary brain tumor. It can be categorized into grade II, grade III, and grade IV (following the World Health Organization (WHO) classification) depending on the degree of aggressiveness [1]. As the most aggressive malignant glioma, glioblastoma multiforme (GBM, grade IV) shows a 5-year survival rate of 5% with the median overall survival of 14-17 months from diagnosis [2, 3]. While gliomas of grade II and III are less aggressive and have been grouped together by The Cancer Genome Atlas (TCGA) as lower grade gliomas (LGGs). However, this subtype classification is highly interobserver variable, and the survival varied considerably within grades [4, 5]. To understand the etiology of glioma, genetic alterations in tumor had been screened in large cohorts of patients previously [6, 7]. These large-scale studies reveal that the genetic landscape of human cancers is driven by the stepwise accumulation of somatic alterations, which is an intrinsic aspect of cancer development [8, 9]. The analysis of the genetic and clinical observations revealed that some actionable driver mutations could promote cancer progression and impact on patient outcome, such as the most commonly altered genes IDH, TP53, and PTEN [7, 10, 11].

Accumulating evidence suggests that diffuse gliomas are highly heterogeneous and invasive and have startling intratumor heterogeneity (ITH) [12, 13]. It has been proved that tumorigenesis progresses through a series of mutational events, providing putative markers for tumor progression [9, 14]. Some of these events are incremental, and others can be catastrophic and may impact on clinical outcome in glioma. Indeed, individual tumors likely evolve through exclusion and interaction between diverse genetic clones and may comprise of multiple clones that exhibit specifically important clinical implications [15]. Previous studies have demonstrated the efficacy of reconstructing the clonal architecture (including clonal and subclonal events) of individual tumor in clinical research [16, 17]. Clonal events (i.e., clonal or trunk mutations) harbor mutations common to all tumor cells. And subclonal events (i.e., subclonal or branch mutations) are specific to one evolutionary branch of the tumor and present in only a subset of cancer cells. Some studies have revealed that the presence of genetically distinct subclones and the clonal status of some genes may reduce the clinical benefit of cancer therapies [18, 19]. For instance, subclonal RAS mutations in colorectal cancer have been shown to precipitate resistance to cetuximab, indicating the importance of clonal evolution in drug resistance as well as the clinical outcome [20]. Francis et al. effectively identified clonal events in GBM and revealed subclonal diversity of oncogenic EGFR and its implication in resistance to treatment for EGFR inhibitors, revealing the broad implications of clonal status in resistance to treatment [21]. Recently, Luo et al. proved that integrating the clonal status into classification could provide more precise stratification in diffuse gliomas and showed that gliomas with subclonal IDH mutation and without 1p/19q codeletion could be a novel subgroup and significantly correlated with patients' clinical outcomes [19]. Therefore, to further investigate the impact of genetic mutations on clinical assessment and disease severity, a better understanding of the clonal status of mutation events is required.

In this study, we investigated the mutation clonality of all mutant genes and their clinical impact in GBM and LGG using the published large-scale genomic data from TCGA. Our results revealed the clonal status of each mutant gene and proved their prognostic value, especially for the risk subclonal mutations. Patients with subclonal mutations that associated with worse overall survival exhibited some interesting genomic and regulatory features, suggesting the clinical importance of considering clonal status in the treatment of gliomas.

## 2. Materials and Methods

### 2.1. Data Source

The clinical data and the somatic mutation data (exome sequencing data (level 2) and Affymetrix SNP6 data (level 3)) of 380 GBM samples and 499 LGG samples were obtained from TCGA and Broad Institute Firehose (https://gdac.broadinstitute.org). The variant allele frequencies of mutations, copy number aberrations, and tumor purity estimated by ABSOLUTE were obtained from cBioPortal (http://www.cbioportal.org) and TCGA's PanCancer Atlas (https://gdc.cancer.gov/about-data/publications/pancanatlas). The mutation burden data were downloaded from https://gdc.cancer.gov/about-data/publications/PanCan-CellOfOrigin. The mRNA and miRNA expression data for the LGG and GBM cohorts were collected from the TCGA data portal (https://tcga-data.nci.nih.gov/tcga), and the lncRNA expression data were derived from TANRIC [22]. Genes with mean expression (normalized by log2(tpm + 1)) lower than 30% of samples or with missing values in more than 10% of samples were filtered.

### 2.2. Inferring the Mutation Status of SNV

We used the tumor purity and the local copy number of mutation sites summarized by McGranahan et al. [23] and Landau et al. [16]. The cancer cell fraction (CCF) of each mutation was estimated by incorporating tumor purity, absolute somatic copy number, and variant allele frequency (VAF), and then, the clonal status of all gene mutations in each sample was inferred [8]. In brief, given a certain CCF of one mutation, the expected VAF could be calculated according to the following equation:

(1) $$\begin{array}{c} {\text{VAF}}_{\text{ex}}=\frac{\rho *\text{CCF}*{\text{CN}}_{\text{mut}}}{{\text{CPN}}_{\mathrm{norm}}\left(1-\rho \right)+\rho *\text{CPN}}, \end{array}$$

where *ρ* is the tumor purity, CN_mut_ denotes the copy number of the mutation in the cells where the mutation occurred (i.e., the number of chromosomal copies that carried the mutation), and CPN_norm_ and CPN_mut_ denote the absolute copy number of this locus in normal and tumor, respectively. Generally, the mutation was very difficult to occur at two or more alleles at the same site, so CN_mut_ was assumed to be 1 to avoid overcalling subclonal mutation [19, 23, 24]. CPN_norm_ was set to be 2 for autosomal chromosomes (mutations in the X and Y chromosome were not considered), and CPN_mut_ was estimated by ABSOLUTE. Therefore, the expected VAF can also be represented as follows:

(2) $$\begin{array}{c} {\text{VAF}}_{\text{ex}}=\frac{\rho *\text{CCF}}{2\left(1-\rho \right)+\rho *{\text{CPN}}_{\text{mut}}}. \end{array}$$

We then estimated the probability of a given CCF by using Bayesian probability theory and a binomial distribution:

(3) $$\begin{array}{c} P\left(\text{CCF}\;|\;\left(a\;|\;N\right)\right)=\frac{P\left(\left(a\;|\;N\right)\;|\;\text{CCF}\right)*P\left(\text{CCF}\right)}{P\left(a\;|\;N\right)},\\ P\left(\text{CCF}\;|\;\left(a\;|\;N\right)\right)\propto \text{Binom}\left(a\;|\;N,\text{VA}{\text{F}}_{\text{ex}}\left(\text{CCF}\right)\right), \end{array}$$

where *a* means the number of altered read counts for the mutation and *N* means the sequencing coverage *N* (alternate read count+reference read count).

Based on the posterior probability distribution of CCF, we obtained the estimated CCF and the 95% CI for each somatic mutation. Finally, we regarded mutations as clonal if the upper band of the CI95 was ≥1 and the Pr(clonal) > 0.5 and as subclonal otherwise. To reduce the background noise, we only analyzed the mutation status of genes with mutation frequency of 2% or greater. If a gene harbored multiple nonsilent SNVs in a patient, it was excluded.

### 2.3. Evaluating the Impact of Mutational Status on Patient Survival

The patients were classified as clonal, subclonal, and wild-type (WT) groups according to the mutation status of a given gene. We only considered genes that harbored both clonal and subclonal mutations in more than 5 patients, respectively. The corresponding clinical information (including clinicopathological factors and overall survival) of GBM and LGG patients were obtained as described above. To assess the associations between the mutational status and patient survival, the overall survival was used as the endpoint of this study, the Kaplan-Meier method was performed for the visualization, and the differences between survival curves (i.e., clonal mutation vs. WT and subclonal mutation vs. WT) were calculated by the log-rank test. The mutational statuses with *p* values smaller than 0.05 were considered to have a significant impact on patient survival. In addition, the univariate Cox proportional hazard regression analysis was also applied to evaluate the prognostic capability of mutational status. The mutational statuses with *p* values < 0.05 were considered to be significantly related to OS. In order to exclude the influence of large differences in the number of samples, we used the R package powerSurvEpi to calculate the power in the analysis of survival data to detect the survival differences under the corresponding sample size. To ensure the accuracy of the result, we filtered clonal and subclonal mutations with power < 80%.

### 2.4. Statistical Analysis of Clinical Data

The overall survival (OS) curves were constructed by the Kaplan-Meier estimation, with *p* values calculated by log-rank test. The univariate and multivariate Cox proportional hazard regression models were used to investigate the association between clonal/subclonal gene mutations and OS. A *p* value less than 0.05 was considered statistically significant. The relationship between genomic characteristics of different sample groups was assessed by using the Wilcoxon rank-sum test. All statistical analyses were performed with R statistical software (http://www.R-project.org).

### 2.5. Identification of Dysregulated Regulatory Interactions Driven by Risk Subclonal Mutation

We developed a computational strategy to identify dysregulated regulatory interactions driven by risk subclonal mutations. It consisted two main steps. The first step is calculating the difference of the expression correlation of the regulatory interactions between patients with the subclonal mutation and not to determine the extent of dysregulation. The extent of dysregulation was defined as follows:

(4) $$\begin{array}{c} ∆R=\left|{\text{cor}}_{v}\left(\text{gene}1,\text{gene}2\right)-{\text{cor}}_{n}\left(\text{gene}1,\text{gene}2\right)\right|, \end{array}$$

where cor_*v*_(gene1, gene2) was the Pearson correlation coefficient (PCC) estimated from the risk subclonal mutation patients and cor_*n*_(gene1, gene2) was from others. The second step is identifying the dysregulated regulatory interactions driven by risk subclonal mutations. Permutation test was performed to determine whether ∆*R* was statistically significant. We randomized the labels of mutation status 1000 times and recalculated the changes of correlation coefficients of each gene pair. A *p* value of 0.05 was used as the cut-off to obtain significantly dysregulated interactions.

### 2.6. Identification of ceRNA Triplets in GBM and LGG

The interactions of mRNA-miRNA and lncRNA-miRNA were obtained from StarBase v2.0 [25]. Using the expression profiles of mRNA, lncRNA, and miRNA in GBM and LGG, respectively, we calculated PCC between mRNA/lncRNA (ceRNA) and miRNA to measure their expression correlations. We required that the ceRNA pairs showed significantly positive correlations (adjusted *p* value < 0.05) in which the correlation of each miRNA-ceRNA pair should be significantly negative (adjusted *p* value < 0.05). The ceRNA pairs that passed these conditions were considered as candidate ceRNA triplets.

## 3. Results

### 3.1. Inference of Clonal Status of Somatic Mutations in Diffuse Gliomas

**Figure 1:**
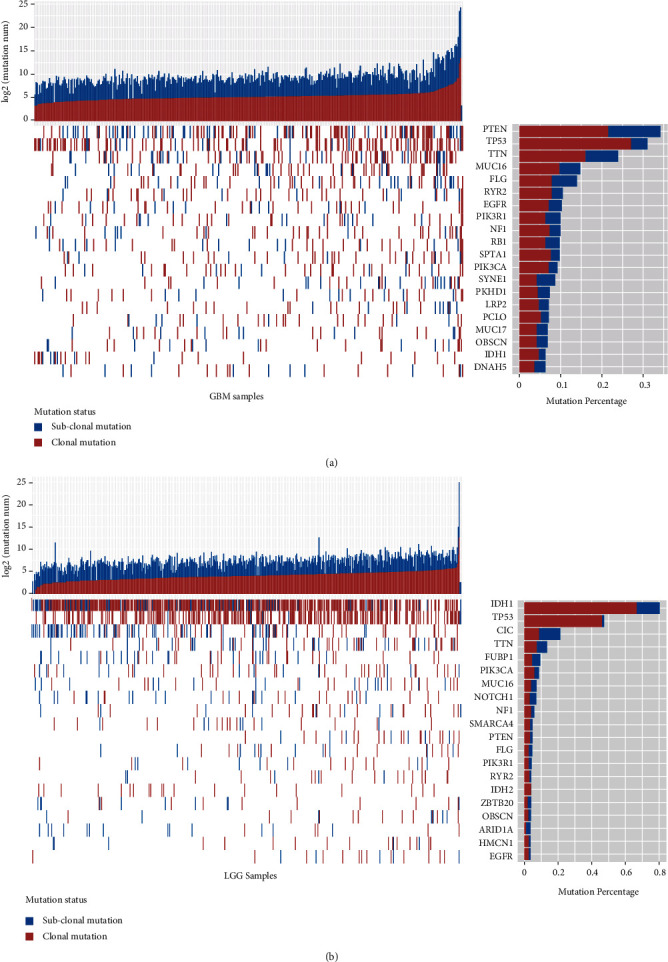
The mutation status of frequently altered driver genes in diffuse gliomas. The prevalence of clonal (red) and subclonal (blue) mutations (top) and the mutation status of each selected driver gene (middle and right) in GBM (a) and LGG (b).

The genomic data of 380 GBM samples and 499 LGG samples were obtained from TCGA. After preprocessing and filtering, over 77 thousand somatic nonsilent mutations were kept for subsequent analysis (see Materials and Methods). We adopted an integrated approach to estimate the cancer cell fraction (CCF) of each single nucleotide variation (SNV) in each sample and inferred clonal status of somatic mutations (see Materials and Methods). We identified 34,549 clonal mutations and 14,571 subclonal mutations in GBM and 16,504 clonal mutations and 11,960 subclonal mutations in LGG, respectively. As expected, the number of clonal mutations was generally higher than that of subclonal mutations within a sample in both GBM and LGG (Figure 1). In GBM, patients harbored an average of 91 and 39 clonal and subclonal mutations, respectively. And in LGG, the numbers were 32 and 23. After filtering out genes mutated in less than 2% sample, most mutant genes occurred both clonal and subclonal mutations in glioma, indicating that subclonal mutation was a widespread phenomenon.

We found that driver genes predominantly occurred clonal mutations. For example, a large proportion of IDH1 mutations were clonal (75% in GBM and 83% in LGG), consistent with previous experiments that the mutations in IDH tended to be trunk events in the tumor initiation (Figure 1) [26]. The clonal status of TP53 mutations was almost clonal in both GBM and LGG, indicating that the mutations in TP53 gene appear to be early events in tumorigenesis (Figure 1) [27]. Interestingly, more than 50% of CIC and FUBP1 mutations were found to be subclonal in LGG samples, suggesting that they probably occurred late during cancer evolution and played roles in tumor progression (Figure 1(b)).

### 3.2. The Mutation Status Could Be an Effective Prognostic Indicator in Diffuse Glioma

Different mutation status of some driver genes has been found to affect patient outcome in renal clear cell carcinoma, chronic lymphocytic leukemia, and breast cancer [18, 28, 29]. To evaluate the prognostic value of clonal status in diffuse glioma, we analyzed genes with both clonal and subclonal mutations which affected more than 5 patients (see Materials and Methods, Supplementary Table 1) and identified 3 genes whose clonal mutations and 4 genes whose subclonal mutations significantly affected overall survival in GBM (Table 1). The numbers in LGG were 5 and 6, respectively. The power for clonal status to detect significant survival differences under the corresponding sample size ranged from 81.1% to 100.0% in GBM and above 90% in LGG. We found that the mutation sites between the two clonal statuses were almost different, but they usually affected similar protein domains (Supplementary Figure 1). For example, the clonal and subclonal mutation sites of AHNAK2 had no interaction, but they were located in the same protein domains (Supplementary Figure 1C). Furthermore, we used two methods (SIFT [30] and POLYPHEN [31]) to predict the functional effect of each mutation and found that the proportion of the subclonal mutations predicted to damage protein function was comparable with clonal mutations (42% and 44%, respectively). These results indicated that not all mutations in a gene have an equal impact, and other factors needed to be further considered to reveal the effect of the clonal status on prognosis [32].

**Figure 2:**
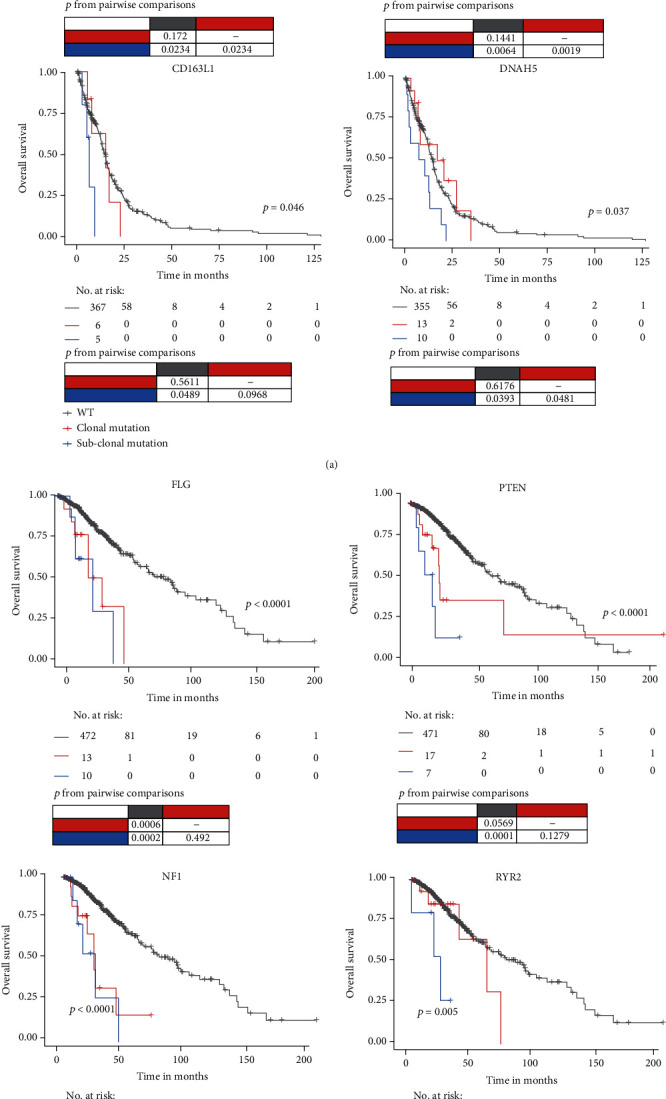
Overall survival among GBM and LGG patients stratified by subclonal mutation. Kaplan-Meier estimates overall survival in GBM (a) and LGG (b) patients harboring risk subclonal mutation.

For some well-known cancer genes, such as TP53 and IDH1 in GBM and EGFR in LGG, their clonal mutations showed improved prognosis in survival, which was consistent with previous studies (Table 1). Notably, all of the subclonal mutations in GBM (DNAH5, AHNAK, AHNAK2, and CD163L1, Figure 2(a)) and most of the subclonal mutations in LGG (PTEN, RYR2, NF1, and FLG, Figure 2(b)) showed significantly poor prognosis, suggesting that subclonal mutation preferred to be a risk factor.

To further evaluate the necessity of mutation status in prognosis analysis, we integrated the Kaplan-Meier method and log-rank test to distinguish the prognostic impact of mutation and mutation status. Our results showed that the prognostic effect of most genes with risk or protect clonal mutation could be identified by overall mutation, such as TP53 and IDH1 in GBM and EGFR, IDH1, NF1, and FLG in LGG (Supplementary Figure 2). However, the prognostic effect of genes with subclonal mutations could be barely recognized by overall mutation. In GBM, all of the genes with risk subclonal mutation had no prognostic significance when just used the mutation of the gene. These findings suggested that the absence of the reference for mutation status may reduce the accuracy of clinical guidance.

### 3.3. The Risk Subclonal Mutations Could Be Novel Prognostic Markers

**Figure 3:**
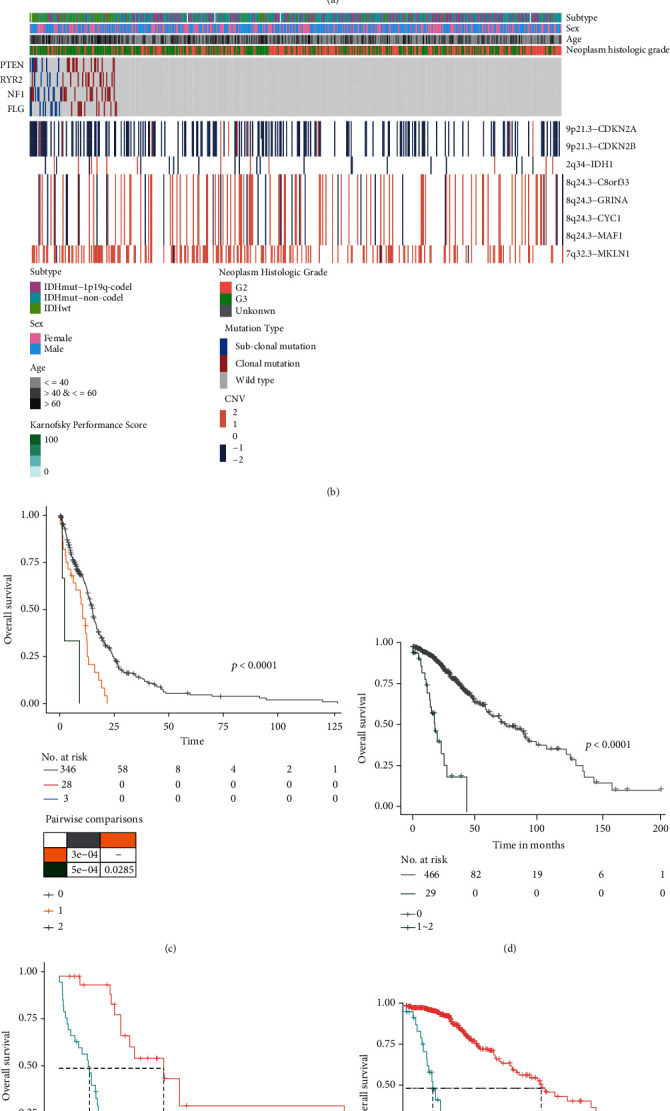
Overview of the clinical features of patients with risk subclonal mutation. (a and b) The heatmap displays the main copy number variations and clinical features in GBM (a) and LGG (b). (c and d) Kaplan-Meier survival curves of the patients without or with at least one or two risk subclonal mutations in GBM (c) and LGG (d). (e and f) Kaplan-Meier survival curves of the patients with IDH mutation or risk subclonal mutation in GBM (e) and LGG (f).

As most of the subclonal mutations were risk prognostic factors in both GBM and LGG, we then focused on this prognostic type for the following analysis (Figure 3). Our results showed that risk subclonal mutation correlated with the deletion of 9p21.3 (which contained CDKN2A and CDKN2B) in gliomas, especially in LGG (Figures 3(a) and 3(b)). Patients with these risk subclonal mutations did not appear to contain IDH1 alterations and preferred to be IDH1 wild-type subtype (Figures 3(a) and 3(b)). We also found that the patients with the risk subclonal mutations had a significantly higher frequency of genetically altered canonical oncogenic signaling pathways, such as P53 signaling in GBM and PI3K signaling in LGG [33]. The accumulation of the risk subclonal mutations showed significantly poor outcome (HR: 2.3218 (1.641−3.2848) and *p* value < 0.0001 for GBM; HR: 3.542 (2.3895−5.2505) and *p* value < 0.0001 for LGG, univariate Cox regression analysis), and patients with more mutations tended to have worse overall survival (Figures 3(c) and 3(d)).

Previous studies have shown that IDH mutation status is a strong predictor of survival in gliomas, which are associated with improved survival compared with GBM [34, 35]. By considering clonal status, our results showed a consistent prognostic effect in both LGG and GBM (Table 1). It should be noted that the presence or absence of an IDH mutation has the largest prognostic significance (more strongly predicted OS than did histologic grade and other molecular alterations) [36]. When comparing with the risk subclonal mutations, we found that the presence of a subclonal mutation was an independent factor (*p* value = 0.0375 in GBM and 7.88e-06 in LGG, multivariate Cox regression analysis) and could separate patients with IDH mutation into two subgroups with significant differences in OS (*p* value < 0.001 in both GBM and LGG, log-rank test, Figures 3(e) and 3(f)). Our results showed that the risk subclonal mutation was able to improve the accuracy of prediction of OS based on IDH mutation, suggesting that assessment of subclonal mutation would be effective in conjunction with the current prognostic instruments to provide a more accurate prognosis.

### 3.4. Patients with Prognostic Subclonal Mutations Reflecting Worse Genomic Instability

**Figure 4:**
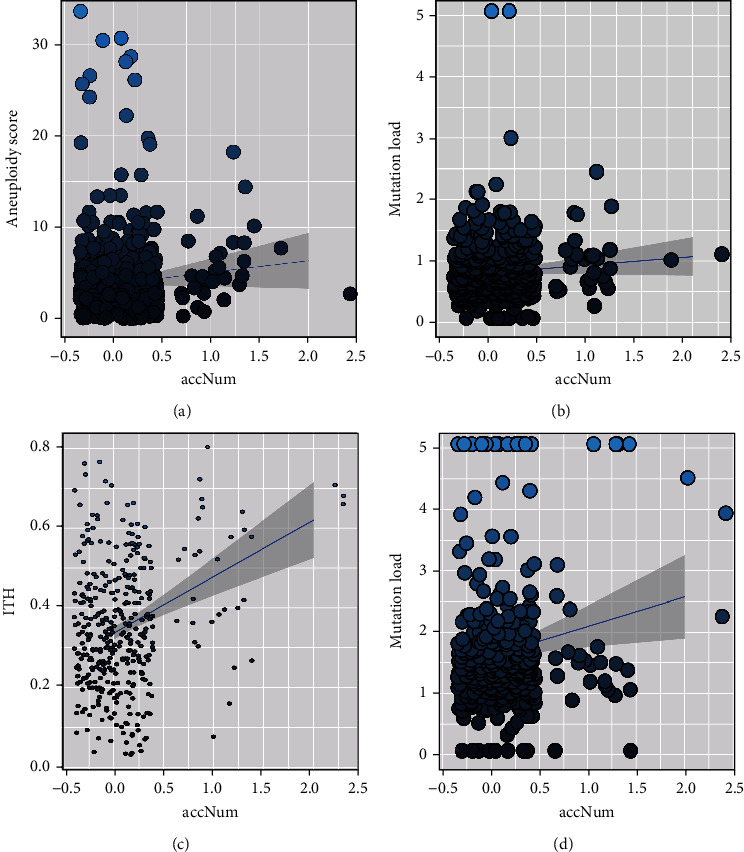
The association between the risk subclonal mutation and different genomic characteristics. The scatter plots represent the relationship between the accumulation of the risk subclonal mutations and aneuploidy score (a) or mutation load (b) in GBM and ITH (c) or mutation load (d) in LGG.

To investigate the potential mechanisms of risk subclonal mutations, we analyzed several genomic features of patients with or without these subclonal mutations. We found that patients with these subclonal mutations preferred to have higher aneuploidy scores than patients without them (*p* value = 0.00466, Wilcoxon rank-sum test, Figure 4(a)) and patients with prognostic clonal mutations (*p* value = 0.00136, Wilcoxon rank-sum test, Supplementary Figure 1D) in LGG. Patients with risk subclonal mutations also showed a positive correlation with elevated mutation load in both LGG and GBM (*p* value < 0.05, Wilcoxon rank-sum test, Figures 4(b) and 4(d) ), and the results were similar by comparing with patients with prognostic clonal mutations (*p* value = 0.0245 for LGG and 0.281 for GBM, Wilcoxon rank-sum test, Supplementary Figure 1E and 1F).

Previous studies have shown that genomic instability often leads to high diversity within tumors and this diversity is termed intratumor heterogeneity (ITH), which was a determinant of patient survival outcomes. We found that the patients with at least one subclonal mutation had significantly higher ITH than other patients as well as patients with prognostic clonal mutations in GBM (*p* values = 7.618e-06 and 4.64e-04, respectively, Wilcoxon rank-sum test, Figure 4(c) and Supplementary Figure 1G) and also showed a positive correlation in LGG. In addition, ITH alone may not be sufficient as a prognostic determinant, and patients with both high extent of ITH and subclonal mutations reflected worse overall survival. These results suggested that the risk subclonal mutations were correlated with specific biological mutagenesis mechanisms and genomic instability in glioma.

### 3.5. Exploring the Dysregulated Transcriptional Programs Driven by the Prognostic Subclonal Mutations

Next, we analyzed the regulatory differences driven by the risk subclonal mutations to explore their potential effect. We first investigated whether these subclonal mutations could influence transcription factor binding. Based on the transcriptional regulatory network from TRRUST v2 database (http://www.grnpedia.org/trrust), we designed a two-step strategy to identify dysregulated interactions of patients with the subclonal mutations: (1) calculated the degree of dysregulation based on the expression correlation and (2) used perturbation test to get significant dysregulated interactions. In total, 152 transcriptional regulatory relationships that significantly changed in GBM and 441 in LGG were identified (Supplementary Figure 3A and 3B). Using GSEA and MSigDB database, we found that the dysregulated interactions driven by the subclonal mutation were significantly enriched in regulation of cell population proliferation, regulation of RNA metabolic process, and regulation of cell death in both GBM and LGG (FDR < 0.001, Supplementary Figure 3C and 3D). The overlap between the top 10 enriched functions was 90%, suggesting their similar functions under risk subclonal mutations.

**Figure 5:**
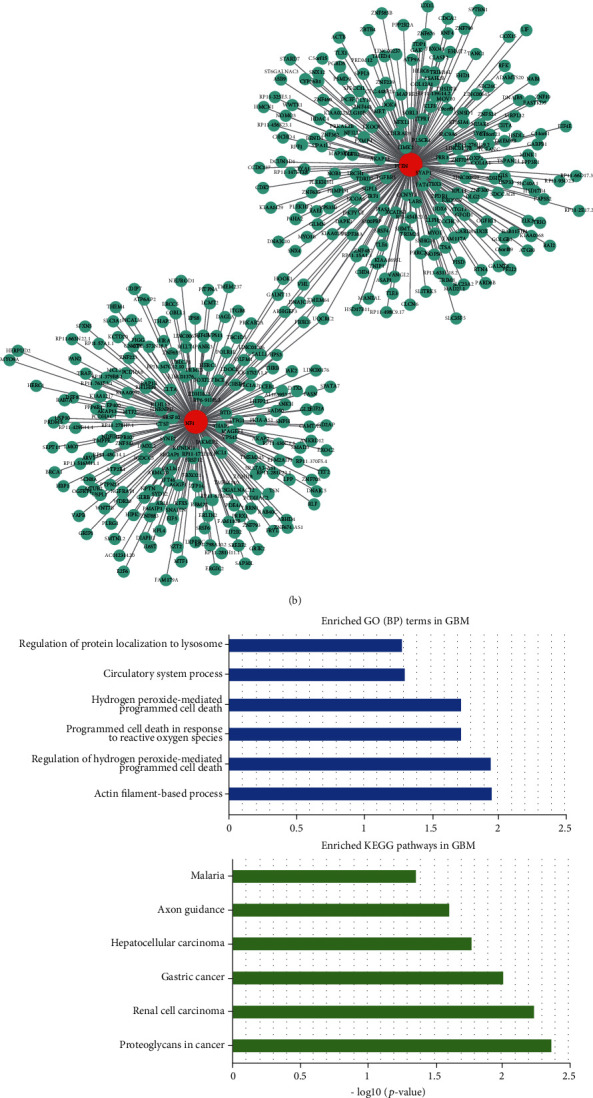
Dysregulated ceRNA pairs driven by the risk subclonal mutation. (a and b) Global view of ceRNA networks driven by subclonal mutations of AHNAK and AHNAK2 in GBM (a) and of PTEN and NF1 in LGG (b). (c and d) GO terms and KEGG pathways annotated by all dysregulated ceRNAs in GBM (c) and LGG (d).

We then constructed ceRNA network and identified dysregulated ceRNA networks driven by the subclonal mutations based on the above strategy and a previous study [37]. In GBM, the dysregulated ceRNA network driven by AHNAK and AHNAK2 were identified, including 66 and 16 significantly changed ceRNA pairs, respectively (Figure 5(a)). The dysregulated ceRNAs also contained 17 long noncoding RNAs (lncRNAs), such as MALAT1 and MIR22HG, which had been proved to play important roles in glioma [38, 39]. In LGG, a total of 766 dysregulated ceRNA pairs were identified (including 61 lncRNAs, such as SNHG16, ZNF883, and MIR22HG), which were driven by NF1 and PTEN (Figure 5(b)). Functional characterization of the dysregulated ceRNA networks revealed some biological pathways that are critical to tumor progression. In GBM, the dysregulated ceRNAs were primarily enriched in categories related to circulatory system process, programmed cell death and axon guidance, and proteoglycans in cancer pathways (Figure 5(c)). In LGG, the top significant biological functions were crucial to glioma development, including cell cycle, neuron development, neurogenesis, and neuron differentiation (Figure 5(d)). These results suggested that the risk subclonal mutations in GBM participated in more specific pathways than in LGG. For example, proteoglycans in cancer have been proved to be critical for understanding tumor microenvironment interactions and many signaling pathways [40, 41]. They have been proved to regulate multiple determinants of tumorigenesis in GBM, indicating a potential role of the subclonal mutations.

### 3.6. Further Classifying the Subclonal Mutations and Constructing Evolutionary Trees

Considering the evolutionary tree for each patient with prognostic subclonal mutations could further dissect the subclonal mutations. We reconstructed the evolutionary trees for most of these patients according to Nik-Zainal et al. (except 3 patients in LGG) [42]. The subclonal mutations located at the terminal subclone which also had mean CCF < 0.3 were regarded as advanced subclonal mutations (subclone 2). For each prognostic subclonal mutation, we compared the OS between patients with the subclonal mutation in subclone 1 and in subclone 2. Our results showed that 70% of them could be classified into two subgroups (except DNAH5 in GBM and RYR2 and PTEN in LGG). Most of the submutations classified as subclone2 showed poorer OS, such as AHNAK and AHNAK2 in GBM (Supplementary Figure 4A-C for AHNAK2) and CIC and FLG in LGG (Supplementary Figure 4D-F for CIC), further supporting the prognostic value of subclonal mutations.

## 4. Discussion

The genetic heterogeneity in glioma has been recently emphasized by whole-genome and exome sequencing studies. In this study, we observed that somatic mutations in many genes showed widespread clonal heterogeneity in GBM and LGG patients, including some driver genes, such as TP53, PTEN, and EGFR. More importantly, the mutation status of several genes was an independent predictor of patient survival. As expected, the prognostic mutation status of driver genes was mainly clonal mutation. And subclonal mutations preferred to contribute to poor prognosis, which could be hardly appropriately recognized without considering mutation status. We mainly analyzed the risk subclonal mutations and found that the accumulation of the mutations came with the increased genomic instability and ITH in both GBM and LGG. In addition, by analyzing the regulatory differences driven by the prognostic subclonal mutations, we identified some underlying biological pathways that contributed to the cancer progression, indicating the important roles of subclonal mutations in diffuse gliomas.

Our results identified clonal and subclonal mutation of many genes, indicating that the subclonal mutation was a widespread phenomenon in GBM and LGG. By classifying patients based on mutation status, we identified more prognostic factors than considering mutation only. Our results showed that for most driver genes, clonal mutation was the main prognostic factor, such as TP53 and IDH1 in GBM and EGFR in LGG, which had been proved by previous studies [43–45]. When both clonal and subclonal mutations were prognostic factors, they usually showed same effect. We also found that some genes, especially those whose only subclonal mutation affected overall survival, could be hardly identified by only mutation. For example, previous studies failed to show that PTEN mutation was linked to survival [46, 47]. Our results revealed that it was the subclonal mutation of PTEN that contributed to the prognosis in LGG.

As most prognostic subclonal mutations were risk factors, our study showed important insights into the genomic features of the risk subclonal mutation status. Previous studies had revealed that genomic instability often leads to high diversity within tumors [48]. Many previous studies have revealed that the extent of ITH was a potential determinant of patient survival outcomes [17, 49]. Our analysis suggested that the high extent of ITH was a common genomic character in the patients with risk subclonal mutation, highlighting the role of the risk subclonal mutation in influencing the OS of patients.

In GBM, we identified ceRNA triplets driven by the risk subclonal mutations of AHNAK and AHNAK2. It has been shown that the mRNA levels of AHNAK were downregulated in glioma and may be an independent prognostic factor for poor survival of glioma patients [50]. Our results demonstrated its prognostic value in genomic level, and its subclonal mutation could influence cell death program and axon guidance by ceRNA mechanisms. For AHNAK2, it has not been well studied in glioma, but it has been proved to be candidate cancer biomarker in pancreatic cancer and papillary thyroid carcinoma [51, 52]. In LGG, ceRNA triplets driven by the risk subclonal mutations of PTEN and NF1 were identified and shown to participate in neuron development, neurogenesis, and nervous system development. The functional enrichment results were consistent with previous studies that PTEN deletion could enhance constitutive neurogenesis and the inactivation of NF1 was important to central nervous system [53, 54]. We further did functional characterization of the dysregulated ceRNA networks driven by clonal mutations and found that the enriched GO terms were more likely to be parent terms of those in subclonal mutations, indicating a more specialized role of the subclonal mutations (Supplementary Figure 5). These results provided a potential explanation of the molecular mechanism of the risk subclonal mutations in diffuse gliomas.

## Figures and Tables

**Table 1:** Univariate and multivariate analysis of the prognostic clonal and subclonal mutations in GBM and LGG.

Cancer	Gene	Predictors	Univariate analysis		Multivariate analysis	
			HR (95% CI)	*p* value	HR (95% CI)	*p* value
GBM	TP53	Clonal mutation vs. WT	0.6236 (0.4737-0.8209)	8.00E-04	0.7207 (0.5468-0.9499)	0.0201
	IDH1	Clonal mutation vs. WT	0.3341 (0.1717-0.6501)	0.0012	0.5314 (0.2675-1.0554)	0.0709
	EPPK1	Clonal mutation vs. WT	3.3009 (1.5338-7.1036)	0.0023	3.5796 (1.6625-7.7075)	0.0011
	CD163L1	Subclonal mutation vs. WT	3.2253 (1.1868-8.7651)	0.0217	3.0238 (1.1119-8.2231)	0.0302
	DNAH5	Subclonal mutation vs. WT	2.1961 (1.1645-4.1415)	0.0151	1.8207 (0.9627-3.4437)	0.0653
	AHNAK	Subclonal mutation vs. WT	2.0811 (1.135-3.8161)	0.0178	1.6487 (0.8968-3.031)	0.1075
	AHNAK2	Subclonal mutation vs. WT	2.9256 (1.3667-6.2629)	0.0057	2.9638 (1.3828-6.3522)	0.0052

LGG	MUC17	Clonal mutation vs. WT	3.2943 (1.2059-8.9997)	0.0201	2.5295 (0.897-7.1326)	0.0793
	EGFR	Clonal mutation vs. WT	5.7694 (2.7781-11.9817)	<0.0001	2.2067 (1.0339-4.7102)	0.0408
	CIC	Subclonal mutation vs. WT	0.4035 (0.1966-0.828)	0.0133	0.3232 (0.1568-0.6661)	0.0022
	PTEN	Subclonal mutation vs. WT	7.3787 (2.9695-18.3348)	<0.0001	5.3941 (2.1497-13.5353)	3.00E-04
	RYR2	Subclonal mutation vs. WT	5.213 (1.6373-16.5981)	0.0052	5.8611 (1.8325-18.7458)	0.0029
	IDH1	Clonal mutation vs. WT	0.2127 (0.1441-0.3137)	<0.0001	0.3159 (0.2089-0.4777)	<0.0001
		Subclonal mutation vs. WT	0.2502 (0.1319-0.4748)	<0.0001	0.3178 (0.1648-0.613)	6.00E-04
	NF1	Clonal mutation vs. WT	4.029 (2.019-8.0403)	1.00E-04	3.1538 (1.5555-6.3942)	0.0014
		Subclonal mutation vs. WT	6.1097 (2.4638-15.1509)	1.00E-04	3.8465 (1.5393-9.6123)	0.0039
	FLG	Clonal mutation vs. WT	3.7363 (1.7226-8.1039)	8.00E-04	3.4509 (1.574-7.5658)	0.002
		Subclonal mutation vs. WT	5.219 (2.1026-12.954)	4.00E-04	3.655 (1.4599-9.1508)	0.0056

## Data Availability

The original contributions presented in the study are included in the article/supplementary material. Further inquiries can be directed to the corresponding authors.

## Supplementary Materials

Supplementary 1 Supplementary Figure 1: comparison analysis of the prognostic clonal mutations and risk subclonal mutations. (A) Density distribution of overlapping mutation sites between two clonal statuses. (B) Density distribution of overlapping protein domains affected by two clonal statuses. (C) Schematic diagram of AHNAK2. Protein domains are represented by boxes. Variants represented on the top of the protein correspond to clonal mutation sites whereas variants represented. (D-G) The scatter plots represent the difference of aneuploidy score (D), mutation load (E-F), and ITH (G) between patients with the risk subclonal mutations and prognostic clonal mutations. Supplementary Figure 2: overall survival among GBM and LGG patients stratified by overall mutation. (A) Overall survival in GBM patients harboring mutation of TP53 or IDH1. (B) Overall survival in LGG patients harboring mutation of EGFR, IDH1, NF1, or FLG. Supplementary Figure 3: transcriptional regulatory relationships influenced by the prognostic subclonal mutations in GBM and LGG. (A-B) Transcriptional regulatory relationships that significantly changed in GBM (A) and in LGG (B). (C-D) Functional enrichment results of dysregulated transcriptional regulatory networks in GBM (C) and in LGG (D). Supplementary Figure 4: evolutionary trees for patients with risk subclonal mutation. (A-C) Evolutionary trees for two patients with subclonal mutation of AHNAK2 (A-B) and survival comparison between subclones in GBM (C). (D-F) Evolutionary trees for two patients with subclonal mutation of CIC (D-E) and survival comparison between subclones in LGG (F). Supplementary Figure 5: GO graph displays the biological process involved in the clonal mutation (red) and the subclonal mutation (blue).

Supplementary 2 Supplementary Table 1: the clonal status of somatic mutations in GBM and LGG.

Supplementary 3 Supplementary Table 2: the dysregulated ceRNA networks driven by risk subclonal mutations in GBM and LGG.
