# Supplementary material for: Dissecting and analyzing the Subclonal Mutations Associated with Poor Prognosis in Diffuse Glioma
Source: Biomed Res Int. 2022 Apr 18;2022:4919111. doi: 10.1155/2022/4919111 (PMC9039777; doi:10.1155/2022/4919111)
Supplement: Supplementary 1 — Supplementary Figure 1: comparison analysis of the prognostic clonal mutations and risk subclonal mutations. (A) Density distribution of overlapping mutation sites between two clonal statuses. (B) Density distribution of overlapping protein domains affected by two clonal statuses. (C) Schematic diagram of AHNAK2. Protein domains are represented by boxes. Variants represented on the top of the protein correspond to clonal mutation sites whereas variants represented. (D-G) The scatter plots represent the difference of aneuploidy score (D), mutation load (E-F), and ITH (G) between patients with the risk subclonal mutations and prognostic clonal mutations. Supplementary Figure 2: overall survival among GBM and LGG patients stratified by overall mutation. (A) Overall survival in GBM patients harboring mutation of TP53 or IDH1. (B) Overall survival in LGG patients harboring mutation of EGFR, IDH1, NF1, or FLG. Supplementary Figure 3: transcriptional regulatory relationships influenced by the prognostic subclonal mutations in GBM and LGG. (A-B) Transcriptional regulatory relationships that significantly changed in GBM (A) and in LGG (B). (C-D) Functional enrichment results of dysregulated transcriptional regulatory networks in GBM (C) and in LGG (D). Supplementary Figure 4: evolutionary trees for patients with risk subclonal mutation. (A-C) Evolutionary trees for two patients with subclonal mutation of AHNAK2 (A-B) and survival comparison between subclones in GBM (C). (D-F) Evolutionary trees for two patients with subclonal mutation of CIC (D-E) and survival comparison between subclones in LGG (F). Supplementary Figure 5: GO graph displays the biological process involved in the clonal mutation (red) and the subclonal mutation (blue). [file 4919111.f1.pdf]

Supplementary Figures

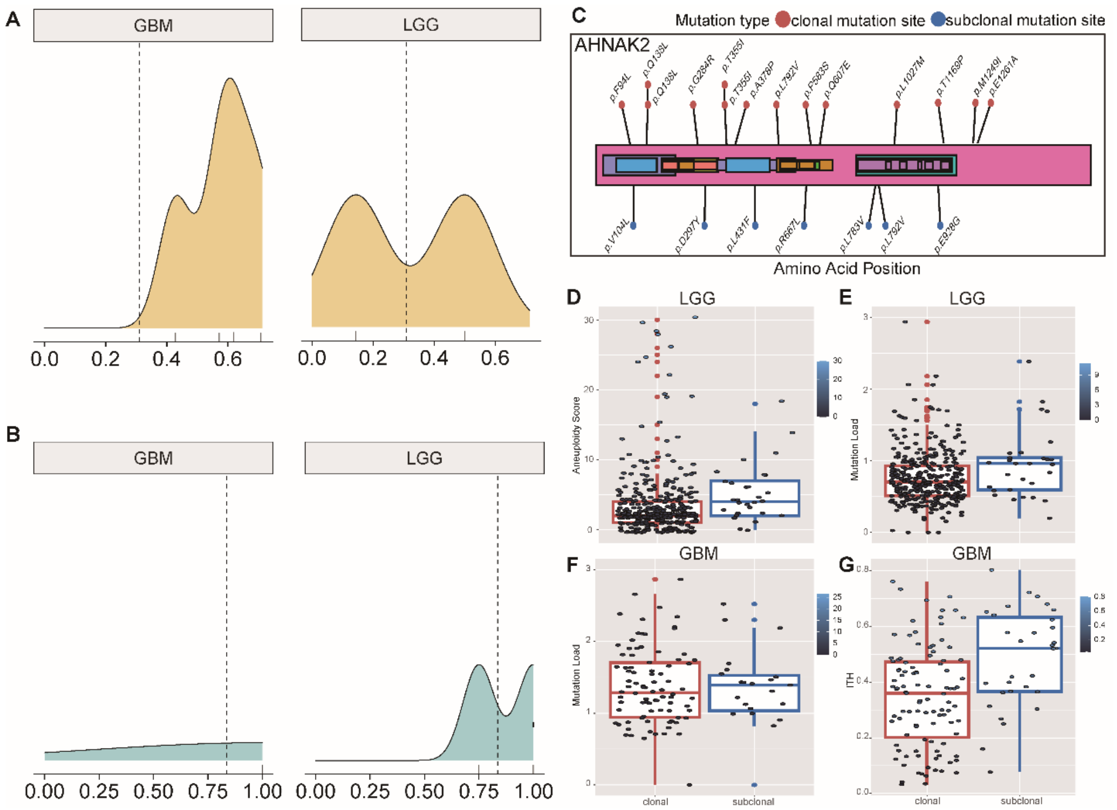

Supplementary Figure 1. Comparison analysis of the prognostic clonal mutations and risk sub-clonal mutations. (A) Density distribution of overlapping mutation sites between two clonal statuses. (B) Density distribution of overlapping protein domains affected by two clonal statuses. (C) Schematic diagram of AHNAK2. Protein domains are represented by boxes. Variants represented on the top of the protein correspond to clonal mutation sites whereas variants represented. (D-G) The scatter plots represent the difference of aneuploidy score (D), mutation load (E-F) and ITH (G) between patients with the risk sub-clonal mutations and prognostic clonal mutations.

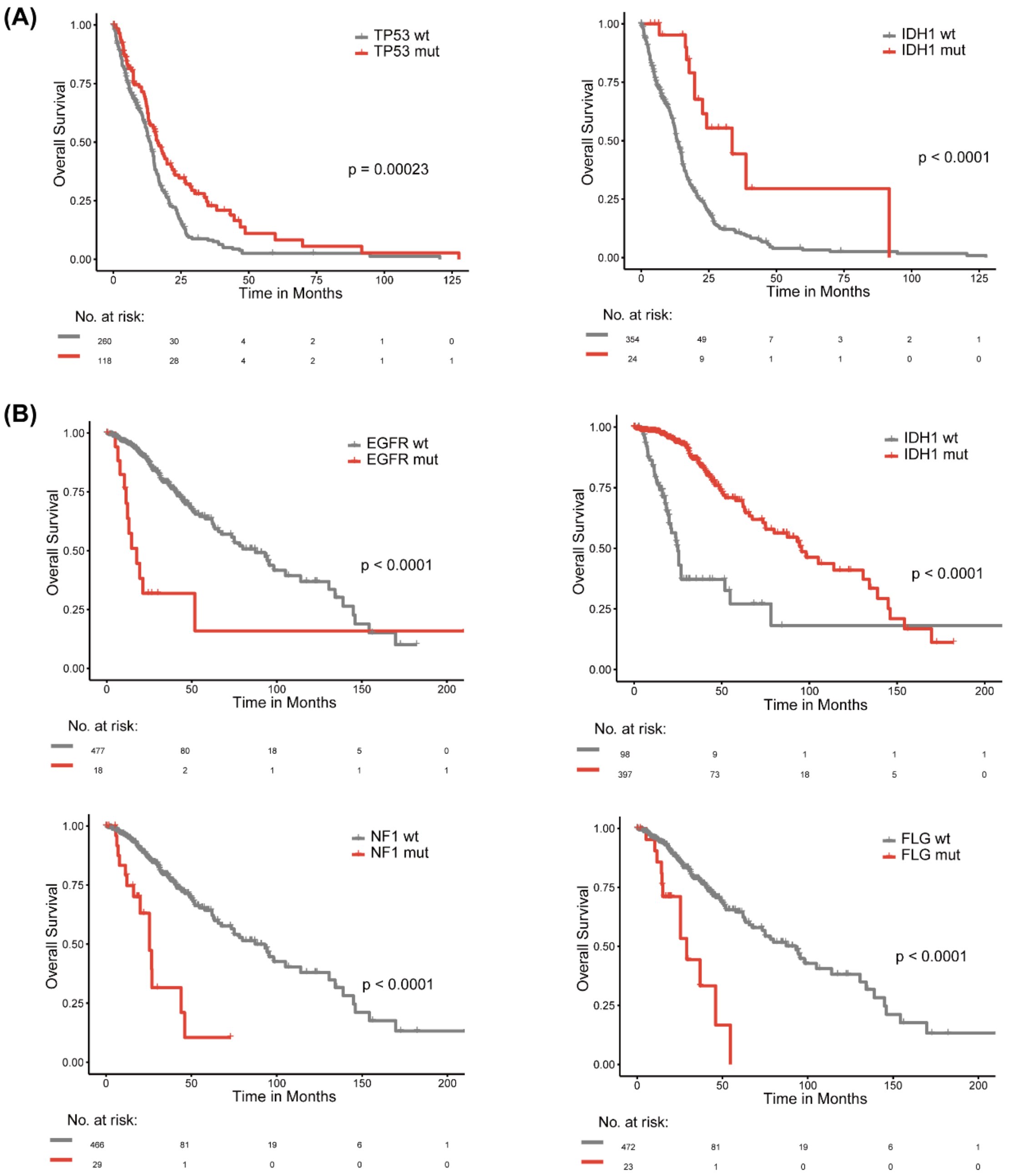

Supplementary Figure 2. Overall survival among GBM and LGG patients stratified by overall mutation. (A) Overall survival in GBM patients harboring mutation of TP53 or IDH1. (B) Overall survival in LGG patients harboring mutation of EGFR, IDH1, NF1 or FLG.

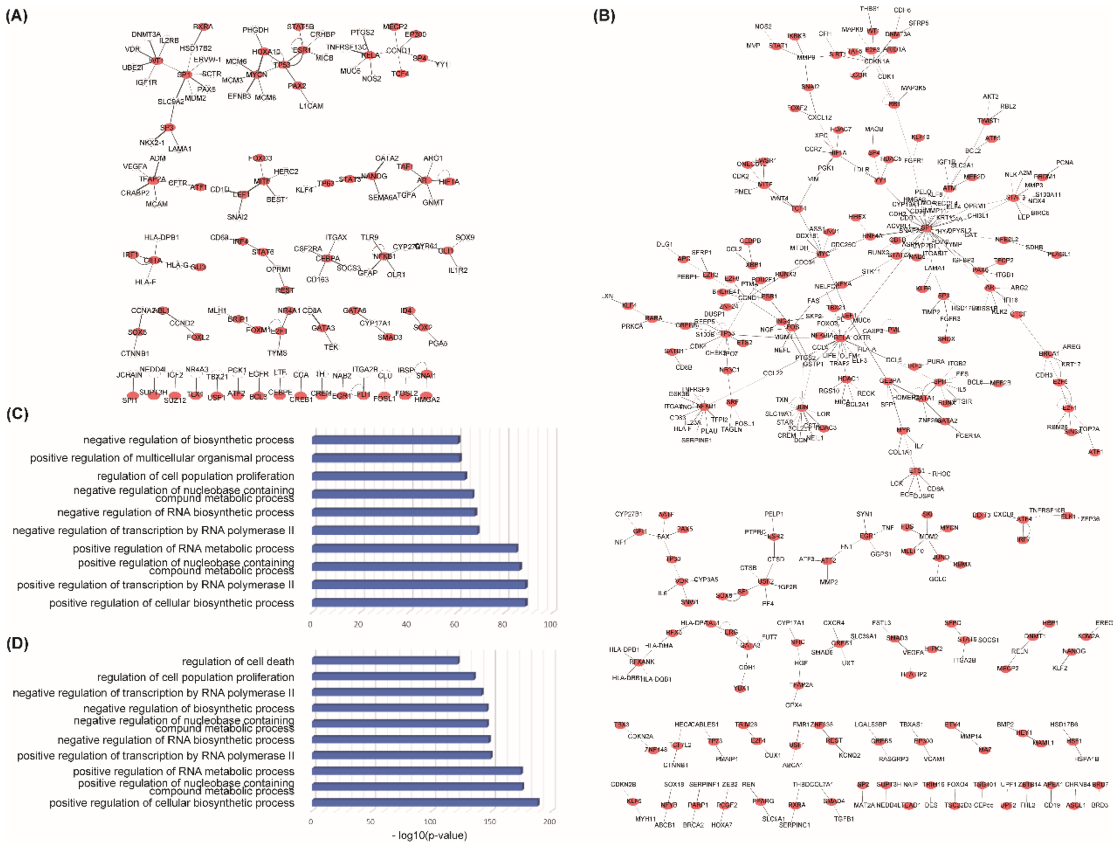

Supplementary Figure 3. Transcriptional regulatory relationships influenced by the prognostic sub-clonal mutations in GBM and LGG. (A-B) Transcriptional regulatory relationships that significantly changed in GBM (A) and in LGG (B). (C-D) Functional enrichment results of dysregulated transcriptional regulatory networks in GBM (C) and in LGG (D).

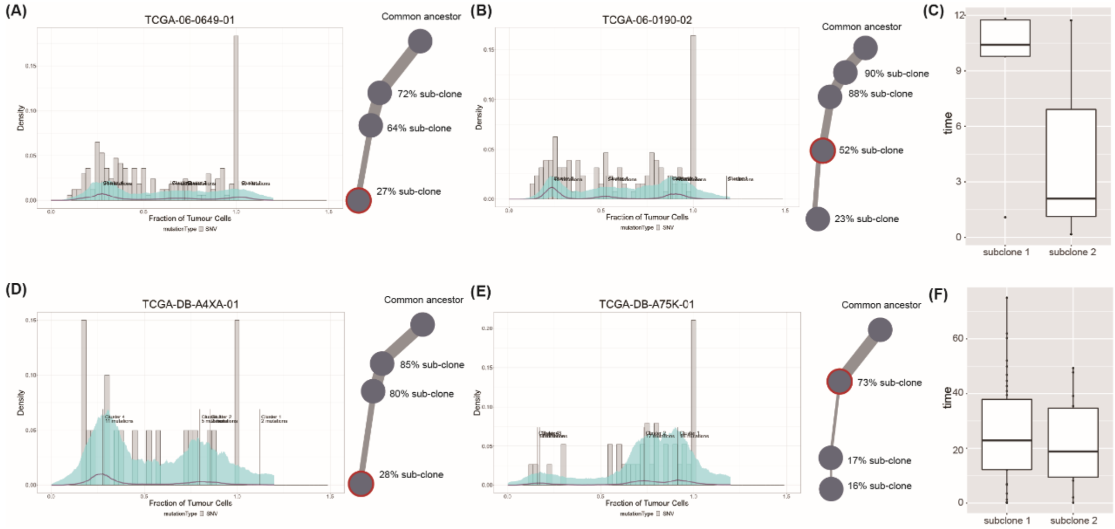

Supplementary Figure 4. Evolutionary trees for patients with risk sub-clonal mutation. (A-C) Evolutionary trees for two patients with sub-clonal mutation of AHNAK2 (A-B)

and survival comparison between subclones in GBM (C). (D-F) Evolutionary trees for two patients with sub-clonal mutation of CIC (D-E) and survival comparison between subclones in LGG (F).

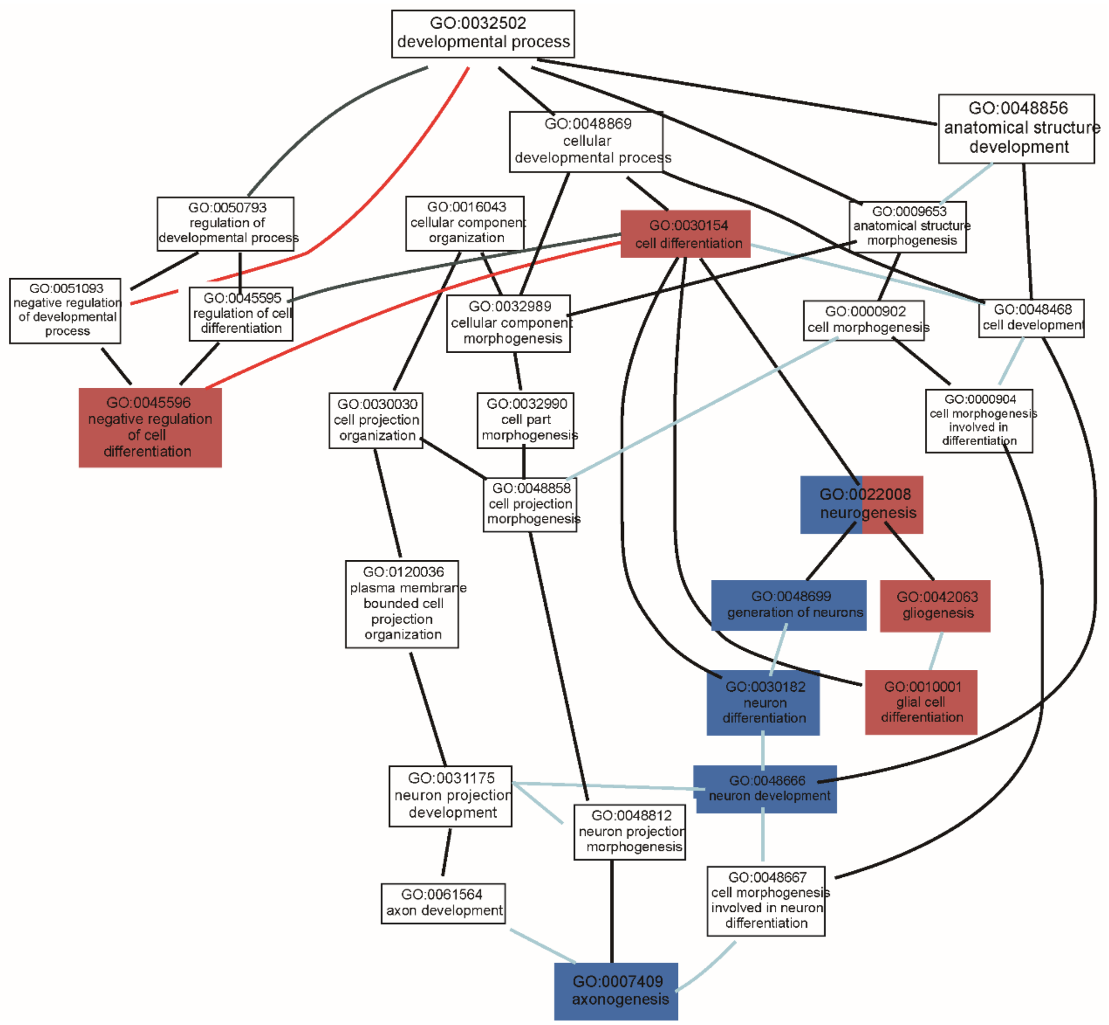

Supplementary Figure 5. GO graph displays the biological process involved in the clonal mutation (red) and the sub-clonal mutation (blue).
