# Supplementary material for: Dissecting and analyzing the Subclonal Mutations Associated with Poor Prognosis in Diffuse Glioma
Source: Biomed Res Int. 2022 Apr 18;2022:4919111. doi: 10.1155/2022/4919111 (PMC9039777; doi:10.1155/2022/4919111)
Supplement: Supplementary 2 — Supplementary Table 1: the clonal status of somatic mutations in GBM and LGG. [file 4919111.f2.docx]

**GBM**

| patient | TP53 | IDH1 | EPPK1 | CD163L1 | DNAH5 | AHNAK | AHNAK2 |
| --- | --- | --- | --- | --- | --- | --- | --- |
| TCGA-02-0003-01 | Clonal | WT | WT | WT | WT | WT | WT |
| TCGA-02-0033-01 | Clonal | WT | WT | WT | WT | WT | WT |
| TCGA-02-0047-01 | WT | WT | WT | WT | WT | WT | WT |
| TCGA-02-0055-01 | Clonal | WT | WT | WT | WT | WT | WT |
| TCGA-02-2466-01 | WT | WT | WT | WT | WT | WT | WT |
| TCGA-02-2470-01 | WT | WT | WT | WT | WT | WT | WT |
| TCGA-02-2483-01 | Clonal | Clonal | WT | WT | WT | WT | WT |
| TCGA-02-2485-01 | Clonal | WT | WT | WT | WT | WT | WT |
| TCGA-02-2486-01 | WT | WT | WT | WT | WT | WT | WT |
| TCGA-06-0119-01 | WT | WT | WT | WT | WT | WT | WT |
| TCGA-06-0122-01 | WT | WT | WT | WT | WT | WT | WT |
| TCGA-06-0124-01 | WT | WT | WT | WT | WT | WT | WT |
| TCGA-06-0125-01 | WT | WT | WT | WT | WT | WT | WT |
| TCGA-06-0125-02 | WT | WT | WT | WT | WT | WT | WT |
| TCGA-06-0126-01 | WT | WT | WT | WT | Clonal | WT | WT |
| TCGA-06-0128-01 | Clonal | Clonal | WT | WT | WT | WT | WT |
| TCGA-06-0129-01 | Clonal | Clonal | WT | WT | WT | WT | WT |
| TCGA-06-0130-01 | Clonal | WT | WT | WT | WT | WT | WT |
| TCGA-06-0132-01 | WT | WT | WT | WT | WT | WT | WT |
| TCGA-06-0137-01 | WT | WT | WT | WT | WT | WT | WT |
| TCGA-06-0140-01 | WT | WT | WT | WT | WT | WT | WT |
| TCGA-06-0141-01 | WT | WT | WT | WT | WT | WT | WT |
| TCGA-06-0145-01 | WT | WT | WT | WT | WT | WT | WT |
| TCGA-06-0151-01 | WT | WT | WT | WT | WT | WT | WT |
| TCGA-06-0152-01 | WT | WT | WT | WT | WT | WT | WT |
| TCGA-06-0152-02 | WT | WT | WT | WT | WT | Subclonal | WT |
| TCGA-06-0154-01 | WT | WT | WT | WT | WT | WT | WT |
| TCGA-06-0155-01 | WT | WT | WT | WT | WT | WT | WT |
| TCGA-06-0157-01 | WT | WT | WT | WT | WT | WT | WT |
| TCGA-06-0158-01 | WT | WT | WT | WT | WT | WT | WT |
| TCGA-06-0166-01 | WT | WT | WT | WT | WT | WT | WT |
| TCGA-06-0168-01 | WT | WT | WT | WT | WT | WT | WT |
| TCGA-06-0169-01 | WT | WT | WT | WT | WT | WT | WT |
| TCGA-06-0171-01 | WT | WT | WT | WT | Subclonal | WT | WT |
| TCGA-06-0171-02 | WT | WT | WT | WT | WT | WT | WT |
| TCGA-06-0173-01 | WT | WT | Clonal | WT | WT | WT | WT |
| TCGA-06-0174-01 | WT | WT | WT | WT | WT | WT | WT |
| TCGA-06-0184-01 | Subclonal | WT | WT | WT | WT | WT | WT |
| TCGA-06-0185-01 | WT | WT | WT | WT | WT | WT | WT |
| TCGA-06-0187-01 | WT | WT | WT | WT | Clonal | WT | WT |
| TCGA-06-0188-01 | Subclonal | WT | WT | WT | WT | WT | WT |
| TCGA-06-0190-01 | Subclonal | WT | WT | WT | Subclonal | WT | WT |
| TCGA-06-0190-02 | Clonal | WT | Clonal | WT | WT | WT | Subclonal |
| TCGA-06-0192-01 | WT | WT | WT | WT | WT | WT | WT |
| TCGA-06-0195-01 | Clonal | WT | WT | WT | WT | WT | WT |
| TCGA-06-0209-01 | WT | WT | WT | WT | WT | WT | WT |
| TCGA-06-0210-01 | Subclonal | WT | WT | WT | WT | WT | WT |
| TCGA-06-0210-02 | Clonal | WT | Clonal | WT | WT | WT | WT |
| TCGA-06-0211-01 | WT | WT | WT | WT | WT | WT | WT |
| TCGA-06-0211-02 | WT | WT | WT | WT | WT | WT | WT |
| TCGA-06-0213-01 | WT | WT | WT | WT | WT | WT | WT |
| TCGA-06-0214-01 | WT | WT | WT | WT | WT | WT | WT |
| TCGA-06-0216-01 | WT | WT | WT | WT | WT | WT | WT |
| TCGA-06-0219-01 | WT | WT | WT | WT | WT | WT | WT |
| TCGA-06-0221-01 | Clonal | Clonal | WT | WT | WT | WT | WT |
| TCGA-06-0221-02 | Clonal | Subclonal | WT | WT | WT | WT | WT |
| TCGA-06-0237-01 | Clonal | WT | WT | WT | WT | WT | WT |
| TCGA-06-0238-01 | Clonal | WT | WT | WT | WT | WT | WT |
| TCGA-06-0241-01 | Subclonal | WT | WT | WT | WT | WT | WT |
| TCGA-06-0644-01 | Clonal | WT | WT | WT | WT | WT | WT |
| TCGA-06-0645-01 | WT | WT | WT | WT | WT | WT | WT |
| TCGA-06-0646-01 | WT | WT | WT | WT | WT | WT | WT |
| TCGA-06-0648-01 | WT | WT | WT | WT | WT | WT | Subclonal |
| TCGA-06-0649-01 | WT | WT | WT | WT | Subclonal | WT | Subclonal |
| TCGA-06-0650-01 | WT | WT | WT | WT | WT | WT | WT |
| TCGA-06-0686-01 | WT | WT | WT | WT | WT | WT | WT |
| TCGA-06-0743-01 | Subclonal | WT | WT | WT | WT | WT | WT |
| TCGA-06-0744-01 | Clonal | WT | Subclonal | WT | WT | WT | Clonal |
| TCGA-06-0745-01 | WT | WT | WT | WT | WT | WT | WT |
| TCGA-06-0747-01 | WT | WT | WT | WT | WT | WT | Clonal |
| TCGA-06-0749-01 | WT | WT | WT | WT | WT | WT | WT |
| TCGA-06-0750-01 | WT | WT | WT | WT | WT | WT | WT |
| TCGA-06-0875-01 | Clonal | WT | WT | WT | WT | WT | WT |
| TCGA-06-0876-01 | Clonal | WT | WT | WT | WT | WT | WT |
| TCGA-06-0877-01 | WT | WT | WT | WT | WT | WT | WT |
| TCGA-06-0878-01 | WT | WT | WT | WT | WT | WT | WT |
| TCGA-06-0879-01 | WT | WT | WT | WT | WT | WT | WT |
| TCGA-06-0882-01 | WT | WT | WT | WT | WT | WT | WT |
| TCGA-06-0939-01 | WT | WT | WT | WT | WT | WT | WT |
| TCGA-06-1084-01 | WT | WT | WT | WT | WT | WT | WT |
| TCGA-06-1087-01 | WT | WT | WT | WT | WT | WT | WT |
| TCGA-06-1800-01 | Clonal | WT | WT | WT | WT | WT | WT |
| TCGA-06-1801-01 | WT | WT | WT | WT | WT | WT | WT |
| TCGA-06-1802-01 | WT | WT | WT | WT | WT | WT | WT |
| TCGA-06-1804-01 | WT | WT | WT | WT | WT | WT | WT |
| TCGA-06-1805-01 | Clonal | Clonal | WT | WT | WT | WT | WT |
| TCGA-06-1806-01 | WT | WT | WT | WT | WT | WT | WT |
| TCGA-06-2557-01 | WT | WT | WT | WT | WT | WT | WT |
| TCGA-06-2558-01 | Clonal | WT | WT | WT | WT | WT | WT |
| TCGA-06-2559-01 | Clonal | WT | WT | WT | WT | WT | WT |
| TCGA-06-2561-01 | WT | WT | WT | WT | WT | WT | WT |
| TCGA-06-2562-01 | WT | WT | WT | WT | WT | WT | WT |
| TCGA-06-2563-01 | Clonal | WT | WT | WT | WT | WT | WT |
| TCGA-06-2564-01 | WT | WT | WT | WT | WT | WT | WT |
| TCGA-06-2565-01 | WT | WT | WT | Clonal | WT | WT | WT |
| TCGA-06-2566-01 | WT | WT | WT | WT | WT | WT | WT |
| TCGA-06-2567-01 | WT | WT | WT | WT | WT | WT | WT |
| TCGA-06-2569-01 | Clonal | WT | WT | WT | WT | WT | WT |
| TCGA-06-2570-01 | Clonal | Clonal | WT | WT | WT | WT | WT |
| TCGA-06-5408-01 | Subclonal | WT | WT | WT | WT | WT | Subclonal |
| TCGA-06-5411-01 | WT | WT | WT | WT | WT | WT | WT |
| TCGA-06-5412-01 | WT | WT | WT | WT | WT | WT | WT |
| TCGA-06-5413-01 | WT | WT | WT | WT | WT | WT | WT |
| TCGA-06-5414-01 | WT | WT | WT | WT | WT | WT | WT |
| TCGA-06-5415-01 | WT | WT | WT | WT | WT | WT | WT |
| TCGA-06-5416-01 | Clonal | WT | Clonal | WT | Clonal | Clonal | WT |
| TCGA-06-5418-01 | WT | WT | WT | WT | WT | WT | WT |
| TCGA-06-5856-01 | WT | WT | WT | WT | WT | Subclonal | WT |
| TCGA-06-5858-01 | Clonal | WT | WT | WT | WT | WT | WT |
| TCGA-06-5859-01 | WT | WT | WT | WT | WT | WT | WT |
| TCGA-06-6388-01 | WT | WT | WT | WT | WT | WT | WT |
| TCGA-06-6389-01 | Clonal | Clonal | WT | WT | WT | WT | WT |
| TCGA-06-6390-01 | WT | WT | Clonal | WT | WT | WT | WT |
| TCGA-06-6391-01 | WT | WT | WT | WT | Subclonal | WT | WT |
| TCGA-06-6693-01 | WT | WT | WT | WT | WT | WT | WT |
| TCGA-06-6694-01 | Clonal | WT | WT | WT | WT | WT | WT |
| TCGA-06-6695-01 | WT | WT | Subclonal | WT | WT | WT | WT |
| TCGA-06-6697-01 | WT | WT | WT | WT | Clonal | WT | WT |
| TCGA-06-6698-01 | Clonal | WT | WT | WT | WT | WT | WT |
| TCGA-06-6699-01 | WT | WT | WT | WT | WT | WT | WT |
| TCGA-06-6700-01 | WT | WT | WT | WT | WT | WT | WT |
| TCGA-06-6701-01 | Clonal | Clonal | WT | WT | WT | WT | WT |
| TCGA-06-A5U0-01 | Clonal | WT | WT | WT | WT | WT | WT |
| TCGA-06-A5U1-01 | WT | WT | WT | WT | WT | WT | WT |
| TCGA-06-A6S0-01 | WT | WT | WT | WT | Clonal | WT | WT |
| TCGA-06-A6S1-01 | WT | WT | WT | WT | WT | WT | WT |
| TCGA-06-A7TK-01 | WT | WT | Subclonal | WT | WT | WT | Clonal |
| TCGA-06-A7TL-01 | Clonal | Subclonal | WT | WT | WT | WT | WT |
| TCGA-08-0386-01 | WT | WT | WT | WT | WT | WT | WT |
| TCGA-12-0615-01 | WT | WT | WT | WT | WT | WT | WT |
| TCGA-12-0616-01 | WT | WT | WT | WT | WT | WT | WT |
| TCGA-12-0618-01 | Clonal | WT | WT | WT | WT | WT | WT |
| TCGA-12-0619-01 | Clonal | WT | WT | WT | Clonal | WT | WT |
| TCGA-12-0656-01 | WT | WT | WT | WT | WT | WT | WT |
| TCGA-12-0657-01 | WT | WT | WT | WT | WT | WT | WT |
| TCGA-12-0662-01 | Subclonal | WT | WT | WT | WT | WT | WT |
| TCGA-12-0670-01 | WT | WT | WT | WT | WT | WT | WT |
| TCGA-12-0688-01 | WT | WT | WT | WT | WT | WT | WT |
| TCGA-12-0691-01 | Clonal | WT | WT | WT | WT | WT | WT |
| TCGA-12-0692-01 | WT | WT | WT | WT | WT | WT | WT |
| TCGA-12-0707-01 | Clonal | WT | WT | WT | WT | WT | WT |
| TCGA-12-0773-01 | Clonal | WT | WT | WT | WT | WT | WT |
| TCGA-12-0775-01 | WT | WT | WT | Clonal | Clonal | WT | WT |
| TCGA-12-0778-01 | WT | WT | WT | Clonal | WT | WT | WT |
| TCGA-12-0818-01 | Clonal | Clonal | WT | WT | WT | WT | WT |
| TCGA-12-0819-01 | WT | WT | WT | WT | WT | WT | WT |
| TCGA-12-0820-01 | WT | WT | WT | WT | WT | WT | WT |
| TCGA-12-0821-01 | WT | WT | WT | WT | WT | WT | WT |
| TCGA-12-0822-01 | WT | WT | WT | WT | WT | WT | WT |
| TCGA-12-0826-01 | WT | WT | WT | WT | WT | WT | WT |
| TCGA-12-0827-01 | WT | Clonal | WT | WT | WT | WT | WT |
| TCGA-12-0828-01 | WT | WT | WT | WT | WT | WT | WT |
| TCGA-12-0829-01 | WT | WT | WT | WT | Clonal | Subclonal | WT |
| TCGA-12-1088-01 | Clonal | WT | WT | WT | WT | WT | WT |
| TCGA-12-1089-01 | WT | WT | WT | WT | WT | WT | WT |
| TCGA-12-1092-01 | Clonal | WT | WT | WT | Subclonal | WT | WT |
| TCGA-12-1093-01 | WT | WT | WT | WT | WT | Subclonal | WT |
| TCGA-12-1598-01 | Subclonal | WT | WT | WT | WT | WT | WT |
| TCGA-12-1599-01 | WT | WT | WT | WT | WT | WT | WT |
| TCGA-12-1600-01 | Clonal | WT | WT | WT | WT | WT | WT |
| TCGA-12-1602-01 | WT | WT | WT | WT | WT | WT | WT |
| TCGA-12-3644-01 | Clonal | WT | WT | WT | WT | WT | WT |
| TCGA-12-3646-01 | WT | WT | WT | WT | WT | WT | WT |
| TCGA-12-3648-01 | WT | WT | WT | WT | WT | WT | WT |
| TCGA-12-3649-01 | WT | WT | WT | WT | WT | WT | WT |
| TCGA-12-3650-01 | WT | WT | WT | WT | WT | WT | WT |
| TCGA-12-3651-01 | WT | WT | WT | WT | WT | WT | WT |
| TCGA-12-3652-01 | WT | WT | WT | WT | WT | WT | WT |
| TCGA-12-3653-01 | WT | WT | WT | WT | WT | WT | WT |
| TCGA-12-5295-01 | WT | WT | WT | WT | WT | WT | WT |
| TCGA-12-5299-01 | WT | WT | Clonal | WT | WT | WT | WT |
| TCGA-12-5301-01 | WT | WT | WT | WT | WT | WT | WT |
| TCGA-14-0736-01 | WT | WT | WT | WT | WT | WT | WT |
| TCGA-14-0736-02 | WT | WT | WT | WT | WT | WT | WT |
| TCGA-14-0740-01 | Clonal | WT | WT | WT | WT | WT | WT |
| TCGA-14-0781-01 | WT | WT | WT | WT | WT | WT | WT |
| TCGA-14-0786-01 | WT | WT | WT | WT | WT | WT | WT |
| TCGA-14-0787-01 | WT | WT | WT | WT | WT | WT | WT |
| TCGA-14-0789-01 | WT | WT | WT | WT | WT | WT | WT |
| TCGA-14-0790-01 | WT | WT | WT | WT | WT | WT | Clonal |
| TCGA-14-0812-01 | Clonal | WT | WT | WT | WT | WT | WT |
| TCGA-14-0813-01 | WT | WT | WT | WT | WT | Subclonal | WT |
| TCGA-14-0817-01 | Clonal | WT | WT | WT | WT | WT | WT |
| TCGA-14-0862-01 | WT | WT | WT | WT | WT | WT | WT |
| TCGA-14-0865-01 | WT | WT | WT | WT | WT | WT | WT |
| TCGA-14-0866-01 | WT | WT | WT | WT | WT | WT | WT |
| TCGA-14-0867-01 | Clonal | WT | WT | WT | WT | WT | WT |
| TCGA-14-0871-01 | Clonal | WT | WT | WT | WT | WT | WT |
| TCGA-14-1034-01 | Clonal | WT | WT | WT | WT | WT | Clonal |
| TCGA-14-1034-02 | Clonal | WT | WT | WT | WT | WT | Clonal |
| TCGA-14-1037-01 | WT | WT | WT | WT | WT | WT | WT |
| TCGA-14-1043-01 | WT | WT | WT | WT | WT | WT | WT |
| TCGA-14-1395-01 | WT | WT | WT | WT | WT | WT | WT |
| TCGA-14-1396-01 | Clonal | WT | WT | WT | WT | Subclonal | WT |
| TCGA-14-1450-01 | WT | WT | WT | WT | WT | WT | Clonal |
| TCGA-14-1451-01 | WT | WT | WT | WT | WT | WT | WT |
| TCGA-14-1453-01 | WT | WT | WT | WT | WT | WT | WT |
| TCGA-14-1455-01 | WT | WT | WT | WT | WT | WT | WT |
| TCGA-14-1456-01 | Clonal | Subclonal | WT | WT | WT | WT | WT |
| TCGA-14-1458-01 | Clonal | Clonal | WT | WT | WT | WT | WT |
| TCGA-14-1794-01 | Clonal | WT | WT | WT | WT | WT | WT |
| TCGA-14-1795-01 | Clonal | WT | WT | WT | WT | WT | WT |
| TCGA-14-1821-01 | Clonal | Clonal | WT | WT | WT | WT | WT |
| TCGA-14-1823-01 | WT | WT | WT | WT | WT | WT | WT |
| TCGA-14-1825-01 | Clonal | WT | WT | WT | WT | WT | WT |
| TCGA-14-1827-01 | WT | WT | WT | WT | WT | WT | WT |
| TCGA-14-1829-01 | WT | WT | WT | WT | WT | WT | WT |
| TCGA-14-2554-01 | WT | WT | WT | WT | WT | WT | WT |
| TCGA-14-3477-01 | Clonal | WT | WT | WT | WT | WT | WT |
| TCGA-14-4157-01 | Clonal | Subclonal | WT | WT | WT | WT | WT |
| TCGA-15-0742-01 | WT | WT | WT | WT | WT | WT | WT |
| TCGA-15-1446-01 | WT | WT | WT | WT | WT | Clonal | WT |
| TCGA-16-0846-01 | Clonal | WT | WT | WT | WT | WT | WT |
| TCGA-16-0848-01 | Clonal | WT | WT | WT | WT | WT | WT |
| TCGA-16-0849-01 | Clonal | Clonal | WT | WT | WT | WT | WT |
| TCGA-16-0850-01 | Clonal | Clonal | WT | WT | WT | WT | WT |
| TCGA-16-0861-01 | WT | WT | WT | WT | Clonal | WT | WT |
| TCGA-16-1045-01 | WT | WT | WT | WT | WT | WT | WT |
| TCGA-16-1460-01 | Clonal | Clonal | WT | WT | WT | WT | WT |
| TCGA-19-0957-01 | WT | WT | WT | WT | WT | WT | WT |
| TCGA-19-1385-01 | WT | WT | WT | WT | WT | WT | WT |
| TCGA-19-1386-01 | WT | WT | WT | Subclonal | WT | Subclonal | WT |
| TCGA-19-1387-01 | WT | WT | WT | WT | WT | WT | WT |
| TCGA-19-1388-01 | Clonal | WT | WT | WT | WT | WT | WT |
| TCGA-19-1389-01 | WT | WT | WT | WT | WT | WT | WT |
| TCGA-19-1390-01 | WT | WT | WT | WT | WT | WT | WT |
| TCGA-19-1786-01 | WT | WT | WT | Clonal | WT | WT | WT |
| TCGA-19-1787-01 | Clonal | WT | WT | WT | WT | Subclonal | WT |
| TCGA-19-1789-01 | WT | WT | WT | WT | WT | WT | WT |
| TCGA-19-1791-01 | WT | WT | WT | WT | WT | WT | WT |
| TCGA-19-2619-01 | WT | WT | WT | WT | WT | WT | WT |
| TCGA-19-2620-01 | WT | WT | WT | WT | WT | WT | WT |
| TCGA-19-2621-01 | WT | WT | WT | WT | WT | Subclonal | Subclonal |
| TCGA-19-2623-01 | Clonal | WT | WT | WT | WT | WT | WT |
| TCGA-19-2624-01 | WT | WT | WT | WT | WT | WT | Subclonal |
| TCGA-19-2625-01 | Clonal | WT | WT | WT | WT | WT | WT |
| TCGA-19-2629-01 | Clonal | Clonal | WT | WT | WT | WT | WT |
| TCGA-19-2631-01 | WT | WT | WT | WT | WT | WT | WT |
| TCGA-19-4065-01 | Clonal | WT | WT | WT | WT | WT | WT |
| TCGA-19-4065-02 | Clonal | WT | WT | WT | WT | WT | WT |
| TCGA-19-5947-01 | WT | WT | WT | WT | WT | WT | WT |
| TCGA-19-5950-01 | WT | WT | WT | WT | Clonal | WT | WT |
| TCGA-19-5951-01 | WT | WT | WT | WT | Clonal | WT | WT |
| TCGA-19-5952-01 | WT | WT | WT | WT | WT | WT | WT |
| TCGA-19-5953-01 | WT | WT | WT | WT | WT | WT | WT |
| TCGA-19-5954-01 | WT | WT | WT | WT | WT | WT | WT |
| TCGA-19-5955-01 | WT | WT | WT | WT | WT | WT | WT |
| TCGA-19-5956-01 | Clonal | WT | WT | Clonal | WT | Clonal | WT |
| TCGA-19-5958-01 | WT | WT | WT | WT | WT | WT | WT |
| TCGA-19-5959-01 | WT | WT | WT | WT | WT | WT | WT |
| TCGA-19-5960-01 | WT | WT | WT | WT | WT | WT | WT |
| TCGA-19-A60I-01 | WT | WT | WT | WT | WT | WT | WT |
| TCGA-19-A6J4-01 | WT | WT | WT | WT | WT | WT | WT |
| TCGA-19-A6J5-01 | Clonal | Clonal | WT | WT | WT | WT | WT |
| TCGA-26-1439-01 | WT | WT | WT | WT | WT | WT | WT |
| TCGA-26-1442-01 | Clonal | Clonal | WT | WT | WT | WT | WT |
| TCGA-26-1799-01 | WT | WT | WT | WT | WT | Subclonal | WT |
| TCGA-26-5132-01 | WT | WT | WT | WT | WT | WT | WT |
| TCGA-26-5133-01 | Clonal | WT | WT | WT | WT | WT | WT |
| TCGA-26-5134-01 | WT | WT | WT | Subclonal | WT | WT | WT |
| TCGA-26-5135-01 | WT | WT | WT | WT | WT | WT | WT |
| TCGA-26-5136-01 | Clonal | WT | WT | WT | WT | WT | WT |
| TCGA-26-5139-01 | WT | WT | Subclonal | WT | WT | WT | WT |
| TCGA-26-6173-01 | WT | WT | WT | WT | WT | WT | WT |
| TCGA-26-6174-01 | WT | WT | WT | WT | WT | WT | WT |
| TCGA-26-A7UX-01 | WT | WT | WT | WT | WT | WT | WT |
| TCGA-27-1830-01 | Clonal | WT | WT | Subclonal | Clonal | WT | WT |
| TCGA-27-1831-01 | WT | WT | WT | WT | WT | WT | WT |
| TCGA-27-1832-01 | WT | WT | WT | WT | WT | WT | WT |
| TCGA-27-1833-01 | WT | WT | WT | WT | WT | WT | WT |
| TCGA-27-1834-01 | WT | WT | WT | WT | WT | WT | WT |
| TCGA-27-1835-01 | Clonal | WT | WT | WT | WT | WT | WT |
| TCGA-27-1836-01 | Subclonal | WT | WT | WT | WT | WT | WT |
| TCGA-27-1837-01 | WT | WT | WT | WT | WT | WT | WT |
| TCGA-27-1838-01 | Clonal | WT | WT | WT | WT | WT | WT |
| TCGA-27-2518-01 | WT | WT | WT | WT | WT | WT | WT |
| TCGA-27-2519-01 | Clonal | WT | WT | WT | WT | WT | WT |
| TCGA-27-2521-01 | Clonal | Subclonal | WT | WT | WT | WT | WT |
| TCGA-27-2523-01 | WT | WT | WT | WT | WT | WT | WT |
| TCGA-27-2524-01 | WT | WT | WT | WT | WT | WT | WT |
| TCGA-27-2526-01 | WT | WT | WT | WT | Subclonal | WT | WT |
| TCGA-27-2527-01 | WT | WT | WT | WT | WT | WT | WT |
| TCGA-27-2528-01 | WT | WT | WT | WT | WT | WT | WT |
| TCGA-28-1746-01 | Clonal | WT | WT | WT | WT | WT | WT |
| TCGA-28-1747-01 | WT | WT | WT | Subclonal | WT | WT | WT |
| TCGA-28-1749-01 | WT | WT | WT | WT | WT | WT | WT |
| TCGA-28-1750-01 | WT | WT | WT | WT | WT | WT | WT |
| TCGA-28-1751-01 | WT | WT | WT | WT | WT | Clonal | WT |
| TCGA-28-1752-01 | Subclonal | WT | WT | WT | WT | WT | WT |
| TCGA-28-1753-01 | Subclonal | WT | WT | WT | WT | WT | WT |
| TCGA-28-1755-01 | WT | WT | WT | WT | WT | WT | WT |
| TCGA-28-1757-01 | WT | WT | WT | WT | WT | WT | WT |
| TCGA-28-2502-01 | WT | WT | WT | WT | WT | WT | WT |
| TCGA-28-2506-01 | WT | WT | WT | WT | WT | WT | WT |
| TCGA-28-2509-01 | Clonal | WT | WT | WT | WT | WT | WT |
| TCGA-28-2513-01 | WT | WT | WT | WT | WT | WT | WT |
| TCGA-28-2514-01 | WT | WT | WT | WT | WT | WT | WT |
| TCGA-28-5204-01 | WT | WT | WT | WT | WT | WT | WT |
| TCGA-28-5207-01 | Clonal | WT | WT | WT | WT | WT | WT |
| TCGA-28-5208-01 | WT | WT | WT | WT | WT | Subclonal | WT |
| TCGA-28-5209-01 | WT | WT | Subclonal | WT | WT | WT | Clonal |
| TCGA-28-5211-01 | WT | WT | WT | WT | WT | WT | Subclonal |
| TCGA-28-5213-01 | WT | WT | WT | WT | WT | Clonal | WT |
| TCGA-28-5214-01 | WT | WT | WT | WT | WT | WT | WT |
| TCGA-28-5215-01 | WT | WT | WT | WT | WT | WT | WT |
| TCGA-28-5216-01 | Clonal | WT | WT | WT | WT | WT | WT |
| TCGA-28-5218-01 | WT | WT | WT | WT | WT | WT | WT |
| TCGA-28-5219-01 | Clonal | WT | WT | WT | WT | WT | WT |
| TCGA-28-5220-01 | WT | WT | WT | WT | WT | WT | WT |
| TCGA-28-6450-01 | WT | WT | WT | WT | WT | WT | WT |
| TCGA-32-1970-01 | Clonal | WT | WT | WT | WT | WT | WT |
| TCGA-32-1976-01 | WT | WT | WT | WT | WT | WT | WT |
| TCGA-32-1977-01 | WT | WT | WT | WT | WT | WT | WT |
| TCGA-32-1979-01 | WT | WT | WT | WT | WT | WT | WT |
| TCGA-32-1980-01 | WT | WT | WT | WT | WT | WT | WT |
| TCGA-32-1982-01 | WT | WT | WT | WT | WT | WT | WT |
| TCGA-32-1986-01 | WT | WT | WT | WT | WT | WT | WT |
| TCGA-32-1991-01 | WT | WT | WT | WT | WT | WT | WT |
| TCGA-32-2491-01 | Clonal | WT | WT | WT | WT | WT | WT |
| TCGA-32-2494-01 | WT | WT | WT | WT | WT | WT | WT |
| TCGA-32-2495-01 | WT | WT | WT | WT | WT | WT | WT |
| TCGA-32-2615-01 | WT | WT | WT | WT | WT | WT | WT |
| TCGA-32-2616-01 | Subclonal | WT | Clonal | WT | Subclonal | WT | WT |
| TCGA-32-2632-01 | WT | WT | WT | WT | WT | WT | WT |
| TCGA-32-2634-01 | Clonal | WT | WT | WT | Clonal | WT | WT |
| TCGA-32-2638-01 | WT | WT | WT | WT | WT | WT | WT |
| TCGA-32-4208-01 | Clonal | Subclonal | WT | WT | WT | WT | WT |
| TCGA-32-4210-01 | Clonal | WT | WT | WT | WT | Clonal | WT |
| TCGA-32-4211-01 | WT | WT | WT | WT | Subclonal | WT | WT |
| TCGA-32-4213-01 | WT | WT | WT | WT | Clonal | WT | Clonal |
| TCGA-32-4719-01 | WT | WT | WT | WT | WT | WT | WT |
| TCGA-32-5222-01 | WT | WT | WT | WT | Subclonal | WT | Clonal |
| TCGA-41-2571-01 | WT | WT | WT | WT | WT | WT | WT |
| TCGA-41-2572-01 | WT | WT | WT | WT | WT | WT | WT |
| TCGA-41-2573-01 | WT | WT | WT | WT | WT | WT | WT |
| TCGA-41-2575-01 | Clonal | WT | WT | WT | WT | WT | WT |
| TCGA-41-3392-01 | WT | WT | WT | WT | Subclonal | WT | WT |
| TCGA-41-3393-01 | WT | WT | WT | WT | WT | WT | WT |
| TCGA-41-3915-01 | WT | WT | WT | WT | WT | WT | Subclonal |
| TCGA-41-4097-01 | WT | WT | WT | WT | WT | WT | WT |
| TCGA-41-5651-01 | Clonal | WT | WT | WT | WT | WT | Clonal |
| TCGA-41-6646-01 | WT | WT | WT | WT | WT | WT | WT |
| TCGA-4W-AA9R-01 | Clonal | WT | WT | WT | WT | WT | WT |
| TCGA-4W-AA9S-01 | WT | WT | Subclonal | WT | WT | WT | WT |
| TCGA-4W-AA9T-01 | WT | WT | WT | WT | WT | WT | Clonal |
| TCGA-74-6573-01 | Clonal | WT | WT | WT | WT | WT | WT |
| TCGA-74-6575-01 | WT | WT | WT | WT | WT | WT | WT |
| TCGA-74-6577-01 | WT | WT | WT | WT | WT | WT | WT |
| TCGA-74-6578-01 | WT | WT | WT | WT | WT | WT | WT |
| TCGA-74-6581-01 | WT | WT | WT | WT | WT | WT | WT |
| TCGA-74-6584-01 | WT | WT | WT | WT | WT | WT | WT |
| TCGA-76-4925-01 | Subclonal | WT | WT | WT | WT | WT | WT |
| TCGA-76-4926-01 | WT | WT | WT | WT | WT | WT | WT |
| TCGA-76-4928-01 | WT | WT | WT | WT | WT | WT | WT |
| TCGA-76-4929-01 | Clonal | WT | WT | WT | WT | WT | WT |
| TCGA-76-4931-01 | WT | WT | WT | WT | WT | WT | Clonal |
| TCGA-76-4934-01 | Clonal | WT | WT | WT | WT | WT | WT |
| TCGA-76-4935-01 | WT | WT | WT | WT | WT | WT | WT |
| TCGA-76-6191-01 | WT | WT | WT | WT | WT | WT | WT |
| TCGA-76-6192-01 | WT | WT | WT | WT | WT | WT | WT |
| TCGA-76-6193-01 | Clonal | WT | WT | WT | WT | WT | WT |
| TCGA-76-6280-01 | WT | WT | WT | WT | WT | WT | WT |
| TCGA-76-6282-01 | WT | WT | WT | WT | WT | WT | WT |
| TCGA-76-6283-01 | Clonal | WT | WT | Clonal | WT | WT | WT |
| TCGA-76-6285-01 | WT | WT | WT | WT | WT | WT | WT |
| TCGA-76-6286-01 | WT | WT | WT | WT | WT | WT | WT |
| TCGA-76-6656-01 | WT | WT | WT | WT | WT | WT | WT |
| TCGA-76-6657-01 | WT | WT | Clonal | WT | WT | WT | WT |
| TCGA-76-6660-01 | Clonal | WT | WT | WT | WT | WT | WT |
| TCGA-76-6661-01 | WT | WT | WT | WT | WT | WT | WT |
| TCGA-76-6662-01 | Clonal | WT | WT | WT | WT | WT | WT |
| TCGA-76-6663-01 | Subclonal | WT | WT | WT | WT | WT | WT |
| TCGA-76-6664-01 | Clonal | WT | WT | WT | WT | WT | WT |
| TCGA-81-5910-01 | WT | WT | WT | WT | WT | WT | WT |
| TCGA-81-5911-01 | WT | WT | WT | WT | WT | WT | WT |
| TCGA-87-5896-01 | WT | WT | WT | WT | WT | WT | WT |
| TCGA-OX-A56R-01 | WT | WT | WT | WT | WT | WT | WT |
| TCGA-RR-A6KA-01 | WT | WT | WT | Subclonal | WT | WT | WT |
| TCGA-RR-A6KB-01 | WT | WT | WT | WT | WT | WT | Clonal |
| TCGA-RR-A6KC-01 | WT | WT | WT | WT | WT | WT | WT |

**LGG**

| patient | IDH1 | MUC17 | EGFR | CIC | PTEN | RYR2 | NF1 | FLG |
| --- | --- | --- | --- | --- | --- | --- | --- | --- |
| TCGA-CS-4938-01 | Clonal | WT | WT | WT | WT | WT | WT | WT |
| TCGA-CS-4941-01 | WT | WT | WT | WT | WT | WT | WT | WT |
| TCGA-CS-4942-01 | Clonal | WT | WT | WT | WT | WT | WT | WT |
| TCGA-CS-4943-01 | Clonal | WT | WT | WT | WT | WT | WT | WT |
| TCGA-CS-4944-01 | Clonal | WT | WT | WT | WT | WT | WT | WT |
| TCGA-CS-5390-01 | Subclonal | WT | WT | WT | WT | WT | WT | WT |
| TCGA-CS-5393-01 | Clonal | WT | WT | WT | WT | WT | WT | WT |
| TCGA-CS-5394-01 | Subclonal | WT | WT | Subclonal | WT | WT | WT | WT |
| TCGA-CS-5395-01 | WT | WT | WT | WT | Subclonal | WT | WT | WT |
| TCGA-CS-5396-01 | Clonal | WT | WT | WT | WT | WT | WT | WT |
| TCGA-CS-5397-01 | WT | WT | WT | WT | WT | WT | Clonal | WT |
| TCGA-CS-6186-01 | WT | WT | WT | WT | WT | WT | WT | WT |
| TCGA-CS-6188-01 | WT | WT | WT | WT | WT | WT | WT | WT |
| TCGA-CS-6290-01 | Clonal | WT | WT | WT | WT | WT | WT | WT |
| TCGA-CS-6665-01 | Clonal | WT | WT | WT | WT | Clonal | WT | WT |
| TCGA-CS-6666-01 | Clonal | WT | WT | WT | WT | WT | WT | WT |
| TCGA-CS-6667-01 | Clonal | WT | WT | WT | WT | WT | WT | WT |
| TCGA-CS-6668-01 | Clonal | WT | WT | Subclonal | WT | WT | WT | WT |
| TCGA-CS-6670-01 | Clonal | WT | WT | Subclonal | WT | WT | WT | WT |
| TCGA-DB-5270-01 | Clonal | WT | WT | WT | WT | WT | WT | WT |
| TCGA-DB-5273-01 | Subclonal | WT | WT | WT | WT | WT | WT | WT |
| TCGA-DB-5274-01 | Subclonal | WT | WT | Subclonal | WT | WT | WT | WT |
| TCGA-DB-5275-01 | Clonal | WT | WT | WT | WT | WT | WT | WT |
| TCGA-DB-5276-01 | Clonal | WT | WT | WT | WT | WT | WT | WT |
| TCGA-DB-5277-01 | Subclonal | WT | WT | WT | WT | WT | WT | WT |
| TCGA-DB-5278-01 | Clonal | WT | WT | Subclonal | WT | WT | WT | WT |
| TCGA-DB-5279-01 | Clonal | WT | WT | WT | WT | WT | WT | WT |
| TCGA-DB-5280-01 | Clonal | WT | WT | WT | WT | WT | WT | WT |
| TCGA-DB-5281-01 | Clonal | WT | WT | WT | WT | WT | WT | WT |
| TCGA-DB-A4X9-01 | Clonal | WT | WT | WT | WT | WT | WT | WT |
| TCGA-DB-A4XA-01 | Clonal | WT | WT | Subclonal | WT | WT | WT | WT |
| TCGA-DB-A4XB-01 | Subclonal | WT | WT | WT | WT | WT | WT | WT |
| TCGA-DB-A4XC-01 | Subclonal | WT | WT | WT | WT | WT | WT | WT |
| TCGA-DB-A4XD-01 | Clonal | WT | WT | WT | WT | WT | WT | WT |
| TCGA-DB-A4XE-01 | Clonal | WT | WT | WT | WT | WT | WT | WT |
| TCGA-DB-A4XF-01 | Clonal | WT | WT | WT | WT | WT | WT | WT |
| TCGA-DB-A4XG-01 | WT | WT | WT | WT | WT | WT | Clonal | WT |
| TCGA-DB-A4XH-01 | Clonal | WT | WT | WT | WT | WT | WT | WT |
| TCGA-DB-A64L-01 | Clonal | Subclonal | WT | Clonal | WT | WT | WT | WT |
| TCGA-DB-A64O-01 | WT | WT | WT | WT | WT | WT | Clonal | WT |
| TCGA-DB-A64P-01 | Clonal | WT | WT | Subclonal | WT | WT | WT | WT |
| TCGA-DB-A64Q-01 | Clonal | WT | WT | Clonal | WT | WT | WT | WT |
| TCGA-DB-A64R-01 | Clonal | WT | WT | Clonal | WT | WT | WT | WT |
| TCGA-DB-A64S-01 | Clonal | WT | WT | WT | WT | WT | WT | WT |
| TCGA-DB-A64U-01 | Subclonal | WT | WT | Subclonal | WT | WT | WT | WT |
| TCGA-DB-A64V-01 | Clonal | WT | WT | Subclonal | WT | WT | WT | WT |
| TCGA-DB-A64W-01 | Clonal | WT | WT | Clonal | WT | WT | WT | WT |
| TCGA-DB-A64X-01 | Clonal | WT | WT | WT | WT | WT | WT | WT |
| TCGA-DB-A75K-01 | Clonal | WT | WT | Subclonal | WT | WT | Subclonal | WT |
| TCGA-DB-A75L-01 | Clonal | WT | WT | WT | WT | WT | WT | WT |
| TCGA-DB-A75M-01 | Subclonal | WT | WT | WT | WT | WT | WT | WT |
| TCGA-DB-A75O-01 | Clonal | WT | WT | WT | WT | WT | WT | WT |
| TCGA-DH-5140-01 | WT | WT | WT | WT | WT | WT | Clonal | WT |
| TCGA-DH-5141-01 | WT | WT | WT | Clonal | WT | WT | WT | WT |
| TCGA-DH-5142-01 | Clonal | WT | WT | WT | WT | WT | WT | WT |
| TCGA-DH-5143-01 | Clonal | WT | WT | WT | WT | WT | WT | WT |
| TCGA-DH-5144-01 | Clonal | WT | WT | Subclonal | WT | WT | WT | WT |
| TCGA-DH-A669-01 | Clonal | WT | WT | WT | WT | WT | WT | WT |
| TCGA-DH-A669-02 | Clonal | WT | WT | WT | WT | WT | WT | WT |
| TCGA-DH-A66B-01 | Clonal | WT | WT | WT | WT | WT | WT | WT |
| TCGA-DH-A66D-01 | Clonal | WT | WT | WT | WT | WT | WT | WT |
| TCGA-DH-A66F-01 | Clonal | WT | WT | Subclonal | WT | WT | WT | WT |
| TCGA-DH-A66G-01 | Clonal | WT | WT | WT | WT | WT | WT | WT |
| TCGA-DH-A7UR-01 | WT | WT | WT | Clonal | WT | WT | WT | WT |
| TCGA-DH-A7US-01 | Clonal | WT | WT | Subclonal | WT | WT | WT | WT |
| TCGA-DH-A7UT-01 | Clonal | WT | WT | WT | WT | WT | WT | WT |
| TCGA-DH-A7UU-01 | Clonal | WT | WT | WT | Clonal | WT | WT | WT |
| TCGA-DH-A7UV-01 | Clonal | WT | WT | WT | Clonal | WT | WT | WT |
| TCGA-DU-5847-01 | WT | WT | WT | WT | Subclonal | WT | Clonal | Subclonal |
| TCGA-DU-5849-01 | WT | WT | WT | WT | WT | WT | WT | WT |
| TCGA-DU-5851-01 | Clonal | WT | WT | WT | WT | WT | WT | WT |
| TCGA-DU-5853-01 | Clonal | WT | WT | WT | WT | WT | WT | WT |
| TCGA-DU-5854-01 | WT | WT | WT | WT | WT | WT | WT | WT |
| TCGA-DU-5855-01 | Clonal | WT | WT | WT | WT | WT | WT | WT |
| TCGA-DU-5870-02 | Clonal | WT | WT | Subclonal | WT | WT | WT | WT |
| TCGA-DU-5871-01 | Clonal | WT | WT | WT | WT | WT | WT | WT |
| TCGA-DU-5872-01 | Clonal | WT | WT | WT | WT | WT | WT | WT |
| TCGA-DU-5872-02 | WT | WT | WT | WT | WT | WT | WT | WT |
| TCGA-DU-5874-01 | Subclonal | WT | WT | WT | WT | WT | WT | WT |
| TCGA-DU-6392-01 | WT | WT | Subclonal | WT | Clonal | WT | WT | WT |
| TCGA-DU-6393-01 | Subclonal | WT | WT | Subclonal | WT | WT | WT | WT |
| TCGA-DU-6394-01 | Subclonal | WT | WT | WT | WT | WT | WT | WT |
| TCGA-DU-6395-01 | Clonal | WT | WT | WT | WT | WT | WT | WT |
| TCGA-DU-6396-01 | Clonal | Clonal | WT | WT | WT | Clonal | WT | WT |
| TCGA-DU-6397-01 | Clonal | WT | WT | WT | WT | WT | Subclonal | Subclonal |
| TCGA-DU-6397-02 | Clonal | WT | WT | Subclonal | WT | WT | WT | WT |
| TCGA-DU-6399-01 | Clonal | WT | WT | WT | WT | WT | WT | WT |
| TCGA-DU-6400-01 | Clonal | WT | WT | Subclonal | WT | WT | WT | WT |
| TCGA-DU-6401-01 | Clonal | WT | WT | WT | WT | WT | WT | WT |
| TCGA-DU-6402-01 | WT | WT | WT | WT | Subclonal | Clonal | Clonal | WT |
| TCGA-DU-6403-01 | WT | WT | WT | WT | WT | WT | WT | Clonal |
| TCGA-DU-6405-01 | WT | WT | WT | WT | WT | WT | WT | WT |
| TCGA-DU-6407-01 | Clonal | WT | WT | WT | WT | WT | WT | WT |
| TCGA-DU-6408-01 | Clonal | WT | WT | WT | WT | WT | WT | WT |
| TCGA-DU-6410-01 | Clonal | WT | WT | WT | WT | WT | WT | WT |
| TCGA-DU-6542-01 | Subclonal | WT | WT | WT | WT | WT | WT | WT |
| TCGA-DU-7006-01 | WT | WT | Subclonal | WT | WT | WT | Subclonal | WT |
| TCGA-DU-7007-01 | Clonal | Subclonal | WT | WT | WT | WT | WT | WT |
| TCGA-DU-7008-01 | Clonal | WT | WT | WT | WT | WT | WT | WT |
| TCGA-DU-7009-01 | Clonal | WT | WT | WT | WT | WT | WT | WT |
| TCGA-DU-7010-01 | Clonal | WT | WT | WT | WT | WT | WT | Subclonal |
| TCGA-DU-7011-01 | Clonal | WT | WT | WT | WT | WT | WT | WT |
| TCGA-DU-7012-01 | WT | Clonal | Clonal | WT | Clonal | WT | WT | WT |
| TCGA-DU-7013-01 | WT | WT | WT | WT | WT | WT | WT | WT |
| TCGA-DU-7015-01 | Clonal | WT | WT | WT | WT | WT | WT | WT |
| TCGA-DU-7018-01 | Clonal | WT | WT | Clonal | WT | WT | WT | WT |
| TCGA-DU-7019-01 | Clonal | WT | WT | WT | WT | WT | WT | WT |
| TCGA-DU-7290-01 | WT | WT | WT | WT | Clonal | WT | WT | WT |
| TCGA-DU-7294-01 | Clonal | WT | WT | Subclonal | WT | WT | WT | WT |
| TCGA-DU-7298-01 | Clonal | WT | WT | WT | WT | Subclonal | WT | WT |
| TCGA-DU-7299-01 | Clonal | WT | WT | WT | WT | WT | Clonal | WT |
| TCGA-DU-7300-01 | Subclonal | WT | WT | Subclonal | WT | WT | WT | WT |
| TCGA-DU-7301-01 | Subclonal | WT | WT | WT | WT | WT | WT | WT |
| TCGA-DU-7302-01 | Clonal | WT | WT | Subclonal | WT | WT | WT | WT |
| TCGA-DU-7304-01 | Clonal | WT | WT | WT | WT | WT | WT | WT |
| TCGA-DU-7304-02 | Clonal | WT | WT | WT | WT | WT | WT | WT |
| TCGA-DU-7306-01 | Subclonal | WT | WT | WT | WT | WT | WT | WT |
| TCGA-DU-7309-01 | WT | WT | WT | WT | WT | WT | WT | WT |
| TCGA-DU-8158-01 | WT | WT | Subclonal | WT | Subclonal | WT | WT | WT |
| TCGA-DU-8161-01 | WT | WT | WT | WT | Clonal | WT | WT | WT |
| TCGA-DU-8162-01 | WT | WT | Clonal | WT | WT | WT | WT | Subclonal |
| TCGA-DU-8163-01 | Clonal | WT | WT | WT | WT | WT | WT | WT |
| TCGA-DU-8164-01 | Subclonal | WT | WT | WT | WT | WT | WT | WT |
| TCGA-DU-8165-01 | WT | WT | WT | WT | WT | WT | WT | WT |
| TCGA-DU-8166-01 | Clonal | WT | WT | WT | WT | WT | WT | WT |
| TCGA-DU-8167-01 | Clonal | WT | WT | WT | WT | WT | WT | WT |
| TCGA-DU-8168-01 | Clonal | WT | WT | Subclonal | WT | WT | WT | WT |
| TCGA-DU-A5TP-01 | Clonal | WT | WT | WT | WT | WT | WT | WT |
| TCGA-DU-A5TR-01 | Clonal | WT | WT | WT | WT | WT | WT | WT |
| TCGA-DU-A5TS-01 | Clonal | WT | WT | WT | WT | WT | WT | WT |
| TCGA-DU-A5TT-01 | WT | WT | Clonal | WT | WT | WT | WT | WT |
| TCGA-DU-A5TU-01 | Clonal | WT | WT | WT | WT | WT | WT | WT |
| TCGA-DU-A5TW-01 | Clonal | Subclonal | WT | WT | WT | WT | WT | WT |
| TCGA-DU-A6S2-01 | Clonal | WT | WT | Clonal | WT | WT | WT | WT |
| TCGA-DU-A6S3-01 | Clonal | WT | WT | Clonal | WT | WT | WT | WT |
| TCGA-DU-A6S6-01 | Subclonal | WT | WT | WT | WT | WT | WT | WT |
| TCGA-DU-A6S7-01 | Clonal | WT | WT | WT | WT | WT | WT | WT |
| TCGA-DU-A6S8-01 | Clonal | WT | WT | Clonal | WT | WT | WT | WT |
| TCGA-DU-A76L-01 | WT | WT | WT | WT | WT | WT | Subclonal | WT |
| TCGA-DU-A76O-01 | Clonal | WT | WT | WT | WT | WT | WT | WT |
| TCGA-DU-A76R-01 | Clonal | WT | WT | WT | WT | WT | WT | WT |
| TCGA-DU-A7T6-01 | Clonal | WT | WT | Clonal | WT | WT | WT | WT |
| TCGA-DU-A7T8-01 | Clonal | WT | WT | WT | WT | WT | WT | WT |
| TCGA-DU-A7TA-01 | WT | WT | WT | WT | WT | WT | WT | WT |
| TCGA-DU-A7TB-01 | WT | WT | WT | WT | WT | WT | WT | WT |
| TCGA-DU-A7TC-01 | Clonal | WT | WT | WT | WT | WT | WT | WT |
| TCGA-DU-A7TD-01 | WT | WT | WT | WT | WT | WT | WT | WT |
| TCGA-DU-A7TG-01 | Subclonal | WT | WT | WT | WT | WT | WT | WT |
| TCGA-DU-A7TJ-01 | WT | WT | WT | WT | WT | WT | WT | WT |
| TCGA-E1-5302-01 | Subclonal | WT | WT | WT | WT | WT | WT | WT |
| TCGA-E1-5303-01 | Clonal | WT | WT | WT | WT | WT | WT | WT |
| TCGA-E1-5304-01 | Clonal | WT | WT | WT | WT | WT | WT | WT |
| TCGA-E1-5305-01 | Clonal | WT | WT | WT | WT | WT | WT | WT |
| TCGA-E1-5307-01 | Clonal | WT | WT | WT | WT | WT | WT | WT |
| TCGA-E1-5311-01 | Clonal | WT | WT | Clonal | WT | WT | WT | WT |
| TCGA-E1-5318-01 | WT | WT | WT | Clonal | Clonal | WT | WT | WT |
| TCGA-E1-5319-01 | Clonal | WT | WT | Subclonal | WT | WT | WT | WT |
| TCGA-E1-5322-01 | Clonal | WT | WT | WT | WT | WT | WT | WT |
| TCGA-E1-A7YD-01 | WT | WT | WT | WT | WT | Clonal | WT | Clonal |
| TCGA-E1-A7YE-01 | Subclonal | WT | WT | WT | WT | WT | WT | Subclonal |
| TCGA-E1-A7YH-01 | Clonal | WT | WT | WT | WT | WT | WT | WT |
| TCGA-E1-A7YI-01 | Subclonal | WT | WT | WT | WT | WT | WT | WT |
| TCGA-E1-A7YJ-01 | WT | WT | Subclonal | WT | WT | WT | WT | WT |
| TCGA-E1-A7YK-01 | Clonal | WT | WT | WT | WT | WT | WT | WT |
| TCGA-E1-A7YL-01 | WT | WT | WT | WT | WT | WT | WT | WT |
| TCGA-E1-A7YM-01 | WT | WT | Clonal | WT | WT | WT | WT | WT |
| TCGA-E1-A7YN-01 | WT | WT | WT | WT | WT | WT | WT | WT |
| TCGA-E1-A7YO-01 | Clonal | WT | WT | Subclonal | WT | WT | WT | WT |
| TCGA-E1-A7YQ-01 | WT | Subclonal | Clonal | WT | WT | WT | WT | WT |
| TCGA-E1-A7YS-01 | Clonal | WT | WT | WT | WT | WT | WT | WT |
| TCGA-E1-A7YU-01 | Clonal | WT | WT | WT | WT | WT | WT | WT |
| TCGA-E1-A7YV-01 | Clonal | WT | WT | WT | WT | WT | WT | WT |
| TCGA-E1-A7YW-01 | Clonal | WT | WT | WT | WT | WT | WT | Clonal |
| TCGA-E1-A7YY-01 | Clonal | WT | WT | Clonal | WT | WT | WT | WT |
| TCGA-E1-A7Z2-01 | WT | WT | Clonal | WT | WT | WT | WT | WT |
| TCGA-E1-A7Z3-01 | Clonal | WT | WT | WT | WT | WT | WT | WT |
| TCGA-E1-A7Z4-01 | Clonal | WT | WT | WT | WT | WT | WT | WT |
| TCGA-E1-A7Z6-01 | Clonal | WT | WT | WT | WT | WT | WT | WT |
| TCGA-EZ-7264-01 | Subclonal | WT | WT | Subclonal | WT | WT | WT | WT |
| TCGA-F6-A8O3-01 | Subclonal | WT | WT | WT | WT | WT | WT | WT |
| TCGA-F6-A8O4-01 | Subclonal | WT | WT | WT | WT | WT | WT | WT |
| TCGA-FG-5962-01 | Subclonal | WT | WT | Subclonal | WT | WT | WT | WT |
| TCGA-FG-5963-01 | WT | WT | WT | WT | WT | WT | WT | Clonal |
| TCGA-FG-5963-02 | WT | WT | WT | WT | WT | WT | Clonal | Clonal |
| TCGA-FG-5964-01 | Subclonal | WT | WT | Clonal | WT | WT | WT | WT |
| TCGA-FG-5965-01 | Clonal | WT | WT | WT | WT | WT | WT | WT |
| TCGA-FG-6688-01 | WT | WT | WT | WT | WT | WT | WT | Clonal |
| TCGA-FG-6689-01 | Clonal | WT | WT | WT | WT | WT | WT | WT |
| TCGA-FG-6690-01 | Clonal | WT | WT | WT | WT | WT | WT | WT |
| TCGA-FG-6691-01 | Clonal | WT | WT | WT | WT | WT | WT | WT |
| TCGA-FG-6692-01 | WT | WT | WT | WT | Subclonal | WT | WT | WT |
| TCGA-FG-7634-01 | Clonal | WT | WT | Subclonal | WT | WT | WT | WT |
| TCGA-FG-7636-01 | Clonal | WT | WT | WT | WT | WT | WT | WT |
| TCGA-FG-7638-01 | Clonal | WT | WT | Subclonal | WT | WT | WT | WT |
| TCGA-FG-7641-01 | Subclonal | WT | WT | Subclonal | WT | WT | WT | WT |
| TCGA-FG-7643-01 | WT | WT | WT | WT | WT | WT | WT | WT |
| TCGA-FG-8182-01 | Clonal | WT | WT | WT | WT | WT | WT | WT |
| TCGA-FG-8185-01 | Clonal | WT | WT | WT | WT | WT | WT | WT |
| TCGA-FG-8186-01 | Subclonal | WT | WT | WT | WT | WT | WT | WT |
| TCGA-FG-8187-01 | Subclonal | WT | WT | WT | WT | WT | WT | WT |
| TCGA-FG-8188-01 | Clonal | WT | WT | WT | WT | WT | WT | WT |
| TCGA-FG-8191-01 | Clonal | WT | WT | WT | WT | Clonal | WT | WT |
| TCGA-FG-A4MT-01 | Clonal | WT | WT | WT | WT | WT | WT | WT |
| TCGA-FG-A4MT-02 | Clonal | WT | WT | WT | WT | WT | WT | WT |
| TCGA-FG-A4MU-01 | WT | WT | WT | WT | WT | WT | WT | WT |
| TCGA-FG-A4MW-01 | WT | WT | WT | WT | Clonal | WT | WT | WT |
| TCGA-FG-A4MX-01 | Clonal | WT | WT | WT | WT | WT | WT | WT |
| TCGA-FG-A4MY-01 | Clonal | WT | WT | WT | WT | WT | WT | WT |
| TCGA-FG-A60J-01 | Clonal | WT | WT | WT | WT | WT | WT | Clonal |
| TCGA-FG-A60K-01 | Clonal | WT | WT | Clonal | WT | WT | WT | WT |
| TCGA-FG-A60L-01 | Clonal | WT | WT | WT | WT | WT | WT | WT |
| TCGA-FG-A6IZ-01 | Subclonal | WT | WT | Clonal | WT | WT | WT | WT |
| TCGA-FG-A6J1-01 | Clonal | WT | WT | Clonal | WT | Clonal | WT | WT |
| TCGA-FG-A6J3-01 | Clonal | WT | WT | WT | WT | WT | WT | WT |
| TCGA-FG-A70Y-01 | Clonal | WT | WT | WT | WT | WT | WT | WT |
| TCGA-FG-A70Z-01 | WT | WT | Clonal | WT | WT | WT | WT | WT |
| TCGA-FG-A710-01 | Subclonal | WT | WT | Clonal | WT | WT | WT | WT |
| TCGA-FG-A711-01 | Clonal | WT | WT | WT | WT | WT | WT | WT |
| TCGA-FG-A713-01 | Clonal | WT | WT | Clonal | WT | WT | WT | WT |
| TCGA-FG-A87N-01 | Subclonal | WT | WT | WT | WT | WT | Clonal | WT |
| TCGA-FG-A87Q-01 | WT | WT | WT | WT | WT | WT | WT | WT |
| TCGA-FN-7833-01 | Subclonal | WT | WT | WT | WT | WT | WT | WT |
| TCGA-HT-7467-01 | Clonal | WT | WT | Clonal | WT | WT | WT | WT |
| TCGA-HT-7468-01 | Subclonal | WT | WT | Subclonal | WT | WT | WT | WT |
| TCGA-HT-7469-01 | WT | WT | WT | WT | WT | WT | WT | WT |
| TCGA-HT-7470-01 | Clonal | WT | WT | WT | WT | Clonal | WT | WT |
| TCGA-HT-7471-01 | Clonal | WT | WT | Subclonal | WT | WT | WT | WT |
| TCGA-HT-7472-01 | Clonal | WT | WT | WT | WT | WT | WT | WT |
| TCGA-HT-7473-01 | Clonal | WT | WT | WT | WT | Subclonal | WT | WT |
| TCGA-HT-7474-01 | Clonal | WT | WT | WT | WT | WT | WT | WT |
| TCGA-HT-7475-01 | Subclonal | WT | WT | WT | WT | WT | WT | WT |
| TCGA-HT-7476-01 | WT | WT | WT | WT | WT | WT | WT | WT |
| TCGA-HT-7477-01 | Clonal | WT | WT | WT | WT | Clonal | WT | WT |
| TCGA-HT-7478-01 | Clonal | WT | WT | WT | WT | WT | WT | WT |
| TCGA-HT-7479-01 | Clonal | WT | WT | WT | WT | WT | WT | WT |
| TCGA-HT-7480-01 | Clonal | WT | WT | Subclonal | WT | WT | WT | WT |
| TCGA-HT-7481-01 | Subclonal | WT | WT | WT | WT | WT | WT | WT |
| TCGA-HT-7482-01 | Clonal | WT | WT | WT | WT | WT | WT | WT |
| TCGA-HT-7483-01 | Clonal | WT | WT | WT | WT | WT | WT | WT |
| TCGA-HT-7485-01 | Clonal | WT | WT | WT | WT | WT | WT | WT |
| TCGA-HT-7601-01 | Subclonal | WT | WT | WT | WT | WT | WT | WT |
| TCGA-HT-7602-01 | Clonal | WT | WT | WT | WT | WT | WT | WT |
| TCGA-HT-7603-01 | Clonal | WT | WT | WT | WT | WT | WT | WT |
| TCGA-HT-7604-01 | Clonal | WT | WT | WT | WT | WT | WT | WT |
| TCGA-HT-7605-01 | Clonal | WT | WT | WT | WT | WT | WT | WT |
| TCGA-HT-7606-01 | Clonal | WT | WT | WT | WT | WT | WT | WT |
| TCGA-HT-7607-01 | Clonal | WT | WT | WT | WT | WT | WT | WT |
| TCGA-HT-7608-01 | Subclonal | WT | WT | WT | WT | WT | Subclonal | WT |
| TCGA-HT-7609-01 | Clonal | WT | WT | WT | WT | WT | WT | WT |
| TCGA-HT-7610-01 | Clonal | WT | WT | WT | WT | WT | WT | WT |
| TCGA-HT-7611-01 | Clonal | WT | WT | WT | WT | WT | WT | WT |
| TCGA-HT-7616-01 | Clonal | WT | WT | Subclonal | WT | Subclonal | WT | WT |
| TCGA-HT-7620-01 | Clonal | WT | WT | WT | WT | WT | WT | WT |
| TCGA-HT-7676-01 | Clonal | WT | WT | WT | WT | WT | WT | WT |
| TCGA-HT-7677-01 | Clonal | WT | WT | Clonal | WT | WT | WT | WT |
| TCGA-HT-7681-01 | WT | WT | WT | Subclonal | WT | WT | WT | WT |
| TCGA-HT-7684-01 | Clonal | WT | WT | WT | WT | WT | WT | WT |
| TCGA-HT-7686-01 | Clonal | WT | WT | WT | WT | WT | WT | WT |
| TCGA-HT-7687-01 | Clonal | Clonal | WT | WT | WT | WT | WT | WT |
| TCGA-HT-7688-01 | Clonal | WT | WT | WT | WT | WT | WT | WT |
| TCGA-HT-7689-01 | Subclonal | WT | WT | WT | WT | WT | WT | WT |
| TCGA-HT-7690-01 | Clonal | WT | WT | WT | WT | WT | WT | WT |
| TCGA-HT-7692-01 | Subclonal | WT | WT | WT | WT | WT | WT | WT |
| TCGA-HT-7693-01 | Clonal | WT | WT | WT | WT | WT | WT | WT |
| TCGA-HT-7694-01 | Clonal | WT | WT | Subclonal | WT | WT | WT | WT |
| TCGA-HT-7695-01 | Clonal | WT | WT | Subclonal | WT | WT | WT | WT |
| TCGA-HT-7855-01 | Clonal | WT | WT | WT | WT | WT | WT | Subclonal |
| TCGA-HT-7856-01 | WT | WT | WT | WT | WT | WT | WT | WT |
| TCGA-HT-7857-01 | WT | WT | WT | WT | WT | WT | Clonal | WT |
| TCGA-HT-7858-01 | Subclonal | WT | WT | WT | WT | WT | WT | WT |
| TCGA-HT-7860-01 | WT | WT | WT | WT | Clonal | WT | Clonal | WT |
| TCGA-HT-7873-01 | Subclonal | WT | WT | WT | WT | WT | WT | WT |
| TCGA-HT-7874-01 | Clonal | WT | WT | WT | WT | WT | WT | WT |
| TCGA-HT-7875-01 | Clonal | Subclonal | WT | WT | WT | WT | WT | Subclonal |
| TCGA-HT-7877-01 | Clonal | WT | WT | Subclonal | WT | WT | WT | WT |
| TCGA-HT-7879-01 | Clonal | WT | WT | WT | WT | WT | WT | WT |
| TCGA-HT-7880-01 | Subclonal | WT | WT | WT | WT | WT | Subclonal | WT |
| TCGA-HT-7881-01 | Subclonal | WT | WT | WT | WT | WT | WT | WT |
| TCGA-HT-7882-01 | WT | WT | WT | WT | WT | WT | WT | WT |
| TCGA-HT-7884-01 | Clonal | WT | WT | WT | WT | WT | WT | WT |
| TCGA-HT-7902-01 | Clonal | Subclonal | WT | WT | WT | WT | WT | WT |
| TCGA-HT-8010-01 | Clonal | WT | WT | WT | WT | WT | Clonal | WT |
| TCGA-HT-8011-01 | WT | WT | WT | WT | WT | WT | WT | WT |
| TCGA-HT-8012-01 | Clonal | WT | WT | Subclonal | WT | WT | WT | WT |
| TCGA-HT-8013-01 | Clonal | WT | WT | WT | WT | Clonal | WT | WT |
| TCGA-HT-8018-01 | Clonal | WT | WT | WT | WT | WT | WT | WT |
| TCGA-HT-8104-01 | WT | WT | WT | WT | Clonal | WT | WT | WT |
| TCGA-HT-8105-01 | Clonal | WT | WT | Clonal | WT | WT | WT | WT |
| TCGA-HT-8106-01 | Clonal | Clonal | WT | WT | WT | WT | WT | WT |
| TCGA-HT-8107-01 | WT | WT | Clonal | WT | WT | WT | WT | WT |
| TCGA-HT-8108-01 | Clonal | WT | WT | WT | WT | WT | WT | WT |
| TCGA-HT-8109-01 | Clonal | WT | WT | WT | WT | WT | WT | WT |
| TCGA-HT-8110-01 | WT | WT | WT | WT | Clonal | WT | WT | WT |
| TCGA-HT-8111-01 | Clonal | WT | WT | WT | WT | WT | WT | WT |
| TCGA-HT-8113-01 | WT | WT | WT | WT | WT | WT | WT | WT |
| TCGA-HT-8114-01 | Clonal | WT | WT | WT | WT | WT | WT | WT |
| TCGA-HT-8563-01 | Clonal | WT | WT | WT | WT | WT | WT | WT |
| TCGA-HT-8564-01 | WT | WT | WT | WT | WT | WT | Clonal | Clonal |
| TCGA-HT-A4DS-01 | WT | WT | WT | WT | WT | WT | Subclonal | WT |
| TCGA-HT-A5R5-01 | Clonal | WT | WT | WT | WT | WT | WT | WT |
| TCGA-HT-A5R7-01 | Clonal | Subclonal | WT | WT | WT | WT | WT | WT |
| TCGA-HT-A5R9-01 | WT | WT | WT | Subclonal | WT | WT | WT | WT |
| TCGA-HT-A5RA-01 | WT | WT | WT | WT | Clonal | WT | WT | WT |
| TCGA-HT-A5RB-01 | Subclonal | WT | WT | WT | WT | WT | WT | WT |
| TCGA-HT-A5RC-01 | WT | WT | WT | WT | WT | WT | WT | Clonal |
| TCGA-HT-A614-01 | Clonal | WT | WT | WT | WT | WT | WT | WT |
| TCGA-HT-A615-01 | Clonal | WT | WT | WT | WT | WT | WT | WT |
| TCGA-HT-A616-01 | Clonal | WT | WT | WT | WT | WT | WT | Subclonal |
| TCGA-HT-A617-01 | WT | WT | WT | WT | WT | WT | WT | WT |
| TCGA-HT-A618-01 | WT | WT | WT | WT | WT | WT | WT | Subclonal |
| TCGA-HT-A619-01 | Clonal | WT | WT | Clonal | WT | WT | WT | WT |
| TCGA-HT-A61B-01 | Clonal | WT | WT | WT | WT | WT | WT | WT |
| TCGA-HT-A61C-01 | WT | WT | Clonal | WT | WT | WT | WT | WT |
| TCGA-HT-A74H-01 | WT | Subclonal | WT | WT | WT | Clonal | WT | WT |
| TCGA-HT-A74J-01 | Clonal | WT | WT | WT | WT | WT | WT | WT |
| TCGA-HT-A74K-01 | Clonal | WT | WT | WT | WT | WT | WT | WT |
| TCGA-HT-A74L-01 | Clonal | WT | WT | WT | WT | WT | WT | WT |
| TCGA-HT-A74O-01 | Clonal | WT | WT | WT | WT | WT | WT | WT |
| TCGA-HW-7486-01 | Subclonal | WT | WT | WT | WT | WT | WT | WT |
| TCGA-HW-7487-01 | Clonal | WT | WT | Clonal | WT | WT | WT | WT |
| TCGA-HW-7489-01 | Clonal | WT | WT | WT | WT | WT | WT | WT |
| TCGA-HW-7490-01 | Subclonal | WT | WT | WT | WT | WT | WT | WT |
| TCGA-HW-7491-01 | WT | WT | WT | Clonal | WT | WT | WT | WT |
| TCGA-HW-7495-01 | Clonal | WT | WT | Subclonal | WT | WT | WT | WT |
| TCGA-HW-8319-01 | Clonal | WT | WT | WT | WT | WT | WT | WT |
| TCGA-HW-8320-01 | Clonal | WT | WT | WT | WT | WT | WT | WT |
| TCGA-HW-8321-01 | Clonal | WT | WT | WT | WT | WT | WT | WT |
| TCGA-HW-8322-01 | Clonal | WT | WT | WT | WT | WT | WT | WT |
| TCGA-HW-A5KJ-01 | Clonal | WT | WT | WT | WT | WT | WT | WT |
| TCGA-HW-A5KK-01 | WT | WT | WT | WT | WT | WT | WT | WT |
| TCGA-HW-A5KL-01 | Clonal | WT | WT | WT | WT | WT | WT | WT |
| TCGA-HW-A5KM-01 | Clonal | WT | WT | WT | WT | WT | WT | WT |
| TCGA-IK-7675-01 | Clonal | WT | WT | WT | WT | WT | WT | WT |
| TCGA-IK-8125-01 | Subclonal | WT | WT | Subclonal | WT | WT | WT | WT |
| TCGA-KT-A74X-01 | Clonal | WT | WT | Clonal | WT | WT | WT | WT |
| TCGA-KT-A7W1-01 | WT | WT | WT | WT | WT | WT | WT | WT |
| TCGA-P5-A5ET-01 | Clonal | WT | WT | WT | WT | WT | WT | WT |
| TCGA-P5-A5EU-01 | Clonal | WT | WT | WT | WT | WT | WT | WT |
| TCGA-P5-A5EV-01 | Clonal | WT | WT | WT | WT | WT | WT | WT |
| TCGA-P5-A5EW-01 | Clonal | WT | WT | WT | WT | WT | WT | WT |
| TCGA-P5-A5EX-01 | Subclonal | WT | WT | WT | WT | WT | WT | WT |
| TCGA-P5-A5EZ-01 | Clonal | WT | WT | WT | WT | WT | WT | WT |
| TCGA-P5-A5F0-01 | Clonal | WT | WT | Subclonal | WT | WT | WT | WT |
| TCGA-P5-A5F1-01 | Clonal | WT | WT | WT | WT | WT | WT | WT |
| TCGA-P5-A5F2-01 | Clonal | WT | WT | WT | WT | WT | WT | WT |
| TCGA-P5-A5F4-01 | Clonal | WT | WT | WT | WT | WT | WT | WT |
| TCGA-P5-A72U-01 | WT | WT | WT | WT | WT | WT | WT | WT |
| TCGA-P5-A72W-01 | Clonal | WT | WT | WT | WT | WT | WT | WT |
| TCGA-P5-A72X-01 | Clonal | WT | WT | WT | WT | WT | WT | WT |
| TCGA-P5-A72Z-01 | Clonal | WT | WT | Clonal | WT | WT | WT | WT |
| TCGA-P5-A730-01 | WT | WT | WT | WT | WT | WT | WT | WT |
| TCGA-P5-A731-01 | Subclonal | WT | WT | WT | WT | WT | WT | WT |
| TCGA-P5-A733-01 | Clonal | WT | WT | WT | WT | WT | WT | WT |
| TCGA-P5-A735-01 | Clonal | WT | WT | WT | WT | WT | WT | WT |
| TCGA-P5-A736-01 | Clonal | WT | WT | WT | WT | Clonal | WT | WT |
| TCGA-P5-A737-01 | Clonal | WT | WT | WT | WT | WT | WT | WT |
| TCGA-P5-A77W-01 | Clonal | WT | WT | Clonal | WT | WT | WT | WT |
| TCGA-P5-A77X-01 | Clonal | WT | WT | Subclonal | WT | WT | WT | WT |
| TCGA-P5-A780-01 | Clonal | WT | WT | WT | WT | WT | WT | WT |
| TCGA-P5-A781-01 | Clonal | WT | WT | WT | WT | WT | WT | WT |
| TCGA-QH-A65R-01 | Clonal | WT | WT | WT | WT | WT | WT | WT |
| TCGA-QH-A65S-01 | Clonal | WT | WT | WT | WT | WT | WT | WT |
| TCGA-QH-A65V-01 | Clonal | WT | WT | Subclonal | WT | WT | WT | WT |
| TCGA-QH-A65X-01 | WT | WT | WT | Clonal | WT | WT | WT | WT |
| TCGA-QH-A65Z-01 | Clonal | WT | WT | Subclonal | WT | WT | WT | WT |
| TCGA-QH-A6CS-01 | WT | WT | WT | WT | Clonal | WT | Clonal | WT |
| TCGA-QH-A6CU-01 | Clonal | WT | WT | WT | WT | WT | WT | WT |
| TCGA-QH-A6CV-01 | WT | WT | WT | WT | WT | WT | WT | WT |
| TCGA-QH-A6CW-01 | Clonal | WT | WT | WT | WT | WT | WT | WT |
| TCGA-QH-A6CX-01 | WT | Clonal | Clonal | WT | Subclonal | WT | Clonal | WT |
| TCGA-QH-A6CY-01 | Clonal | WT | WT | Clonal | WT | WT | WT | WT |
| TCGA-QH-A6CZ-01 | Clonal | WT | WT | Clonal | WT | WT | WT | WT |
| TCGA-QH-A6X3-01 | Clonal | WT | WT | WT | WT | WT | WT | WT |
| TCGA-QH-A6X4-01 | Subclonal | WT | WT | WT | WT | WT | WT | WT |
| TCGA-QH-A6X5-01 | Subclonal | WT | WT | Subclonal | WT | WT | WT | WT |
| TCGA-QH-A6X8-01 | Clonal | WT | WT | Subclonal | WT | WT | WT | WT |
| TCGA-QH-A6X9-01 | Clonal | WT | WT | WT | WT | WT | WT | WT |
| TCGA-QH-A6XA-01 | Clonal | WT | WT | WT | WT | WT | WT | WT |
| TCGA-QH-A86X-01 | Clonal | WT | WT | Clonal | WT | WT | WT | WT |
| TCGA-QH-A870-01 | Clonal | WT | WT | WT | WT | WT | WT | WT |
| TCGA-R8-A6MK-01 | Clonal | WT | WT | WT | WT | WT | WT | WT |
| TCGA-R8-A6ML-01 | Clonal | WT | WT | Subclonal | WT | WT | WT | WT |
| TCGA-R8-A6MO-01 | Subclonal | WT | WT | Clonal | WT | Subclonal | WT | WT |
| TCGA-R8-A73M-01 | Clonal | WT | WT | Clonal | WT | WT | WT | WT |
| TCGA-RY-A83X-01 | Clonal | WT | WT | Subclonal | WT | WT | WT | WT |
| TCGA-RY-A83Y-01 | Clonal | WT | WT | Clonal | WT | WT | WT | WT |
| TCGA-RY-A83Z-01 | Subclonal | WT | WT | WT | WT | WT | WT | WT |
| TCGA-RY-A840-01 | Subclonal | WT | WT | WT | WT | WT | WT | WT |
| TCGA-RY-A843-01 | Clonal | WT | WT | WT | WT | WT | WT | WT |
| TCGA-RY-A845-01 | Clonal | WT | WT | WT | WT | WT | WT | WT |
| TCGA-RY-A847-01 | Clonal | WT | WT | WT | WT | WT | WT | WT |
| TCGA-S9-A6TS-01 | Clonal | Clonal | WT | WT | WT | WT | WT | WT |
| TCGA-S9-A6TU-01 | Clonal | WT | WT | WT | WT | WT | WT | WT |
| TCGA-S9-A6TV-01 | Clonal | WT | WT | WT | WT | Clonal | WT | WT |
| TCGA-S9-A6TW-01 | Clonal | WT | WT | Clonal | Subclonal | WT | WT | WT |
| TCGA-S9-A6TX-01 | Clonal | WT | WT | Subclonal | WT | WT | WT | WT |
| TCGA-S9-A6TY-01 | WT | WT | WT | WT | WT | WT | WT | WT |
| TCGA-S9-A6TZ-01 | Clonal | WT | WT | WT | WT | WT | WT | WT |
| TCGA-S9-A6U0-01 | WT | WT | WT | WT | Clonal | Subclonal | WT | WT |
| TCGA-S9-A6U1-01 | Clonal | WT | WT | WT | WT | WT | WT | WT |
| TCGA-S9-A6U2-01 | Subclonal | WT | WT | Clonal | WT | WT | WT | Clonal |
| TCGA-S9-A6U5-01 | Clonal | WT | WT | Subclonal | WT | WT | WT | WT |
| TCGA-S9-A6U6-01 | Clonal | WT | WT | WT | WT | WT | WT | WT |
| TCGA-S9-A6U8-01 | Clonal | WT | WT | WT | WT | WT | WT | WT |
| TCGA-S9-A6U9-01 | Clonal | WT | WT | WT | WT | WT | WT | WT |
| TCGA-S9-A6UA-01 | WT | WT | WT | WT | WT | WT | Clonal | WT |
| TCGA-S9-A6UB-01 | Clonal | WT | WT | Clonal | WT | WT | WT | WT |
| TCGA-S9-A6WD-01 | Clonal | WT | WT | WT | WT | WT | WT | WT |
| TCGA-S9-A6WE-01 | Clonal | WT | WT | Subclonal | WT | WT | WT | WT |
| TCGA-S9-A6WG-01 | Clonal | WT | WT | WT | WT | WT | WT | WT |
| TCGA-S9-A6WH-01 | Clonal | WT | WT | Clonal | WT | WT | WT | WT |
| TCGA-S9-A6WI-01 | Clonal | WT | WT | WT | WT | WT | WT | WT |
| TCGA-S9-A6WL-01 | Clonal | Subclonal | WT | WT | WT | WT | WT | WT |
| TCGA-S9-A6WM-01 | WT | WT | WT | WT | WT | Clonal | WT | WT |
| TCGA-S9-A6WN-01 | Clonal | WT | Clonal | WT | WT | WT | WT | WT |
| TCGA-S9-A6WO-01 | Clonal | WT | WT | WT | WT | WT | WT | WT |
| TCGA-S9-A6WP-01 | Clonal | WT | WT | WT | WT | WT | Clonal | WT |
| TCGA-S9-A6WQ-01 | Clonal | WT | WT | WT | WT | WT | WT | WT |
| TCGA-S9-A7IQ-01 | Subclonal | WT | WT | WT | WT | WT | WT | WT |
| TCGA-S9-A7IS-01 | Clonal | WT | WT | WT | WT | WT | WT | WT |
| TCGA-S9-A7IX-01 | WT | WT | WT | WT | WT | WT | Clonal | WT |
| TCGA-S9-A7IY-01 | Clonal | WT | WT | Subclonal | WT | WT | WT | WT |
| TCGA-S9-A7IZ-01 | Clonal | WT | WT | WT | WT | WT | WT | WT |
| TCGA-S9-A7J0-01 | Clonal | WT | WT | WT | WT | WT | WT | WT |
| TCGA-S9-A7J1-01 | Clonal | WT | WT | Subclonal | WT | WT | WT | WT |
| TCGA-S9-A7J2-01 | Clonal | WT | WT | Clonal | WT | WT | WT | WT |
| TCGA-S9-A7J3-01 | Subclonal | WT | WT | Subclonal | WT | WT | WT | WT |
| TCGA-S9-A7QW-01 | Clonal | WT | WT | WT | WT | WT | WT | WT |
| TCGA-S9-A7QX-01 | Clonal | WT | WT | WT | WT | WT | WT | WT |
| TCGA-S9-A7QY-01 | WT | WT | WT | WT | WT | WT | WT | WT |
| TCGA-S9-A7QZ-01 | Clonal | WT | WT | Clonal | WT | WT | WT | WT |
| TCGA-S9-A7R1-01 | Clonal | WT | WT | WT | WT | WT | WT | WT |
| TCGA-S9-A7R2-01 | WT | WT | Clonal | WT | WT | WT | WT | Subclonal |
| TCGA-S9-A7R3-01 | Clonal | WT | WT | WT | WT | WT | WT | WT |
| TCGA-S9-A7R4-01 | Clonal | WT | WT | WT | WT | WT | WT | WT |
| TCGA-S9-A7R7-01 | Clonal | WT | WT | WT | WT | WT | WT | WT |
| TCGA-S9-A7R8-01 | Subclonal | WT | WT | WT | WT | WT | WT | WT |
| TCGA-S9-A89V-01 | WT | WT | WT | WT | WT | WT | Clonal | WT |
| TCGA-S9-A89Z-01 | Subclonal | WT | WT | WT | WT | WT | WT | WT |
| TCGA-TM-A7C3-01 | WT | WT | WT | WT | WT | WT | WT | Clonal |
| TCGA-TM-A7C4-01 | Clonal | WT | WT | WT | WT | WT | WT | WT |
| TCGA-TM-A7C5-01 | Clonal | WT | WT | Subclonal | WT | WT | WT | WT |
| TCGA-TM-A7CA-01 | Clonal | WT | WT | WT | WT | Clonal | WT | WT |
| TCGA-TM-A7CF-01 | Clonal | WT | WT | WT | WT | WT | WT | WT |
| TCGA-TM-A7CF-02 | Subclonal | WT | WT | WT | WT | WT | WT | WT |
| TCGA-TM-A84B-01 | WT | WT | WT | WT | Clonal | WT | WT | WT |
| TCGA-TM-A84C-01 | WT | WT | WT | WT | WT | WT | Subclonal | WT |
| TCGA-TM-A84F-01 | Clonal | WT | WT | WT | WT | WT | WT | WT |
| TCGA-TM-A84G-01 | Clonal | WT | WT | WT | WT | WT | WT | WT |
| TCGA-TM-A84H-01 | Clonal | WT | WT | WT | WT | WT | WT | WT |
| TCGA-TM-A84I-01 | Clonal | WT | WT | WT | WT | WT | WT | WT |
| TCGA-TM-A84J-01 | WT | WT | WT | WT | WT | WT | WT | WT |
| TCGA-TM-A84L-01 | Clonal | WT | WT | WT | WT | WT | WT | WT |
| TCGA-TM-A84M-01 | Clonal | WT | WT | Subclonal | WT | WT | WT | WT |
| TCGA-TM-A84O-01 | Clonal | WT | WT | Subclonal | WT | WT | WT | WT |
| TCGA-TM-A84Q-01 | Clonal | WT | WT | WT | WT | WT | WT | WT |
| TCGA-TM-A84R-01 | WT | WT | WT | WT | WT | WT | WT | WT |
| TCGA-TM-A84S-01 | Clonal | WT | WT | WT | WT | WT | WT | WT |
| TCGA-TM-A84T-01 | Clonal | WT | WT | WT | WT | WT | WT | WT |
| TCGA-TQ-A7RF-01 | Clonal | WT | WT | WT | WT | WT | WT | WT |
| TCGA-TQ-A7RG-01 | Clonal | WT | WT | Subclonal | WT | WT | WT | WT |
| TCGA-TQ-A7RH-01 | Clonal | WT | WT | WT | WT | WT | WT | WT |
| TCGA-TQ-A7RI-01 | Clonal | WT | WT | Subclonal | WT | WT | WT | WT |
| TCGA-TQ-A7RJ-01 | Clonal | WT | WT | WT | WT | WT | WT | WT |
| TCGA-TQ-A7RK-01 | Clonal | WT | WT | WT | WT | WT | WT | WT |
| TCGA-TQ-A7RM-01 | Clonal | WT | WT | WT | WT | WT | WT | WT |
| TCGA-TQ-A7RN-01 | Clonal | WT | WT | WT | WT | WT | WT | WT |
| TCGA-TQ-A7RO-01 | WT | WT | WT | Clonal | WT | WT | WT | WT |
| TCGA-TQ-A7RP-01 | WT | WT | Clonal | WT | Clonal | Clonal | Clonal | WT |
| TCGA-TQ-A7RQ-01 | Clonal | WT | WT | Clonal | WT | WT | WT | WT |
| TCGA-TQ-A7RR-01 | Clonal | WT | WT | WT | WT | WT | WT | WT |
| TCGA-TQ-A7RS-01 | Clonal | WT | WT | WT | WT | WT | WT | WT |
| TCGA-TQ-A7RU-01 | Clonal | WT | WT | Subclonal | WT | WT | WT | WT |
| TCGA-TQ-A7RV-01 | Clonal | WT | WT | WT | WT | WT | WT | WT |
| TCGA-TQ-A7RV-02 | Clonal | WT | WT | WT | WT | WT | WT | WT |
| TCGA-TQ-A7RW-01 | Clonal | WT | WT | WT | WT | WT | WT | WT |
| TCGA-TQ-A8XE-01 | Subclonal | WT | WT | WT | WT | WT | WT | WT |
| TCGA-TQ-A8XE-02 | Subclonal | WT | WT | WT | WT | WT | WT | WT |
| TCGA-VM-A8C8-01 | Clonal | WT | WT | WT | WT | WT | WT | WT |
| TCGA-VM-A8C9-01 | WT | WT | WT | WT | WT | WT | WT | WT |
| TCGA-VM-A8CA-01 | Clonal | WT | WT | WT | WT | WT | WT | WT |
| TCGA-VM-A8CB-01 | Subclonal | WT | WT | Subclonal | WT | WT | WT | WT |
| TCGA-VM-A8CD-01 | WT | WT | Subclonal | WT | Clonal | WT | Subclonal | WT |
| TCGA-VM-A8CE-01 | Subclonal | WT | WT | Subclonal | WT | WT | WT | WT |
| TCGA-VM-A8CF-01 | Clonal | WT | WT | WT | WT | WT | WT | Clonal |
| TCGA-VM-A8CH-01 | Clonal | WT | WT | WT | WT | WT | WT | WT |
| TCGA-VV-A829-01 | Subclonal | WT | WT | WT | WT | WT | WT | Clonal |
| TCGA-VV-A86M-01 | Clonal | WT | WT | WT | WT | WT | WT | WT |
| TCGA-VW-A7QS-01 | Subclonal | WT | WT | WT | WT | WT | WT | WT |
| TCGA-VW-A8FI-01 | WT | WT | WT | WT | WT | WT | WT | WT |
| TCGA-W9-A837-01 | Clonal | WT | WT | WT | WT | WT | WT | WT |
| TCGA-WH-A86K-01 | Clonal | WT | WT | WT | WT | WT | WT | WT |
| TCGA-WY-A858-01 | Clonal | WT | WT | WT | WT | WT | WT | WT |
| TCGA-WY-A859-01 | Clonal | WT | WT | WT | WT | WT | WT | WT |
| TCGA-WY-A85A-01 | Clonal | WT | WT | WT | WT | WT | WT | WT |
| TCGA-WY-A85B-01 | Clonal | WT | WT | WT | WT | WT | WT | WT |
| TCGA-WY-A85C-01 | Clonal | WT | WT | WT | WT | WT | WT | WT |
| TCGA-WY-A85D-01 | Clonal | WT | WT | WT | WT | WT | WT | WT |
| TCGA-WY-A85E-01 | Clonal | WT | WT | WT | WT | WT | WT | WT |
