# Supplementary material for: Dissecting and analyzing the Subclonal Mutations Associated with Poor Prognosis in Diffuse Glioma
Source: Biomed Res Int. 2022 Apr 18;2022:4919111. doi: 10.1155/2022/4919111 (PMC9039777; doi:10.1155/2022/4919111)
Supplement: Supplementary 3 — Supplementary Table 2: the dysregulated ceRNA networks driven by risk subclonal mutations in GBM and LGG. [file 4919111.f3.docx]

**GBM**

| gene1 | gene2 | deltac | mir |
| --- | --- | --- | --- |
| AHNAK | LINC01320 | 0.006008 | hsa-miR-186-5p |
| AHNAK | SEMA6D | 0.008608 | hsa-miR-186-5p |
| AHNAK | DSP | 0.003103 | hsa-miR-186-5p |
| AHNAK | NEK9 | 0.012964 | hsa-miR-186-5p |
| AHNAK | ALDH5A1 | 0.004046 | hsa-miR-186-5p |
| AHNAK | ZMAT3 | 0.006486 | hsa-miR-186-5p |
| AHNAK | EFNA5 | 0.009513 | hsa-miR-186-5p |
| AHNAK | TGFB2 | 0.008019 | hsa-miR-186-5p |
| AHNAK | C2orf72 | 0.023002 | hsa-miR-186-5p |
| AHNAK | SUDS3 | 0.014425 | hsa-miR-186-5p |
| AHNAK | HMGA2 | 0.016147 | hsa-miR-186-5p |
| AHNAK | MALAT1 | 0.008768 | hsa-miR-181b-5p |
| AHNAK | SH2D4A | 0.005117 | hsa-miR-181b-5p |
| AHNAK | 11-Sep | 0.010128 | hsa-miR-181b-5p |
| AHNAK | C3orf58 | 0.010711 | hsa-miR-181b-5p |
| AHNAK | RNF213 | 0.003524 | hsa-miR-181b-5p |
| AHNAK | PIK3C2A | 0.003997 | hsa-miR-181b-5p |
| AHNAK | PSD3 | 0.012574 | hsa-miR-181b-5p |
| AHNAK | SYT15 | 0.014727 | hsa-miR-181b-5p |
| AHNAK | MTX3 | 0.00751 | hsa-miR-181b-5p |
| AHNAK | GALNT4 | 0.008633 | hsa-miR-181b-5p |
| AHNAK | NEO1 | 0.009822 | hsa-miR-181b-5p |
| AHNAK | MYO1E | 0.00635 | hsa-miR-181b-5p |
| AHNAK | TMED7-TICAM2 | 0.005625 | hsa-miR-181b-5p |
| AHNAK | SLC5A3 | 0.007417 | hsa-miR-181a-5p |
| AHNAK | GXYLT1 | 0.009919 | hsa-miR-181a-5p |
| AHNAK | FAM225B | 0.017406 | hsa-miR-326 |
| AHNAK | LINC01035 | 0.010673 | hsa-miR-326 |
| AHNAK | LINC01219 | 0.014583 | hsa-miR-326 |
| AHNAK | AC009502.4 | 0.017993 | hsa-miR-326 |
| AHNAK | LINC00837 | 0.008842 | hsa-miR-326 |
| AHNAK | RP11-61A14.4 | 0.008492 | hsa-miR-326 |
| AHNAK | RP11-893F2.5 | 0.007716 | hsa-miR-326 |
| AHNAK | LINC00930 | 0.001425 | hsa-miR-326 |
| AHNAK | RP11-430B1.2 | 0.00998 | hsa-miR-326 |
| AHNAK | CTC-559E9.4 | 0.017152 | hsa-miR-326 |
| AHNAK | SLAMF1 | 0.002351 | hsa-miR-326 |
| AHNAK | GAB1 | 0.001985 | hsa-miR-326 |
| AHNAK | ITPKA | 0.011931 | hsa-miR-326 |
| AHNAK | RP11-680C21.1 | 0.001214 | hsa-miR-20a-5p |
| AHNAK | TMTC1 | 0.00379 | hsa-miR-20a-5p |
| AHNAK | FSTL5 | 0.011127 | hsa-miR-20a-5p |
| AHNAK | DTWD2 | 0.011707 | hsa-miR-20a-5p |
| AHNAK | LYPD6 | 0.017684 | hsa-miR-433-3p |
| AHNAK | KIAA1671 | 0.00308 | hsa-miR-433-3p |
| AHNAK | F2RL1 | 0.011719 | hsa-miR-433-3p |
| AHNAK | TMEM170A | 0.012336 | hsa-miR-154-5p |
| AHNAK | NAV2 | 0.000901 | hsa-miR-154-5p |
| AHNAK | GPR137B | 0.011177 | hsa-miR-154-5p |
| AHNAK | CASC4 | 0.008127 | hsa-miR-154-5p |
| AHNAK | ENDOD1 | 0.014293 | hsa-miR-211-5p |
| AHNAK | WNT10B | 0.016141 | hsa-miR-211-5p |
| AHNAK | SV2B | 0.002615 | hsa-miR-211-5p |
| AHNAK | RBM20 | 0.004167 | hsa-miR-211-5p |
| AHNAK | SCN2B | 0.012037 | hsa-miR-211-5p |
| AHNAK | RHOF | 0.007334 | hsa-miR-519d-3p |
| AHNAK | CHD5 | 0.008112 | hsa-miR-519d-3p |
| AHNAK | TP53BP2 | 0.013659 | hsa-miR-372-3p |
| AHNAK | ROCK2 | 0.006277 | hsa-miR-372-3p |
| AHNAK | PPM1H | 0.000831 | hsa-miR-372-3p |
| AHNAK | PCNX | 0.005398 | hsa-miR-106b-5p |
| AHNAK | LINC00472 | 0.007988 | hsa-miR-204-5p |
| AHNAK | LRP2 | 0.024077 | hsa-miR-424-5p |
| AHNAK | RP5-942I16.1 | 0.004106 | hsa-miR-506-3p |
| AHNAK | CTD-2246P4.1 | 0.000132 | hsa-miR-506-3p |
| AHNAK | CELSR1 | 0.007831 | hsa-miR-506-3p |
| AHNAK2 | MIR22HG | 0.000427 | hsa-miR-195-5p |
| AHNAK2 | DCBLD2 | 0.001764 | hsa-miR-195-5p |
| AHNAK2 | HGF | 0.007351 | hsa-miR-195-5p |
| AHNAK2 | TRIM59 | 0.000946 | hsa-miR-195-5p |
| AHNAK2 | MET | 0.006126 | hsa-miR-195-5p |
| AHNAK2 | PAWR | 0.006537 | hsa-miR-195-5p |
| AHNAK2 | HMGA2 | 0.004252 | hsa-miR-195-5p |
| AHNAK2 | PLXDC2 | 0.006981 | hsa-miR-497-5p |
| AHNAK2 | CREG1 | 0.002333 | hsa-miR-497-5p |
| AHNAK2 | NIPAL4 | 0.011006 | hsa-miR-497-5p |
| AHNAK2 | VTI1B | 0.014806 | hsa-miR-497-5p |
| AHNAK2 | SAT1 | 0.001917 | hsa-miR-497-5p |
| AHNAK2 | AFTPH | 0.017422 | hsa-miR-33b-5p |
| AHNAK2 | MID1 | 0.011836 | hsa-miR-33b-5p |
| AHNAK2 | DKK1 | 0.010901 | hsa-miR-33b-5p |
| AHNAK2 | SLCO3A1 | 0.008347 | hsa-miR-15b-5p |

**LGG**

| gene1 | gene2 | deltac | mir |
| --- | --- | --- | --- |
| NF1 | LINC00662 | 0.007398 | hsa-miR-34a-5p |
| NF1 | LINC00483 | 0.000111 | hsa-miR-34a-5p |
| NF1 | RP11-314B1.2 | 0.004336 | hsa-miR-34a-5p |
| NF1 | RP11-981G7.6 | 0.00507 | hsa-miR-34a-5p |
| NF1 | NDRG3 | 0.00744 | hsa-miR-34a-5p |
| NF1 | CTDSP2 | 0.011197 | hsa-miR-34a-5p |
| NF1 | UBE2H | 0.009853 | hsa-miR-34a-5p |
| NF1 | CRKL | 0.003047 | hsa-miR-34a-5p |
| NF1 | USP6NL | 0.010158 | hsa-miR-34a-5p |
| NF1 | SRSF3 | 0.004197 | hsa-miR-34a-5p |
| NF1 | RB1 | 0.005155 | hsa-miR-34a-5p |
| NF1 | UBR1 | 0.000842 | hsa-miR-34a-5p |
| NF1 | TXNL1 | 0.008817 | hsa-miR-34a-5p |
| NF1 | ZC3H7B | 0.00867 | hsa-miR-34a-5p |
| NF1 | DDX6 | 0.000177 | hsa-miR-34a-5p |
| NF1 | SKI | 0.007704 | hsa-miR-34a-5p |
| NF1 | FAM160B1 | 0.002061 | hsa-miR-34a-5p |
| NF1 | BNIP2 | 0.008859 | hsa-miR-34a-5p |
| NF1 | PKIA | 0.001734 | hsa-miR-34a-5p |
| NF1 | REEP1 | 0.001573 | hsa-miR-34a-5p |
| NF1 | ADO | 0.00198 | hsa-miR-34a-5p |
| NF1 | ZNF879 | 0.009316 | hsa-miR-34a-5p |
| NF1 | ADAMTS5 | 0.004294 | hsa-miR-34a-5p |
| NF1 | PPP1CB | 0.003824 | hsa-miR-34a-5p |
| NF1 | TLL1 | 0.004166 | hsa-miR-34a-5p |
| NF1 | UBE2O | 0.007666 | hsa-miR-34a-5p |
| NF1 | ZDHHC9 | 0.002593 | hsa-miR-34a-5p |
| NF1 | NIN | 0.006237 | hsa-miR-34a-5p |
| NF1 | UBN2 | 0.008475 | hsa-miR-34a-5p |
| NF1 | SUPT16H | 0.003974 | hsa-miR-34a-5p |
| NF1 | ATG5 | 0.004129 | hsa-miR-34a-5p |
| NF1 | RAPGEF6 | 0.004585 | hsa-miR-34a-5p |
| NF1 | WASF1 | 0.002512 | hsa-miR-34a-5p |
| NF1 | CMPK1 | 0.00975 | hsa-miR-34a-5p |
| NF1 | YY1 | 0.008599 | hsa-miR-34a-5p |
| NF1 | SOX4 | 0.00058 | hsa-miR-34a-5p |
| NF1 | DIP2A | 0.003168 | hsa-miR-34a-5p |
| NF1 | CCNDBP1 | 0.006503 | hsa-miR-34a-5p |
| NF1 | RANBP10 | 0.003722 | hsa-miR-34a-5p |
| NF1 | KAT7 | 0.002859 | hsa-miR-34a-5p |
| NF1 | TSPAN14 | 0.00532 | hsa-miR-34a-5p |
| NF1 | FGFR1OP2 | 0.001012 | hsa-miR-34a-5p |
| NF1 | TCF12 | 0.005058 | hsa-miR-34a-5p |
| NF1 | PCMTD2 | 0.010775 | hsa-miR-34a-5p |
| NF1 | STAG2 | 0.004263 | hsa-miR-34a-5p |
| NF1 | STX17 | 0.005472 | hsa-miR-34a-5p |
| NF1 | SEC23A | 0.009074 | hsa-miR-34a-5p |
| NF1 | ZNF189 | 0.010779 | hsa-miR-34a-5p |
| NF1 | RAB11FIP3 | 0.000685 | hsa-miR-34a-5p |
| NF1 | MAP4K4 | 0.007528 | hsa-miR-34a-5p |
| NF1 | ACOX1 | 0.011812 | hsa-miR-34a-5p |
| NF1 | ARHGEF12 | 0.01029 | hsa-miR-34a-5p |
| NF1 | ZNF614 | 0.008018 | hsa-miR-34a-5p |
| NF1 | ZNF776 | 0.014206 | hsa-miR-34a-5p |
| NF1 | ZNF10 | 0.006676 | hsa-miR-34a-5p |
| NF1 | INCENP | 0.003003 | hsa-miR-34a-5p |
| NF1 | PIGS | 0.003889 | hsa-miR-34a-5p |
| NF1 | HACE1 | 0.006373 | hsa-miR-34a-5p |
| NF1 | CORO1C | 0.007884 | hsa-miR-34a-5p |
| NF1 | DCTN2 | 0.016478 | hsa-miR-34a-5p |
| NF1 | SCD5 | 0.010819 | hsa-miR-34a-5p |
| NF1 | HECTD1 | 0.00085 | hsa-miR-34a-5p |
| NF1 | RSBN1 | 0.006556 | hsa-miR-34a-5p |
| NF1 | SRPK2 | 0.0013 | hsa-miR-34a-5p |
| NF1 | EPB41L4A-AS1 | 0.003444 | hsa-miR-30e-5p |
| NF1 | RP11-739N10.1 | 0.004021 | hsa-miR-30e-5p |
| NF1 | SON | 0.004659 | hsa-miR-30e-5p |
| NF1 | CCDC120 | 0.002605 | hsa-miR-30e-5p |
| NF1 | PIP4K2B | 0.001284 | hsa-miR-30e-5p |
| NF1 | RAB1A | 0.005848 | hsa-miR-30e-5p |
| NF1 | SPAST | 0.005079 | hsa-miR-30e-5p |
| NF1 | ATAD2B | 0.002244 | hsa-miR-30e-5p |
| NF1 | DCAF5 | 0.007464 | hsa-miR-30e-5p |
| NF1 | DENND5B | 0.005482 | hsa-miR-30e-5p |
| NF1 | MTPAP | 0.001262 | hsa-miR-30e-5p |
| NF1 | MAP3K13 | 0.010412 | hsa-miR-30e-5p |
| NF1 | SPG11 | 0.000718 | hsa-miR-30e-5p |
| NF1 | C1orf131 | 0.008139 | hsa-miR-30e-5p |
| NF1 | SAFB2 | 0.000179 | hsa-miR-30e-5p |
| NF1 | GSK3B | 0.001288 | hsa-miR-30e-5p |
| NF1 | C6orf120 | 0.006191 | hsa-miR-30e-5p |
| NF1 | TBPL1 | 0.001659 | hsa-miR-30e-5p |
| NF1 | CECR6 | 0.001759 | hsa-miR-30e-5p |
| NF1 | RPAIN | 0.006514 | hsa-miR-30e-5p |
| NF1 | QSOX2 | 0.001991 | hsa-miR-30e-5p |
| NF1 | PPP2R5E | 0.002237 | hsa-miR-30e-5p |
| NF1 | FLVCR2 | 0.002109 | hsa-miR-30e-5p |
| NF1 | DLG5 | 0.00277 | hsa-miR-30e-5p |
| NF1 | MMGT1 | 0.003635 | hsa-miR-30e-5p |
| NF1 | CEP350 | 0.001521 | hsa-miR-30e-5p |
| NF1 | PNN | 0.01197 | hsa-miR-30e-5p |
| NF1 | BPTF | 0.004207 | hsa-miR-30e-5p |
| NF1 | MTX3 | 0.011317 | hsa-miR-30e-5p |
| NF1 | ZNF804A | 0.006795 | hsa-miR-30e-5p |
| NF1 | MBD1 | 0.008963 | hsa-miR-30e-5p |
| NF1 | PNPT1 | 0.007704 | hsa-miR-30e-5p |
| NF1 | FAM168B | 0.003041 | hsa-miR-30e-5p |
| NF1 | RP2 | 0.005583 | hsa-miR-30e-5p |
| NF1 | RP11-728E14.3 | 0.010205 | hsa-miR-30c-5p |
| NF1 | SLTM | 0.008056 | hsa-miR-30c-5p |
| NF1 | IQGAP2 | 0.003626 | hsa-miR-30c-5p |
| NF1 | GM2A | 0.014242 | hsa-miR-30c-5p |
| NF1 | C15orf57 | 0.012832 | hsa-miR-30c-5p |
| NF1 | GRB2 | 0.011668 | hsa-miR-30c-5p |
| NF1 | PLA2G12A | 0.009049 | hsa-miR-30c-5p |
| NF1 | UBQLN4 | 0.003084 | hsa-miR-30c-5p |
| NF1 | MAP3K1 | 0.001705 | hsa-miR-30c-5p |
| NF1 | DLD | 0.017639 | hsa-miR-30c-5p |
| NF1 | RANBP9 | 0.011032 | hsa-miR-30c-5p |
| NF1 | MYH10 | 0.003292 | hsa-miR-30c-5p |
| NF1 | ITSN2 | 0.003341 | hsa-miR-30c-5p |
| NF1 | YME1L1 | 0.001769 | hsa-miR-30c-5p |
| NF1 | FBXW7 | 0.009229 | hsa-miR-30c-5p |
| NF1 | DNAJC3 | 0.016034 | hsa-miR-30c-5p |
| NF1 | ATP6V1B2 | 0.004238 | hsa-miR-30c-5p |
| NF1 | ALKBH1 | 0.006033 | hsa-miR-30c-5p |
| NF1 | RGL1 | 0.011456 | hsa-miR-30c-5p |
| NF1 | SNTB2 | 0.000681 | hsa-miR-30c-5p |
| NF1 | MAP3K2 | 0.001808 | hsa-miR-30c-5p |
| NF1 | NDEL1 | 0.007214 | hsa-miR-30c-5p |
| NF1 | ARL6 | 0.002175 | hsa-miR-30c-5p |
| NF1 | FAM49B | 0.01307 | hsa-miR-30c-5p |
| NF1 | CPNE2 | 0.009993 | hsa-miR-30c-5p |
| NF1 | FCHSD2 | 0.0047 | hsa-miR-137 |
| NF1 | ZNF512 | 0.004905 | hsa-miR-137 |
| NF1 | AKAP2 | 0.012136 | hsa-miR-137 |
| NF1 | TET2 | 0.004689 | hsa-miR-137 |
| NF1 | MTF1 | 0.00417 | hsa-miR-137 |
| NF1 | TBCE | 0.001342 | hsa-miR-137 |
| NF1 | PIGG | 0.007523 | hsa-miR-137 |
| NF1 | ERLIN2 | 0.002141 | hsa-miR-137 |
| NF1 | COBLL1 | 0.00551 | hsa-miR-137 |
| NF1 | RAP1B | 0.017323 | hsa-miR-137 |
| NF1 | ERGIC2 | 0.005595 | hsa-miR-137 |
| NF1 | CD2AP | 0.003881 | hsa-miR-137 |
| NF1 | MTF2 | 0.004383 | hsa-miR-137 |
| NF1 | HERPUD2 | 0.014093 | hsa-miR-137 |
| NF1 | FRYL | 0.004455 | hsa-miR-137 |
| NF1 | AGGF1 | 0.000378 | hsa-miR-137 |
| NF1 | HNRNPU | 0.002642 | hsa-miR-137 |
| NF1 | EPS8 | 0.014107 | hsa-miR-137 |
| NF1 | ITGB8 | 0.019381 | hsa-miR-137 |
| NF1 | KIAA0907 | 0.008496 | hsa-miR-137 |
| NF1 | CNOT8 | 0.000464 | hsa-miR-137 |
| NF1 | FAM110B | 0.002454 | hsa-miR-137 |
| NF1 | ZDHHC3 | 0.011971 | hsa-miR-137 |
| NF1 | VPS45 | 0.003999 | hsa-miR-137 |
| NF1 | KCTD20 | 0.000312 | hsa-miR-199a-5p |
| NF1 | HIRA | 0.004351 | hsa-miR-199a-5p |
| NF1 | PANK3 | 0.003496 | hsa-miR-199a-5p |
| NF1 | GRIP1 | 0.005986 | hsa-miR-199a-5p |
| NF1 | USPL1 | 0.008298 | hsa-miR-199a-5p |
| NF1 | AKAP13 | 0.004543 | hsa-miR-199a-5p |
| NF1 | IGDCC3 | 0.013155 | hsa-miR-199a-5p |
| NF1 | TBC1D5 | 0.005074 | hsa-miR-199a-5p |
| NF1 | ABHD4 | 0.003684 | hsa-miR-199a-5p |
| NF1 | SNAPIN | 0.004351 | hsa-miR-199a-5p |
| NF1 | ZNF655 | 0.001971 | hsa-miR-135b-5p |
| NF1 | EXOC2 | 0.000809 | hsa-miR-135b-5p |
| NF1 | BTD | 0.002919 | hsa-miR-135b-5p |
| NF1 | SMAD3 | 0.01026 | hsa-miR-135b-5p |
| NF1 | HIPK3 | 0.008998 | hsa-miR-135b-5p |
| NF1 | ZNF708 | 0.003556 | hsa-miR-135b-5p |
| NF1 | EIF4B | 0.00229 | hsa-miR-135b-5p |
| NF1 | ERCC5 | 0.005653 | hsa-miR-135b-5p |
| NF1 | WNT7B | 0.00707 | hsa-miR-135b-5p |
| NF1 | UBLCP1 | 0.002738 | hsa-miR-34c-5p |
| NF1 | LPP | 0.01129 | hsa-miR-34c-5p |
| NF1 | 11-Sep | 0.00811 | hsa-miR-34c-5p |
| NF1 | GLRB | 0.009925 | hsa-miR-34c-5p |
| NF1 | THRB | 0.001084 | hsa-miR-34c-5p |
| NF1 | SRSF10 | 0.004758 | hsa-miR-34c-5p |
| NF1 | DNAJC16 | 0.004723 | hsa-miR-34c-5p |
| NF1 | THAP2 | 0.002207 | hsa-miR-34c-5p |
| NF1 | DEF8 | 0.008298 | hsa-miR-34c-5p |
| NF1 | CAMTA2 | 0.005985 | hsa-miR-34c-5p |
| NF1 | FOXF2 | 0.007095 | hsa-miR-34c-5p |
| NF1 | HBP1 | 0.000364 | hsa-miR-34c-5p |
| NF1 | GALNT13 | 0.005902 | hsa-let-7i-5p |
| NF1 | SPATA7 | 0.003754 | hsa-let-7i-5p |
| NF1 | TRAF3 | 0.008092 | hsa-let-7i-5p |
| NF1 | SRSF6 | 0.009378 | hsa-let-7i-5p |
| NF1 | LDOC1 | 0.011772 | hsa-let-7i-5p |
| NF1 | RAB40C | 0.00804 | hsa-let-7i-5p |
| NF1 | PRRX1 | 0.00619 | hsa-let-7i-5p |
| NF1 | DAGLA | 0.006193 | hsa-let-7i-5p |
| NF1 | ATP2B4 | 0.002832 | hsa-miR-135a-5p |
| NF1 | BHLHB9 | 0.006589 | hsa-miR-135a-5p |
| NF1 | JAK2 | 0.00344 | hsa-miR-135a-5p |
| NF1 | WDR20 | 0.004741 | hsa-miR-135a-5p |
| NF1 | RFX5 | 0.001315 | hsa-miR-135a-5p |
| NF1 | CALM1 | 0.00351 | hsa-miR-135a-5p |
| NF1 | ARHGEF3 | 0.014584 | hsa-miR-135a-5p |
| NF1 | DNAJC5 | 0.010767 | hsa-miR-135a-5p |
| NF1 | THAP5 | 0.004203 | hsa-miR-135a-5p |
| NF1 | PITPNA | 0.002723 | hsa-miR-135a-5p |
| NF1 | ZNF223 | 0.006954 | hsa-miR-135a-5p |
| NF1 | IL6ST | 0.011442 | hsa-miR-135a-5p |
| NF1 | USP10 | 0.00467 | hsa-miR-135a-5p |
| NF1 | ZFP14 | 0.010195 | hsa-miR-135a-5p |
| NF1 | MCL1 | 0.016844 | hsa-miR-135a-5p |
| NF1 | KLHL15 | 0.010115 | hsa-miR-135a-5p |
| NF1 | ATP6AP2 | 0.009326 | hsa-miR-135a-5p |
| NF1 | PICALM | 0.010273 | hsa-miR-135a-5p |
| NF1 | LRPPRC | 0.008867 | hsa-miR-135a-5p |
| NF1 | LINC00176 | 0.006223 | hsa-miR-16-5p |
| NF1 | JAKMIP2 | 0.004849 | hsa-miR-16-5p |
| NF1 | UQCRC2 | 0.002657 | hsa-miR-16-5p |
| NF1 | RCL1 | 0.001463 | hsa-miR-16-5p |
| NF1 | OLFM1 | 0.009557 | hsa-miR-16-5p |
| NF1 | FBXO21 | 0.001055 | hsa-miR-16-5p |
| NF1 | PCDHA6 | 0.003274 | hsa-miR-16-5p |
| NF1 | SFXN5 | 0.014804 | hsa-miR-16-5p |
| NF1 | SYDE2 | 0.001768 | hsa-miR-16-5p |
| NF1 | RAD50 | 0.002308 | hsa-miR-16-5p |
| NF1 | SALL1 | 0.017254 | hsa-miR-16-5p |
| NF1 | CDIPT | 0.000251 | hsa-miR-16-5p |
| NF1 | TSN | 0.015214 | hsa-miR-16-5p |
| NF1 | FASN | 0.000767 | hsa-miR-16-5p |
| NF1 | NEUROD1 | 0.011153 | hsa-miR-16-5p |
| NF1 | PLRG1 | 0.005181 | hsa-miR-16-5p |
| NF1 | OTUB2 | 0.006038 | hsa-miR-15a-5p |
| NF1 | EIF2B2 | 0.007427 | hsa-miR-15a-5p |
| NF1 | TMEM245 | 0.009328 | hsa-miR-15a-5p |
| NF1 | SCN8A | 0.009981 | hsa-miR-15a-5p |
| NF1 | SLC3A1 | 0.011507 | hsa-miR-15a-5p |
| NF1 | EPM2AIP1 | 0.001264 | hsa-miR-15a-5p |
| NF1 | ANKRD12 | 0.001044 | hsa-miR-15a-5p |
| NF1 | RP11-370F5.4 | 0.000726 | hsa-miR-543 |
| NF1 | ZNF674-AS1 | 0.000282 | hsa-miR-543 |
| NF1 | LINC01376 | 0.000888 | hsa-miR-543 |
| NF1 | LINC01206 | 0.012316 | hsa-miR-543 |
| NF1 | RP13-516M14.1 | 0.009653 | hsa-miR-543 |
| NF1 | RP11-429B14.4 | 0.008067 | hsa-miR-543 |
| NF1 | RP11-430C1.1 | 0.007535 | hsa-miR-543 |
| NF1 | RP11-48G14.1 | 0.001531 | hsa-miR-543 |
| NF1 | HPS3 | 0.011084 | hsa-miR-543 |
| NF1 | BRCA1 | 0.014794 | hsa-miR-543 |
| NF1 | CSGALNACT2 | 0.008691 | hsa-miR-543 |
| NF1 | MLLT4 | 0.005805 | hsa-miR-543 |
| NF1 | ARV1 | 0.003427 | hsa-miR-543 |
| NF1 | RLF | 0.001432 | hsa-miR-543 |
| NF1 | LCMT2 | 0.009909 | hsa-miR-543 |
| NF1 | PAN2 | 0.009623 | hsa-miR-543 |
| NF1 | HERC4 | 0.000379 | hsa-miR-543 |
| NF1 | GRIK2 | 0.009471 | hsa-miR-543 |
| NF1 | ZNF883 | 0.000392 | hsa-miR-485-5p |
| NF1 | OGFRP1 | 0.009805 | hsa-miR-485-5p |
| NF1 | LINC00671 | 0.000577 | hsa-miR-485-5p |
| NF1 | AC012314.20 | 0.004127 | hsa-miR-485-5p |
| NF1 | RP4-798A10.2 | 0.012404 | hsa-miR-485-5p |
| NF1 | RP11-379B8.1 | 0.006245 | hsa-miR-485-5p |
| NF1 | AL163953.3 | 0.005893 | hsa-miR-485-5p |
| NF1 | RP11-815M8.1 | 0.013137 | hsa-miR-485-5p |
| NF1 | SPATA3-AS1 | 0.001152 | hsa-miR-485-5p |
| NF1 | CTC-573N18.1 | 0.000455 | hsa-miR-485-5p |
| NF1 | RP11-281P23.1 | 0.012933 | hsa-miR-485-5p |
| NF1 | KB-1732A1.1 | 0.013431 | hsa-miR-485-5p |
| NF1 | RP11-172E10.1 | 0.000603 | hsa-miR-485-5p |
| NF1 | RP11-347C12.10 | 0.011673 | hsa-miR-485-5p |
| NF1 | RP11-663N22.1 | 0.009396 | hsa-miR-485-5p |
| NF1 | VPS11 | 0.004943 | hsa-miR-485-5p |
| NF1 | THEM4 | 0.013234 | hsa-miR-485-5p |
| NF1 | GLI2 | 0.001714 | hsa-miR-485-5p |
| NF1 | VHL | 0.001078 | hsa-miR-485-5p |
| NF1 | SZT2 | 0.006868 | hsa-miR-485-5p |
| NF1 | PRKAR2A | 0.000253 | hsa-miR-485-5p |
| NF1 | RPL4 | 0.000574 | hsa-miR-485-5p |
| NF1 | RP11-563J2.2 | 0.01072 | hsa-miR-154-5p |
| NF1 | CEP290 | 0.009315 | hsa-miR-154-5p |
| NF1 | TTC7A | 0.003341 | hsa-miR-154-5p |
| NF1 | TMEM133 | 0.005306 | hsa-miR-154-5p |
| NF1 | RP6-91H8.3 | 0.01109 | hsa-miR-33b-5p |
| NF1 | DIAPH1 | 0.017507 | hsa-miR-33b-5p |
| NF1 | MAGEF1 | 0.015912 | hsa-miR-33b-5p |
| NF1 | SYNE2 | 0.005907 | hsa-miR-33b-5p |
| NF1 | PTPN23 | 0.002257 | hsa-miR-33b-5p |
| NF1 | SRGAP1 | 0.006943 | hsa-miR-33b-5p |
| NF1 | DTX3 | 0.011123 | hsa-miR-33b-5p |
| NF1 | RP11-763E3.1 | 0.011133 | hsa-miR-10a-5p |
| NF1 | ANKH | 0.007566 | hsa-miR-10a-5p |
| NF1 | CTSF | 0.01232 | hsa-miR-10a-5p |
| NF1 | HOOK1 | 0.010619 | hsa-miR-10a-5p |
| NF1 | SMTNL2 | 0.000848 | hsa-miR-10a-5p |
| NF1 | FAM179A | 0.008815 | hsa-miR-10a-5p |
| NF1 | SLC1A2 | 0.007806 | hsa-miR-10a-5p |
| NF1 | LHFPL4 | 0.002345 | hsa-miR-10a-5p |
| NF1 | PDE4A | 0.007301 | hsa-miR-10a-5p |
| NF1 | SNW1 | 0.003363 | hsa-miR-150-5p |
| NF1 | PIGQ | 0.011283 | hsa-miR-150-5p |
| NF1 | RP11-57A1.1 | 0.014616 | hsa-miR-217 |
| NF1 | CLTA | 0.013002 | hsa-miR-217 |
| NF1 | NPTN | 0.013635 | hsa-miR-217 |
| NF1 | MYO9A | 0.001976 | hsa-miR-217 |
| NF1 | DMXL2 | 0.005124 | hsa-miR-217 |
| NF1 | SAP30L | 0.000593 | hsa-miR-217 |
| NF1 | TMPPE | 0.001304 | hsa-miR-217 |
| NF1 | RBM22 | 0.001802 | hsa-miR-217 |
| NF1 | LMO7 | 0.009202 | hsa-miR-217 |
| NF1 | NDFIP1 | 0.001631 | hsa-miR-217 |
| NF1 | ZC3H18 | 0.009258 | hsa-miR-217 |
| NF1 | GPR107 | 0.004817 | hsa-miR-217 |
| NF1 | PTPRD-AS2 | 0.011355 | hsa-miR-10b-5p |
| NF1 | TMEM64 | 0.006822 | hsa-miR-149-5p |
| NF1 | RAB7A | 0.003388 | hsa-miR-149-5p |
| NF1 | POLR1E | 0.012317 | hsa-miR-149-5p |
| NF1 | EP400 | 0.005976 | hsa-miR-149-5p |
| NF1 | E2F6 | 0.009966 | hsa-miR-149-5p |
| NF1 | SREBF2 | 0.00837 | hsa-miR-149-5p |
| NF1 | TMEM140 | 0.008454 | hsa-miR-149-5p |
| NF1 | ABCG1 | 0.01128 | hsa-miR-185-5p |
| NF1 | IK | 0.0083 | hsa-miR-185-5p |
| NF1 | WIZ | 0.008538 | hsa-miR-185-5p |
| NF1 | ZNF75D | 0.009614 | hsa-miR-185-5p |
| NF1 | MRPL45 | 0.001554 | hsa-miR-185-5p |
| NF1 | RP11-278H7.1 | 0.003567 | hsa-miR-301b |
| NF1 | RP11-281H11.1 | 0.010559 | hsa-miR-301b |
| NF1 | PKIA-AS1 | 0.002312 | hsa-miR-301b |
| NF1 | PCDHAC2 | 0.010959 | hsa-miR-301b |
| NF1 | KIAA1217 | 0.00313 | hsa-miR-301b |
| NF1 | ARMC10 | 0.005003 | hsa-miR-301b |
| NF1 | PCDHAC1 | 0.011583 | hsa-miR-301b |
| NF1 | ARL1 | 0.002077 | hsa-miR-301b |
| NF1 | NGFRAP1 | 0.010371 | hsa-miR-301b |
| NF1 | SNPH | 0.003193 | hsa-miR-301b |
| NF1 | PPFIBP2 | 0.003422 | hsa-miR-15b-5p |
| NF1 | WDR11 | 0.006926 | hsa-miR-15b-5p |
| NF1 | ZNF701 | 0.008036 | hsa-miR-145-5p |
| NF1 | AP1S2 | 0.012619 | hsa-miR-145-5p |
| NF1 | EIF2A | 0.003194 | hsa-miR-340-5p |
| NF1 | PMAIP1 | 0.015327 | hsa-miR-340-5p |
| NF1 | LRRN1 | 0.001784 | hsa-miR-340-5p |
| NF1 | FBXO3 | 0.010753 | hsa-miR-340-5p |
| NF1 | TMEM237 | 0.002059 | hsa-miR-340-5p |
| NF1 | IFT46 | 0.004306 | hsa-miR-340-5p |
| NF1 | SRSF12 | 0.009539 | hsa-miR-340-5p |
| NF1 | NCS1 | 0.014553 | hsa-miR-30a-5p |
| NF1 | ANAPC7 | 0.004457 | hsa-miR-30a-5p |
| NF1 | RP11-572C15.5 | 0.00194 | hsa-miR-486-5p |
| NF1 | CTDP1 | 0.010049 | hsa-miR-486-5p |
| NF1 | PRDM5 | 0.005318 | hsa-miR-30b-5p |
| NF1 | AMPH | 0.005983 | hsa-miR-32-5p |
| NF1 | EXOC3 | 0.000106 | hsa-miR-32-5p |
| NF1 | ITFG1 | 0.00234 | hsa-miR-374b-5p |
| NF1 | PRMT3 | 0.002177 | hsa-miR-374b-5p |
| NF1 | ZNF793 | 0.0002 | hsa-miR-374b-5p |
| NF1 | RUNDC1 | 0.000261 | hsa-miR-374b-5p |
| NF1 | VAPB | 0.010638 | hsa-miR-374b-5p |
| NF1 | TCEB1 | 0.004894 | hsa-miR-374b-5p |
| NF1 | PPP6R1 | 0.004334 | hsa-miR-374b-5p |
| NF1 | HERC1 | 0.002467 | hsa-miR-374b-5p |
| NF1 | EIF5 | 0.006579 | hsa-miR-374b-5p |
| NF1 | CPSF2 | 0.009148 | hsa-miR-361-5p |
| NF1 | SND1 | 0.012457 | hsa-miR-361-5p |
| NF1 | AIG1 | 0.002125 | hsa-miR-361-5p |
| NF1 | AC084082.3 | 0.003679 | hsa-miR-424-5p |
| PTEN | SCAMP1 | 0.006661 | hsa-miR-30e-5p |
| PTEN | RP1-151F17.2 | 0.007451 | hsa-miR-30e-5p |
| PTEN | SECISBP2 | 0.002078 | hsa-miR-30e-5p |
| PTEN | ORC2 | 0.005255 | hsa-miR-30e-5p |
| PTEN | TMEM170B | 0.009314 | hsa-miR-30e-5p |
| PTEN | SUZ12 | 0.010214 | hsa-miR-30e-5p |
| PTEN | CRKL | 0.008256 | hsa-miR-30e-5p |
| PTEN | DHX36 | 0.001958 | hsa-miR-30e-5p |
| PTEN | ZFX | 0.008094 | hsa-miR-30e-5p |
| PTEN | YES1 | 0.013202 | hsa-miR-30e-5p |
| PTEN | SGCB | 0.007416 | hsa-miR-30e-5p |
| PTEN | TUBGCP5 | 0.002053 | hsa-miR-30e-5p |
| PTEN | RPS6KB1 | 0.003576 | hsa-miR-30e-5p |
| PTEN | ARHGEF2 | 0.009028 | hsa-miR-30e-5p |
| PTEN | MLXIP | 0.010529 | hsa-miR-30e-5p |
| PTEN | C4orf46 | 0.004363 | hsa-miR-30e-5p |
| PTEN | LCORL | 0.003914 | hsa-miR-30e-5p |
| PTEN | FAM160B1 | 0.002954 | hsa-miR-30e-5p |
| PTEN | UBE2I | 0.010283 | hsa-miR-30e-5p |
| PTEN | FAHD1 | 0.009045 | hsa-miR-30e-5p |
| PTEN | AKAP1 | 0.005688 | hsa-miR-30e-5p |
| PTEN | GNPDA2 | 0.003085 | hsa-miR-30e-5p |
| PTEN | ZNF711 | 0.002834 | hsa-miR-30e-5p |
| PTEN | NIF3L1 | 0.007951 | hsa-miR-30e-5p |
| PTEN | CLNS1A | 0.010676 | hsa-miR-30e-5p |
| PTEN | LIFR | 0.010688 | hsa-miR-30e-5p |
| PTEN | HIBCH | 0.015604 | hsa-miR-30e-5p |
| PTEN | BUB3 | 0.004032 | hsa-miR-30e-5p |
| PTEN | LRRC58 | 0.007767 | hsa-miR-30e-5p |
| PTEN | PELO | 0.006247 | hsa-miR-30e-5p |
| PTEN | CASP3 | 0.003461 | hsa-miR-30e-5p |
| PTEN | PPM1D | 0.015128 | hsa-miR-30e-5p |
| PTEN | DDIT4 | 0.003777 | hsa-miR-30e-5p |
| PTEN | MKRN3 | 0.008004 | hsa-miR-30e-5p |
| PTEN | RCOR1 | 0.009081 | hsa-miR-30e-5p |
| PTEN | PSIP1 | 0.010235 | hsa-miR-30e-5p |
| PTEN | FLVCR2 | 0.00602 | hsa-miR-30e-5p |
| PTEN | STAG2 | 0.003118 | hsa-miR-30e-5p |
| PTEN | ANKHD1 | 0.007592 | hsa-miR-30e-5p |
| PTEN | NRP1 | 0.004445 | hsa-miR-30e-5p |
| PTEN | EAF1 | 0.001811 | hsa-miR-30e-5p |
| PTEN | MAP4K4 | 0.016886 | hsa-miR-30e-5p |
| PTEN | AQR | 0.007585 | hsa-miR-30e-5p |
| PTEN | KCTD3 | 0.002511 | hsa-miR-30e-5p |
| PTEN | BCOR | 0.006316 | hsa-miR-30e-5p |
| PTEN | MBD1 | 0.006075 | hsa-miR-30e-5p |
| PTEN | TSHZ1 | 0.005914 | hsa-miR-30e-5p |
| PTEN | SLC35F1 | 0.007789 | hsa-miR-30e-5p |
| PTEN | WDR26 | 0.006737 | hsa-miR-30e-5p |
| PTEN | FLVCR1 | 0.011277 | hsa-miR-30e-5p |
| PTEN | AMMECR1L | 0.00986 | hsa-miR-30e-5p |
| PTEN | ATAD1 | 0.01169 | hsa-miR-30e-5p |
| PTEN | CBLB | 0.006485 | hsa-miR-30e-5p |
| PTEN | TUBB3 | 0.008583 | hsa-miR-30e-5p |
| PTEN | CPEB4 | 0.011971 | hsa-miR-30e-5p |
| PTEN | COG2 | 0.005984 | hsa-miR-30e-5p |
| PTEN | RNF122 | 0.009936 | hsa-miR-30e-5p |
| PTEN | LYRM7 | 0.001535 | hsa-miR-30e-5p |
| PTEN | VCAN | 0.000832 | hsa-miR-30e-5p |
| PTEN | NAA30 | 0.006948 | hsa-miR-30e-5p |
| PTEN | EPC2 | 0.006308 | hsa-miR-30e-5p |
| PTEN | C9orf85 | 0.008769 | hsa-miR-30e-5p |
| PTEN | KLF10 | 0.003458 | hsa-miR-30e-5p |
| PTEN | PRR14L | 0.004456 | hsa-miR-30e-5p |
| PTEN | RSF1 | 0.011375 | hsa-miR-30e-5p |
| PTEN | CBX1 | 0.013979 | hsa-miR-30e-5p |
| PTEN | ZNF597 | 0.006532 | hsa-miR-30e-5p |
| PTEN | PDE4D | 0.010204 | hsa-miR-30e-5p |
| PTEN | MRPS14 | 0.003354 | hsa-miR-30e-5p |
| PTEN | PANK1 | 0.014705 | hsa-miR-30e-5p |
| PTEN | BTBD1 | 0.011224 | hsa-miR-30c-5p |
| PTEN | SLC11A2 | 0.00266 | hsa-miR-30c-5p |
| PTEN | VAT1 | 0.007177 | hsa-miR-30c-5p |
| PTEN | RNMT | 0.002753 | hsa-miR-30c-5p |
| PTEN | FGD6 | 0.008616 | hsa-miR-30c-5p |
| PTEN | SLC9A8 | 0.007232 | hsa-miR-30c-5p |
| PTEN | OAT | 0.016824 | hsa-miR-30c-5p |
| PTEN | LSS | 0.011995 | hsa-miR-30c-5p |
| PTEN | SRFBP1 | 0.006627 | hsa-miR-30c-5p |
| PTEN | TRIP12 | 0.002265 | hsa-miR-30c-5p |
| PTEN | CLOCK | 0.008072 | hsa-miR-30c-5p |
| PTEN | NSF | 0.007709 | hsa-miR-30c-5p |
| PTEN | ATP8B1 | 0.013218 | hsa-miR-30c-5p |
| PTEN | MED28 | 0.009433 | hsa-miR-30c-5p |
| PTEN | ASPH | 0.008203 | hsa-miR-30c-5p |
| PTEN | NIN | 0.004358 | hsa-miR-30c-5p |
| PTEN | AKAP11 | 0.010738 | hsa-miR-30c-5p |
| PTEN | ENAH | 0.000642 | hsa-miR-30c-5p |
| PTEN | G3BP1 | 0.005512 | hsa-miR-30c-5p |
| PTEN | YWHAZ | 0.003547 | hsa-miR-30c-5p |
| PTEN | MAP3K1 | 0.009753 | hsa-miR-30c-5p |
| PTEN | DEGS1 | 0.008494 | hsa-miR-30c-5p |
| PTEN | ZNF780B | 0.001649 | hsa-miR-30c-5p |
| PTEN | PPAT | 0.015541 | hsa-miR-30c-5p |
| PTEN | CLUAP1 | 0.011547 | hsa-miR-30c-5p |
| PTEN | TMEM55A | 0.006964 | hsa-miR-30c-5p |
| PTEN | PPP3CB | 0.004937 | hsa-miR-30c-5p |
| PTEN | FAS | 0.011013 | hsa-miR-30c-5p |
| PTEN | PUM1 | 0.006032 | hsa-miR-30c-5p |
| PTEN | TRPM7 | 0.00188 | hsa-miR-30c-5p |
| PTEN | ATP6V1H | 0.009574 | hsa-miR-30c-5p |
| PTEN | CLTC | 0.003144 | hsa-miR-30c-5p |
| PTEN | RAB5B | 0.001278 | hsa-miR-30c-5p |
| PTEN | ELK4 | 0.010012 | hsa-miR-30c-5p |
| PTEN | NCKAP1 | 0.00574 | hsa-miR-30c-5p |
| PTEN | TPMT | 0.002744 | hsa-miR-30c-5p |
| PTEN | BAZ1B | 0.007553 | hsa-miR-30c-5p |
| PTEN | TJP1 | 0.012505 | hsa-miR-30c-5p |
| PTEN | NUP153 | 0.002934 | hsa-miR-30c-5p |
| PTEN | C12orf29 | 0.003428 | hsa-miR-30c-5p |
| PTEN | JRKL | 0.006528 | hsa-miR-30c-5p |
| PTEN | GALNT10 | 0.003721 | hsa-miR-30c-5p |
| PTEN | RP11-923I11.6 | 0.004297 | hsa-miR-186-5p |
| PTEN | GOSR1 | 0.006714 | hsa-miR-186-5p |
| PTEN | WDR5B | 0.003061 | hsa-miR-186-5p |
| PTEN | BASP1 | 0.008163 | hsa-miR-186-5p |
| PTEN | QTRTD1 | 0.009277 | hsa-miR-186-5p |
| PTEN | ATF2 | 0.003958 | hsa-miR-186-5p |
| PTEN | ATE1 | 0.005665 | hsa-miR-186-5p |
| PTEN | CDKN1A | 0.013661 | hsa-miR-186-5p |
| PTEN | FCF1 | 0.007776 | hsa-miR-186-5p |
| PTEN | MTSS1L | 0.010676 | hsa-miR-186-5p |
| PTEN | CGGBP1 | 0.008827 | hsa-miR-186-5p |
| PTEN | SBNO1 | 0.00749 | hsa-miR-186-5p |
| PTEN | TIGD1 | 0.009399 | hsa-miR-186-5p |
| PTEN | CNOT4 | 0.000955 | hsa-miR-186-5p |
| PTEN | SDCBP | 0.012906 | hsa-miR-186-5p |
| PTEN | PAICS | 0.009388 | hsa-miR-186-5p |
| PTEN | TXNRD1 | 0.002144 | hsa-miR-186-5p |
| PTEN | SPOPL | 0.005443 | hsa-miR-186-5p |
| PTEN | SLC1A1 | 0.010611 | hsa-miR-186-5p |
| PTEN | NCOA1 | 0.001185 | hsa-miR-186-5p |
| PTEN | KLF7 | 0.009415 | hsa-miR-186-5p |
| PTEN | PHF12 | 0.003577 | hsa-miR-186-5p |
| PTEN | MTPN | 0.008844 | hsa-miR-186-5p |
| PTEN | CHD6 | 0.008197 | hsa-miR-186-5p |
| PTEN | ARMC1 | 0.002238 | hsa-miR-186-5p |
| PTEN | CYFIP2 | 0.009104 | hsa-miR-186-5p |
| PTEN | METTL3 | 0.009993 | hsa-miR-186-5p |
| PTEN | ZADH2 | 0.007629 | hsa-miR-186-5p |
| PTEN | ATF7 | 0.006455 | hsa-miR-186-5p |
| PTEN | GTF3C1 | 0.011601 | hsa-miR-186-5p |
| PTEN | ZFR | 0.001454 | hsa-miR-186-5p |
| PTEN | FBXO22 | 0.009723 | hsa-miR-186-5p |
| PTEN | ST3GAL2 | 0.013437 | hsa-miR-186-5p |
| PTEN | DDX21 | 0.002191 | hsa-miR-186-5p |
| PTEN | ELF2 | 0.004405 | hsa-miR-186-5p |
| PTEN | NCOA4 | 0.00842 | hsa-miR-186-5p |
| PTEN | DHX29 | 0.012316 | hsa-miR-186-5p |
| PTEN | NFU1 | 0.00521 | hsa-miR-186-5p |
| PTEN | HNRNPU | 0.008881 | hsa-miR-186-5p |
| PTEN | MRPL3 | 0.002426 | hsa-miR-186-5p |
| PTEN | DCAF17 | 0.002966 | hsa-miR-186-5p |
| PTEN | DERL1 | 0.007127 | hsa-miR-186-5p |
| PTEN | TOP1 | 0.006437 | hsa-miR-186-5p |
| PTEN | UBE2Q1 | 0.001283 | hsa-miR-186-5p |
| PTEN | RFWD2 | 0.006892 | hsa-miR-186-5p |
| PTEN | CASC4 | 0.006612 | hsa-miR-186-5p |
| PTEN | PAFAH1B1 | 0.003812 | hsa-miR-186-5p |
| PTEN | KRIT1 | 0.000214 | hsa-miR-186-5p |
| PTEN | FADS1 | 0.004362 | hsa-miR-186-5p |
| PTEN | TMOD2 | 0.013104 | hsa-miR-186-5p |
| PTEN | AFAP1L1 | 0.01637 | hsa-miR-186-5p |
| PTEN | SYS1 | 0.014507 | hsa-miR-186-5p |
| PTEN | ISCA1 | 0.013682 | hsa-miR-186-5p |
| PTEN | SLC30A6 | 0.008589 | hsa-miR-186-5p |
| PTEN | ITGAV | 0.00538 | hsa-miR-186-5p |
| PTEN | SEC61A1 | 0.010464 | hsa-miR-186-5p |
| PTEN | WWC3 | 0.003847 | hsa-miR-186-5p |
| PTEN | GGNBP2 | 0.004779 | hsa-miR-186-5p |
| PTEN | SHPRH | 0.003209 | hsa-miR-186-5p |
| PTEN | SOCS4 | 0.009656 | hsa-miR-186-5p |
| PTEN | NRP2 | 0.001313 | hsa-miR-186-5p |
| PTEN | UBR5 | 0.010245 | hsa-miR-186-5p |
| PTEN | TACC2 | 0.000612 | hsa-miR-186-5p |
| PTEN | YIPF6 | 0.011395 | hsa-miR-186-5p |
| PTEN | CMTM8 | 0.008835 | hsa-miR-186-5p |
| PTEN | ARPC1A | 0.007242 | hsa-miR-186-5p |
| PTEN | GABPA | 0.00039 | hsa-miR-186-5p |
| PTEN | CDK13 | 0.011616 | hsa-miR-186-5p |
| PTEN | SLCO5A1 | 0.000568 | hsa-miR-186-5p |
| PTEN | CBLL1 | 0.004999 | hsa-miR-186-5p |
| PTEN | PPP1CC | 0.009813 | hsa-miR-186-5p |
| PTEN | BCAP29 | 0.016537 | hsa-miR-186-5p |
| PTEN | SRPK2 | 0.008153 | hsa-miR-186-5p |
| PTEN | CD24 | 0.001686 | hsa-miR-186-5p |
| PTEN | RPL15 | 0.002421 | hsa-miR-186-5p |
| PTEN | RP11-147L13.8 | 0.008979 | hsa-miR-181b-5p |
| PTEN | TGFBR3 | 0.001446 | hsa-miR-181b-5p |
| PTEN | NAB1 | 0.011377 | hsa-miR-181b-5p |
| PTEN | EYA1 | 0.007347 | hsa-miR-181b-5p |
| PTEN | IRF1 | 0.003808 | hsa-miR-181b-5p |
| PTEN | TLE3 | 0.00335 | hsa-miR-181b-5p |
| PTEN | MAP3K14 | 0.00103 | hsa-miR-181b-5p |
| PTEN | TMEM9B | 0.008172 | hsa-miR-181b-5p |
| PTEN | TBX3 | 0.012174 | hsa-miR-181b-5p |
| PTEN | LIF | 0.00655 | hsa-miR-181b-5p |
| PTEN | SEC24C | 0.009025 | hsa-miR-181b-5p |
| PTEN | ADAMTS20 | 0.007816 | hsa-miR-181b-5p |
| PTEN | OGFRL1 | 0.011778 | hsa-miR-181b-5p |
| PTEN | SHMT1 | 0.002496 | hsa-miR-181b-5p |
| PTEN | ZNF487 | 0.006686 | hsa-miR-181b-5p |
| PTEN | CARD8 | 0.000229 | hsa-miR-181b-5p |
| PTEN | FAM117A | 0.008524 | hsa-miR-181b-5p |
| PTEN | PLEKHF2 | 0.009503 | hsa-miR-181b-5p |
| PTEN | ELK3 | 0.000929 | hsa-miR-181b-5p |
| PTEN | PRR3 | 0.011284 | hsa-miR-181b-5p |
| PTEN | SPECC1L | 0.013235 | hsa-miR-181b-5p |
| PTEN | LIMK2 | 0.01104 | hsa-miR-181b-5p |
| PTEN | SNHG16 | 0.014398 | hsa-miR-146b-5p |
| PTEN | RP11-15A1.3 | 0.002501 | hsa-miR-146b-5p |
| PTEN | ATG13 | 0.001956 | hsa-miR-146b-5p |
| PTEN | ZNF676 | 0.006817 | hsa-miR-146b-5p |
| PTEN | LDLRAD3 | 0.005367 | hsa-miR-146b-5p |
| PTEN | DDX6 | 0.0015 | hsa-miR-146b-5p |
| PTEN | BEND4 | 0.00091 | hsa-miR-146b-5p |
| PTEN | CLCN6 | 0.001075 | hsa-miR-146b-5p |
| PTEN | MANEAL | 0.01577 | hsa-miR-146b-5p |
| PTEN | NFXL1 | 0.006902 | hsa-miR-146b-5p |
| PTEN | SLC9A6 | 0.006259 | hsa-miR-146b-5p |
| PTEN | C14orf1 | 0.006051 | hsa-miR-146b-5p |
| PTEN | STARD7 | 0.005649 | hsa-miR-146b-5p |
| PTEN | ASAP1 | 0.003757 | hsa-miR-146b-5p |
| PTEN | RP11-276H19.2 | 0.014681 | hsa-miR-26a-5p |
| PTEN | RP11-95O2.1 | 0.002865 | hsa-miR-26a-5p |
| PTEN | SPPL3 | 0.008082 | hsa-miR-26a-5p |
| PTEN | NOP56 | 0.00997 | hsa-miR-26a-5p |
| PTEN | ZNF300 | 0.006459 | hsa-miR-26a-5p |
| PTEN | SLITRK5 | 0.013883 | hsa-miR-26a-5p |
| PTEN | DAPK1 | 0.003905 | hsa-miR-26a-5p |
| PTEN | MAD2L1 | 0.003368 | hsa-miR-26a-5p |
| PTEN | ACADSB | 0.003331 | hsa-miR-26a-5p |
| PTEN | LARS | 0.010809 | hsa-miR-26a-5p |
| PTEN | RFK | 0.00888 | hsa-miR-26a-5p |
| PTEN | FBXO3 | 0.002802 | hsa-miR-26a-5p |
| PTEN | SGPL1 | 0.005857 | hsa-miR-26a-5p |
| PTEN | TRIO | 0.00065 | hsa-miR-26a-5p |
| PTEN | NOB1 | 0.01111 | hsa-miR-26a-5p |
| PTEN | EXOC8 | 0.002761 | hsa-miR-26a-5p |
| PTEN | CLASP2 | 0.007001 | hsa-miR-26a-5p |
| PTEN | PELI2 | 0.00719 | hsa-miR-26a-5p |
| PTEN | ATP9A | 0.010279 | hsa-miR-26a-5p |
| PTEN | VANGL2 | 0.003518 | hsa-miR-26a-5p |
| PTEN | TDP1 | 0.001659 | hsa-miR-26a-5p |
| PTEN | KIAA0753 | 0.000997 | hsa-miR-26a-5p |
| PTEN | GABPB1 | 0.007295 | hsa-miR-26a-5p |
| PTEN | EHD1 | 0.012459 | hsa-miR-26a-5p |
| PTEN | PIKFYVE | 0.005427 | hsa-miR-26a-5p |
| PTEN | HDAC4 | 0.002088 | hsa-miR-26a-5p |
| PTEN | PGBD5 | 0.01385 | hsa-miR-20a-5p |
| PTEN | AKAP12 | 0.002323 | hsa-miR-20a-5p |
| PTEN | RTN4 | 0.001066 | hsa-miR-20a-5p |
| PTEN | ST8SIA4 | 0.007335 | hsa-miR-20a-5p |
| PTEN | PLSCR4 | 0.00911 | hsa-miR-20a-5p |
| PTEN | PLEKHM1 | 0.004086 | hsa-miR-20a-5p |
| PTEN | ASB9 | 0.014159 | hsa-miR-20a-5p |
| PTEN | ZNF460 | 0.001564 | hsa-miR-20a-5p |
| PTEN | SLC35F5 | 0.007019 | hsa-miR-20a-5p |
| PTEN | PISD | 0.009855 | hsa-miR-20a-5p |
| PTEN | HOOK1 | 0.005418 | hsa-miR-20a-5p |
| PTEN | RAB11FIP4 | 0.011939 | hsa-miR-20a-5p |
| PTEN | LRCH1 | 0.008805 | hsa-miR-20a-5p |
| PTEN | PRKAR2B | 0.012982 | hsa-miR-20a-5p |
| PTEN | TLE4 | 0.009177 | hsa-miR-20a-5p |
| PTEN | PRDM12 | 0.002643 | hsa-miR-20a-5p |
| PTEN | SORL1 | 0.017078 | hsa-miR-20a-5p |
| PTEN | ARHGEF3 | 0.007927 | hsa-miR-20a-5p |
| PTEN | CCNYL1 | 0.010588 | hsa-miR-20a-5p |
| PTEN | GOLGB1 | 0.013633 | hsa-miR-20a-5p |
| PTEN | SYAP1 | 0.014184 | hsa-miR-20a-5p |
| PTEN | MET | 0.013291 | hsa-miR-20a-5p |
| PTEN | GAK | 0.011871 | hsa-miR-20a-5p |
| PTEN | FAT4 | 0.000651 | hsa-miR-20a-5p |
| PTEN | DNAJC16 | 0.012661 | hsa-miR-20a-5p |
| PTEN | MINK1 | 0.007082 | hsa-miR-20a-5p |
| PTEN | PPP3R1 | 0.008275 | hsa-miR-20a-5p |
| PTEN | SDCCAG8 | 0.018505 | hsa-miR-20a-5p |
| PTEN | SNX12 | 0.004325 | hsa-miR-20a-5p |
| PTEN | ST6GALNAC3 | 0.012312 | hsa-miR-20a-5p |
| PTEN | TGFB2 | 0.009341 | hsa-miR-20a-5p |
| PTEN | CYP26B1 | 0.00337 | hsa-miR-20a-5p |
| PTEN | GALNT4 | 0.012319 | hsa-miR-20a-5p |
| PTEN | KIAA0232 | 0.005054 | hsa-miR-20a-5p |
| PTEN | PAPSS2 | 0.001896 | hsa-miR-20a-5p |
| PTEN | SPTBN1 | 0.000793 | hsa-miR-20a-5p |
| PTEN | LYST | 0.005056 | hsa-miR-20a-5p |
| PTEN | COL4A1 | 0.006638 | hsa-miR-20a-5p |
| PTEN | TRIM8 | 0.007661 | hsa-miR-20a-5p |
| PTEN | CTSA | 0.013271 | hsa-miR-20a-5p |
| PTEN | SRSF4 | 0.011646 | hsa-miR-20a-5p |
| PTEN | ITPR1 | 0.003893 | hsa-miR-20a-5p |
| PTEN | SLC40A1 | 0.006986 | hsa-miR-20a-5p |
| PTEN | ZBTB4 | 0.000836 | hsa-miR-20a-5p |
| PTEN | USP33 | 0.003342 | hsa-miR-20a-5p |
| PTEN | LINC00237 | 0.012514 | hsa-miR-33b-5p |
| PTEN | LINC00899 | 0.005246 | hsa-miR-33b-5p |
| PTEN | UQCRC2 | 0.012849 | hsa-miR-33b-5p |
| PTEN | CDCA2 | 0.00455 | hsa-miR-33b-5p |
| PTEN | NOMO3 | 0.003283 | hsa-miR-33b-5p |
| PTEN | PARD6B | 0.009398 | hsa-miR-33b-5p |
| PTEN | CDK7 | 0.004696 | hsa-miR-33b-5p |
| PTEN | DNAJC10 | 0.008773 | hsa-miR-33b-5p |
| PTEN | RPF1 | 0.008807 | hsa-miR-33b-5p |
| PTEN | DOK4 | 0.003332 | hsa-miR-33b-5p |
| PTEN | C18orf21 | 0.010473 | hsa-miR-33b-5p |
| PTEN | ACTB | 0.007671 | hsa-miR-33b-5p |
| PTEN | ZNF639 | 0.01265 | hsa-miR-33b-5p |
| PTEN | SIPA1L2 | 0.00898 | hsa-miR-33b-5p |
| PTEN | CTC-448F2.4 | 0.007239 | hsa-miR-26b-5p |
| PTEN | EIF4E | 0.0027 | hsa-miR-26b-5p |
| PTEN | COL12A1 | 0.013748 | hsa-miR-26b-5p |
| PTEN | GLS | 0.010934 | hsa-miR-26b-5p |
| PTEN | C9orf91 | 0.012259 | hsa-miR-26b-5p |
| PTEN | S100PBP | 0.013324 | hsa-miR-26b-5p |
| PTEN | TANC1 | 0.01188 | hsa-miR-26b-5p |
| PTEN | LINC01378 | 0.013446 | hsa-miR-155-5p |
| PTEN | PRPF38A | 0.002829 | hsa-miR-155-5p |
| PTEN | MAPRE2 | 0.011228 | hsa-miR-155-5p |
| PTEN | SLC23A2 | 0.001676 | hsa-miR-155-5p |
| PTEN | RPL14 | 0.011243 | hsa-miR-155-5p |
| PTEN | TSPAN14 | 0.000389 | hsa-miR-155-5p |
| PTEN | IPCEF1 | 0.006602 | hsa-miR-155-5p |
| PTEN | KIAA1429 | 0.007726 | hsa-miR-155-5p |
| PTEN | HSDL1 | 0.004098 | hsa-miR-155-5p |
| PTEN | PPP2R2A | 0.006328 | hsa-miR-155-5p |
| PTEN | FOXP2 | 0.013977 | hsa-miR-155-5p |
| PTEN | HSD17B4 | 0.001616 | hsa-miR-155-5p |
| PTEN | RP11-139I14.2 | 0.002642 | hsa-miR-425-5p |
| PTEN | RP13-631K18.2 | 0.004332 | hsa-miR-425-5p |
| PTEN | RP3-454B23.1 | 0.003395 | hsa-miR-425-5p |
| PTEN | LINC00645 | 0.008953 | hsa-miR-425-5p |
| PTEN | RP11-325E5.1 | 0.00608 | hsa-miR-425-5p |
| PTEN | RP11-2E17.2 | 0.005245 | hsa-miR-425-5p |
| PTEN | VPS33B | 0.006208 | hsa-miR-425-5p |
| PTEN | TNIP1 | 0.013789 | hsa-miR-425-5p |
| PTEN | EXOSC9 | 0.004492 | hsa-miR-425-5p |
| PTEN | NCOA6 | 0.012232 | hsa-miR-425-5p |
| PTEN | TMED4 | 0.006026 | hsa-miR-425-5p |
| PTEN | ALG10B | 0.00403 | hsa-miR-425-5p |
| PTEN | RAE1 | 0.011597 | hsa-miR-425-5p |
| PTEN | RP11-456K23.1 | 0.00131 | hsa-let-7g-5p |
| PTEN | ZNF799 | 0.007905 | hsa-let-7g-5p |
| PTEN | GALNT13 | 0.008894 | hsa-let-7g-5p |
| PTEN | ZCCHC9 | 0.013322 | hsa-let-7g-5p |
| PTEN | EHMT2 | 0.00865 | hsa-let-7g-5p |
| PTEN | ATG10 | 0.011648 | hsa-let-7g-5p |
| PTEN | TRIM26 | 0.01082 | hsa-let-7g-5p |
| PTEN | ZNF443 | 0.01231 | hsa-let-7g-5p |
| PTEN | SUOX | 0.004437 | hsa-let-7g-5p |
| PTEN | TRIM16L | 0.011389 | hsa-let-7g-5p |
| PTEN | CHD4 | 0.008679 | hsa-let-7g-5p |
| PTEN | PXMP4 | 0.0104 | hsa-let-7g-5p |
| PTEN | SNX4 | 0.00925 | hsa-let-7g-5p |
| PTEN | C6orf89 | 0.007795 | hsa-let-7g-5p |
| PTEN | TCEANC | 0.012973 | hsa-let-7g-5p |
| PTEN | KIAA0895L | 0.004651 | hsa-let-7g-5p |
| PTEN | ZNF10 | 0.002064 | hsa-let-7g-5p |
| PTEN | C5orf15 | 0.000317 | hsa-let-7g-5p |
| PTEN | ZNF529 | 0.007815 | hsa-miR-30a-5p |
| PTEN | DCP2 | 0.00717 | hsa-miR-30a-5p |
| PTEN | KDM5A | 0.008139 | hsa-miR-30a-5p |
| PTEN | PIAS1 | 0.007948 | hsa-miR-320a |
| PTEN | GFOD1 | 0.007906 | hsa-miR-320a |
| PTEN | ASAH2B | 0.000121 | hsa-miR-320a |
| PTEN | MYOF | 0.00212 | hsa-miR-320a |
| PTEN | KLF5 | 0.009911 | hsa-miR-320a |
| PTEN | VHL | 0.00825 | hsa-miR-320a |
| PTEN | GLMN | 0.010274 | hsa-miR-320a |
| PTEN | RAI2 | 0.016595 | hsa-miR-320a |
| PTEN | DCUN1D1 | 0.0026 | hsa-miR-320a |
| PTEN | NUAK1 | 0.00036 | hsa-miR-320a |
| PTEN | DLG2 | 0.002701 | hsa-miR-320a |
| PTEN | KIAA0368 | 0.006419 | hsa-miR-320a |
| PTEN | DNAJB5 | 0.004916 | hsa-miR-320a |
| PTEN | ZNF585B | 0.014662 | hsa-miR-30b-5p |
| PTEN | P4HA2 | 0.002309 | hsa-miR-30b-5p |
| PTEN | MOV10 | 0.006658 | hsa-miR-30b-5p |
| PTEN | WWTR1 | 0.004162 | hsa-miR-30b-5p |
| PTEN | ZNF521 | 0.017601 | hsa-miR-30b-5p |
| PTEN | EPDR1 | 0.004772 | hsa-miR-30b-5p |
| PTEN | TDRD7 | 0.012426 | hsa-miR-30b-5p |
| PTEN | TMEM64 | 0.001804 | hsa-miR-30b-5p |
| PTEN | LYN | 0.009251 | hsa-miR-30b-5p |
| PTEN | NFIL3 | 0.007704 | hsa-miR-374b-5p |
| PTEN | MRPL32 | 0.000707 | hsa-miR-374b-5p |
| PTEN | CIITA | 0.006703 | hsa-miR-374b-5p |
| PTEN | LLPH | 0.003925 | hsa-miR-374b-5p |
| PTEN | MYO1B | 0.012934 | hsa-miR-374b-5p |
| PTEN | THSD7B | 0.010407 | hsa-miR-374b-5p |
| PTEN | FRMPD4 | 0.00502 | hsa-miR-374b-5p |
| PTEN | ZNF701 | 0.002966 | hsa-miR-374b-5p |
| PTEN | FBXO48 | 0.005438 | hsa-miR-374b-5p |
| PTEN | SDHD | 0.006629 | hsa-miR-374b-5p |
| PTEN | PSMD9 | 0.011788 | hsa-miR-374b-5p |
| PTEN | COX15 | 0.002813 | hsa-miR-374b-5p |
| PTEN | HMCN1 | 0.011734 | hsa-miR-374b-5p |
| PTEN | CCDC127 | 0.000106 | hsa-miR-374b-5p |
| PTEN | FASTKD5 | 0.00176 | hsa-miR-374b-5p |
| PTEN | PRRC2C | 0.002186 | hsa-miR-374b-5p |
| PTEN | CHCHD4 | 0.004567 | hsa-miR-374b-5p |
| PTEN | ASNSD1 | 0.005717 | hsa-miR-374b-5p |
| PTEN | RP11-498C9.17 | 0.007826 | hsa-miR-106a-5p |
| PTEN | RP11-66D17.3 | 0.013689 | hsa-miR-106a-5p |
| PTEN | RNF4 | 0.00861 | hsa-miR-106a-5p |
| PTEN | HSD17B11 | 0.000183 | hsa-miR-106a-5p |
| PTEN | ZNF239 | 0.010935 | hsa-miR-106a-5p |
| PTEN | ZNF362 | 0.008682 | hsa-miR-106a-5p |
| PTEN | TLX1 | 0.009485 | hsa-miR-106a-5p |
| PTEN | HERC6 | 0.011335 | hsa-miR-106a-5p |
| PTEN | LIX1L | 0.014157 | hsa-miR-106a-5p |
